# Improving virtual screening of G protein-coupled receptors via ligand-directed modeling

**DOI:** 10.1371/journal.pcbi.1005819

**Published:** 2017-11-13

**Authors:** Thomas Coudrat, John Simms, Arthur Christopoulos, Denise Wootten, Patrick M. Sexton

**Affiliations:** 1 Drug Discovery Biology and Department of Pharmacology, Monash Institute of Pharmaceutical Sciences, Monash University, Parkville, Victoria, Australia; 2 School of Life and Health Sciences, Aston University, Birmingham, United Kingdom; Icahn School of Medicine at Mount Sinai, UNITED STATES

## Abstract

G protein-coupled receptors (GPCRs) play crucial roles in cell physiology and pathophysiology. There is increasing interest in using structural information for virtual screening (VS) of libraries and for structure-based drug design to identify novel agonist or antagonist leads. However, the sparse availability of experimentally determined GPCR/ligand complex structures with diverse ligands impedes the application of structure-based drug design (SBDD) programs directed to identifying new molecules with a select pharmacology. In this study, we apply ligand-directed modeling (LDM) to available GPCR X-ray structures to improve VS performance and selectivity towards molecules of specific pharmacological profile. The described method refines a GPCR binding pocket conformation using a single known ligand for that GPCR. The LDM method is a computationally efficient, iterative workflow consisting of protein sampling and ligand docking. We developed an extensive benchmark comparing LDM-refined binding pockets to GPCR X-ray crystal structures across seven different GPCRs bound to a range of ligands of different chemotypes and pharmacological profiles. LDM-refined models showed improvement in VS performance over origin X-ray crystal structures in 21 out of 24 cases. In all cases, the LDM-refined models had superior performance in enriching for the chemotype of the refinement ligand. This likely contributes to the LDM success in all cases of inhibitor-bound to agonist-bound binding pocket refinement, a key task for GPCR SBDD programs. Indeed, agonist ligands are required for a plethora of GPCRs for therapeutic intervention, however GPCR X-ray structures are mostly restricted to their inactive inhibitor-bound state.

## Author summary

G protein-coupled receptors (GPCRs) are a major target for drug discovery. These receptors are highly dynamic membrane proteins, and have had limited tractability using with biophysical screens that are widely adopted for globular protein targets. Thus, structure-based virtual screening (SBVS) holds great promise as a complement to physical screening for rational design of novel drugs. Indeed, the increasing number of atomic-detail GPCR X-ray crystal structures has coincided with an increase in prospective SBVS studies that have identified novel compounds. However, experimentally solved GPCR structures do not meet the full demand for SBVS, as the GPCR structural landscape is incomplete, lacking both in coverage of available GPCRs, and diversity in both receptor conformations and the chemistry of co-crystalised ligands. Here we present a novel computational GPCR binding pocket refinement method that can generate predictive GPCR/ligand complexes with improved SBVS performance. This ligand-directed modeling workflow uses parallel processing and efficient algorithms to search the GPCR/ligand conformational space faster and more efficiently than the widely used protein refinement method molecular dynamics. In this study, the resulting models are evaluated both structurally, and in retrospective SBVS. We demonstrate improved performance of refined models over their starting structures in the majority of our test cases.

## Introduction

G protein-coupled receptors (GPCRs) are the largest protein superfamily in mammalian genomes [1,2], encompassing close to 800 human genes that play key roles in modulating tissue and cell physiology and homoeostasis [3]. Consequently, GPCRs are currently targeted by over 30% of all prescription pharmaceuticals on the market [4]. GPCRs all share a common transmembrane (TM) fold [5] and the superfamily is organised into four main classes according to the A-F classification system [6,7]. Their function is modulated by a wide variety of activity modulators, including peptide and non-peptide neurotransmitters and hormones, growth factors, ions, odorant and tastant molecules and even photons of light [8]. They are highly dynamic proteins that can adopt a range of conformations, some of which are sparsely populated in the ligand-free receptor. Binding of an agonist at the extracellular region of the TM domain of the GPCR induces a shift in the conformational equilibrium, pushing the receptor through a series of discrete conformational intermediates, ultimately leading to large rearrangements at the intracellular region that facilitate the interaction with intracellular effectors including heterotrimeric G proteins, arrestins, and G protein-coupled receptor kinases that lead to downstream signalling and regulation [9].

The past decade has seen an increase in structure determination of GPCRs in atomic detail, predominantly through the application of X-ray crystallography [10]. These studies have revealed the arrangement of the TM domain, location of ligand binding pockets, interaction patterns exhibited by agonists and inhibitors (antagonists and inverse agonists), and the structural rearrangements involved in conformational changes upon GPCR activation [11]. The GPCR structural coverage has now reached 192 structures of 44 different GPCRs, of which most belong to the Class A subfamily [12]. To date, most of these GPCR structures are in an inactive conformation, bound to an inhibitor, however more recently structures bound to agonists have been solved. These include intermediate conformations (e.g. beta-1 adrenergic receptor (B1AR) [13], beta-2 adrenergic receptor (B2AR) [14] and adenosine A2a receptor (AA2AR) [15,16]) that are solved without an intracellular effector and fully active receptors (e.g. bovine rhodopsin [17,18], B2AR [19–21], muscarinic acetylcholine receptor M2 (M2R) [22] and AA2AR [23]), solved with an agonist ligand and intracellular partner (either a G protein C-terminal fragment, heterotrimeric G protein, mini G alpha protein, G protein mimicking nanobody or an arrestin). Together these structures provide unprecedented insight into the structural and functional diversity of this protein family [24].

The wealth of structural information on Class A GPCRs (the GPCR superfamily targeted by the largest number of clinically used drugs [25]) is invaluable for structure-based drug discovery (SBDD) programs that complement traditional drug discovery efforts. These include using rational substitutions within ligand design that prioritise medicinal chemistry directions, and identification of new scaffolds or compounds using virtual screening (VS), which ranks libraries of small molecules based on the predicted interaction score between ligand and receptor binding pocket. VS has been extensively and successfully used on many soluble protein drug targets (e.g. enzymes) and more recently for GPCRs [26–31]. However, to fully harness VS for GPCR SBDD, greater structural coverage and diversity is required than has currently been experimentally derived, both in terms of the number of unique GPCRs and the variety of ligands that are bound to these receptors.

For GPCRs where structures are available, it is critical to increase the diversity of ligands bound in these structures to enhance their power in VS, as even small conformational differences are enough to distinguish agonist-bound from inhibitor-bound complexes. Indeed, principal component analysis (PCA) (see description in the Methods section) applied on B2AR and AA2AR X-ray structures that are used in this study identifies a separation between agonist-bound and inhibitor-bound binding pockets based on their conformation (S1A and S1B Fig). This has already been shown for rhodopsin X-ray structures [32]. Further, analysis of ligand-receptor interaction patterns with interaction fingerprint (IFP) clustering [33] (see description in the Methods section) reveals that agonist-bound and inhibitor-bound binding pockets have distinct fingerprints for individual receptors (S1C and S1D Fig). These small differences can influence a VS such that an inhibitor-bound structure will preferentially select for inhibitors in a VS and vice-versa for an agonist-bound structure [34]. While this information can be leveraged to bias VS of binding pockets towards the identification of ligands of a desired pharmacology, as shown in studies on adrenoceptors [35], it can also hinder the identification of novel ligands and ligands of a particular pharmacology in VS where limited structures are available. To attempt to overcome this, multiple GPCR binding pocket refinement methods have been applied to both X-ray crystal structures and GPCR homology models.

Molecular dynamics (MD) is a powerful computational tool that can be used for the refinement of a ligand-bound GPCR structure [36]. Classical MD has been used to understand GPCR activation mechanisms [37] and for GPCR model refinement [38,39]. Enhanced sampling methods, like accelerated MD, have also been used to explore receptor flexibility around a known ligand [40]. Two other methods that rely on MD are also gaining a lot of interest; (i) Markov state modeling, to identify ligand-induced binding pocket conformational changes [41] and (ii) free energy perturbations, to accurately recapitulate ligand binding affinities using X-ray structures [42] or a homology model [43] and the effect of mutations on agonist [44] and antagonist ligands [45]. MD however, remains a computationally expensive technique and efforts towards the development of computational methods that are more tractable for GPCR model refinement are being explored. One such set of methods, termed ligand-guided or ligand-steered modeling, relies on the optimization of residue conformations around a bound ligand to improve binding pocket VS performance using either manual [46] or automated protocols [47–49]. Another set of methods involves sampling of the whole receptor structure in the presence of a known ligand for the target receptor using protein sampling methods such as normal mode analysis [50–52] and Monte Carlo sampling [53]. Other methods focus on building de novo GPCR models, relying only on the protein sequence. These include threading [54,55] and helix packing [56–59] methods.

In this study, we present a novel computationally efficient ligand directed modeling (LDM) workflow that performs extensive sampling of a GPCR and ligand conformation to obtain a low energy minimum of the complex. The efficiency is achieved by running independent conformational searches simultaneously on multiple compute cores and leveraging computationally efficient protein geometry-based sampling [60,61] and ligand molecular docking [62] methods. These methods also facilitate overcoming energy barriers. Furthermore, the LDM is an iterative process that integrates a combination of scoring functions used to prioritize complexes for further sampling, thus focusing the conformational search in the relevant space. Importantly, this method requires only a single known ligand for the GPCR to undergo its refinement, which is a sought-after feature for SBDD programs where only few ligands may be known. We evaluate the performance of this LDM protocol across a variety of refinement tasks of increasing difficulty, and identify advantages and shortcomings of using this LDM method for GPCR binding pocket refinement. The LDM generated models were evaluated using retrospective VS where we analysed the recovery of known ligands over decoys, the selectivity of agonists over inhibitors (or vice-versa) and the enrichment of specific ligand chemotypes. We demonstrate broad utility of this LDM approach for improving VS performance, particularly when searching for ligands of a defined pharmacology (for example agonists over inhibitors).

## Methods

### LDM workflow

**Fig 1:**
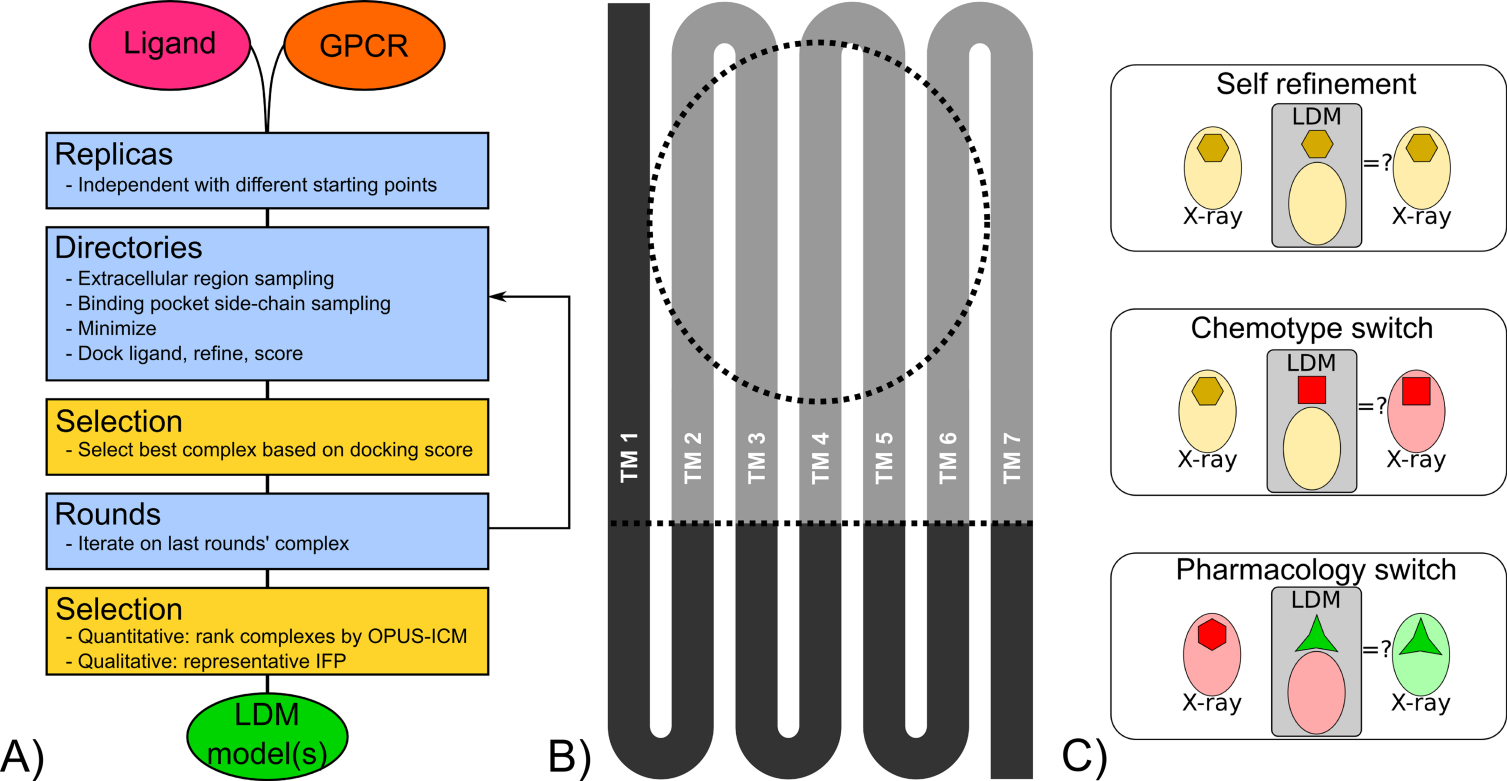
LDM workflow description. A) Schematic of the LDM workflow that takes a GPCR model and a ligand as input, and outputs LDM models. Sampling steps are represented in blue while selection steps are represented in yellow. B) GPCRs are separated in regions in the LDM workflow that have increasing degree of sampling. The TM1 and cytoplasmic region are kept static (dark grey), the rest of the GPCR is flexible (light grey). The binding pocket region defines the docking area and undergoes further sampling (dotted circle). C) The LDM was evaluated with a benchmark divided into three scenarios where an origin X-ray crystal structure binding pocket is refined by the LDM using the ligand found in a destination X-ray crystal structure, referred to here as origin and destination, respectively. In self refinement, the origin and destination are the same structure, in chemotype switch, the origin and destination are bound by ligands of the same pharmacology (agonist or inhibitor) but with different chemotypes and in pharmacology switch, origin and destination are bound by ligands of different pharmacology (agonist to inhibitor or vice-versa).

The LDM method performs a GPCR binding pocket refinement around a small molecule ligand to establish a low energy minimum of the GPCR/ligand complex. The method requires an initial GPCR structure and a known small molecule ligand for the target GPCR. The LDM is optimised for refinement using ligands that bind to the canonical orthosteric binding pocket of Class A GPCRs, formed by the top of third of the TM bundle, using parameters defined in S1 Table. The highly dynamic loops are deleted from the initial model, except for the distal portion of the extracellular loop 2 (ECL2). This portion consists of the segment of ECL2 downstream of the conserved ECL2 cysteine that forms a disulfide bridge with TM3. Although the ECL2 involvement in ligand recognition and kinetics has been extensively reviewed [63,64], ECL2 was shown not to be critical to VS outcome [65] and ECL2-distal specifically was shown to be involved in the orthosteric ligand final binding pose from many class A GPCR crystal structure analyses previously reviewed [5]. Ligand docking, which is a key component of the LDM workflow described below, does not apply any selective pressure on the conformation of other extracellular loops (ECLs) or intracellular loops (ICLs). The inclusion of ECLs and ICLs expands the conformational search space, thus for equal search time, reduces the conformational space explored within the more relevant canonical Class A GPCR binding pocket. Additionally, the highly flexible ECLs may occlude this binding pocket and prevent ligand docking on some generated conformations. Furthermore, two mutually exclusive regions are defined on the TM bundle, which are processed differently during the LDM workflow. The “extracellular region” corresponds to upper segments of TMs 2–7, while the “cytoplasmic region” corresponds to TM1 and the bottom of TMs 2–7 (Fig 1B). The cutoff point is a set of user defined residues that are located below the canonical orthosteric Class A binding pocket; these residues correspond to 1.48, 2.51, 3.38, 4.51, 5.50, 6.43, 7.45 defined using Ballesteros-Weinstein nomenclature [66]. This makes the extracellular region approximately 2/3 of the TM bundle and the rigid cytoplasmic region thus ensures that generated receptor structures retain their seven TM fold. The extracellular region, which contains the binding pocket, is also user defined before the start of the LDM but is updated during the workflow. The starting binding pocket was defined in this study by selecting residues within 1.5 Å of the binding pocket defined using the ICMPocketFinder tool. A box is created around these residues and used to calculate the docking grid.

The LDM workflow is provided as a set of scripts written in Bash designed to run on a computer cluster using either the SLURM or the PBS submission system (https://github.com/thomas-coudrat/ldm_scripts), with parameters that can be modified by the user. The parameters used in the following description (described in more detail in S1 Table) are the recommended parameters and those that were used in this study. The LDM workflow (Fig 1A) initiates several independent replicas where the input receptor structure is minimised with explicit waters using GROMACS [67] and undergoes minimal sampling with CONCOORD [60]. This ensures that each replica has a slightly different conformational starting point. In each replica, an iterative process is initiated and run over several rounds. Each round includes a series of sampling, selection and scoring steps with the first round processing the receptor alone and all subsequent rounds processing the receptor-ligand complex in the same manner. Both receptor and receptor-ligand complexes will be referred to as the system in the following description. During each round, a set of independent directories is created. In each of these, the system undergoes extracellular protein sampling with CONCOORD [60], followed by a selection of the resulting binding pockets that are within a cutoff distance from the starting system. The selected systems then undergo binding pocket side-chain sampling with tCONCOORD [61]. Pockets that conserved a fraction of binding pocket polar residues from the starting system are selected. Both these selection steps ensure that the resulting system does not deviate too greatly from the original and retains a suitable binding pocket. The LDM process therefore prioritizes incremental conformational changes. The system’s side-chains, from the cytoplasmic region of the system, are rebuilt using sccomp [68], the whole system is then minimised in explicit waters with GROMACS readying it for the docking phase. ICM flexible ligand docking is performed with the single ligand onto the pre-calculated receptor docking grid. An area of 2 Å around the ligand is refined and ICM is used to score the ligand-receptor complex. This key step in the LDM workflow selects for binding pockets where the known ligand binds with a high score, thus providing a selective pressure on the binding pocket and the extracellular region of the receptor. The scoring phase within each replica then compares all complexes generated in individual directories based on their ICM score, with the best scoring complex extracted and assigned an OPUS score, using OPUS_PSP [69], which provides information on the overall quality of the protein fold. All other complexes are discarded. The extracted complex is then used as the starting point for a subsequent round of the iterative LDM workflow. After all rounds are completed, the LDM outputs a maximum number of models equal to the number of rounds times the number of replicas. This is a selection from a much larger total number of receptor-ligand complexes generated over the course of the workflow. These LDM output models are sorted with the quantitative ICM and OPUS score, where both methods are normalised and used in equal weights (OPUS-ICM score). In summary, the LDM workflow relies on the selection steps and scoring that prioritise binding pocket integrity and known ligand complementarity, the scoring that prioritises proteins with a correct fold and the static treatment of the cytoplasmic region of the receptor enable the LDM workflow. Together, these parameters optimise for GPCR binding pocket conformations that are compatible with a known ligand while not requiring an explicit lipid membrane for their computation. A description of the required software, preparation and parameters to run the LDM is provided in S1 Text.

### LDM assessment and benchmarks

X-ray structures used for this study (with abbreviations for GPCRs and their ligands) are listed in S2 Table. These structures were selected based on the criteria that they were of human origin with binding pockets containing no or a limited number of mutations and unresolved side-chains. Benchmarking was designed so that the target result for each application of the method was available in the form of an X-ray structure. The LDM was applied on an origin X-ray structure refined using the ligand bound in a destination X-ray structure and the LDM results were compared to both the origin and destination X-ray crystal structures. All GPCRs that were considered for benchmarking also had libraries of known ligands and decoys available from the GPCR ligand library/GPCR decoy database (GLL/GDD) [70], described below.

Three different scenarios were assessed that evaluate different conformational distance between the origin and the destination X-ray structures representing varying levels of difficulty (Fig 1C). In the first scenario, various GPCRs were selected to perform self refinement of a ligand-bound X-ray structure, where a binding pocket is refined using its own bound ligand. The chemotype switch scenario evaluated the LDM at refining an agonist-bound (or inhibitor bound) binding pocket using an agonist (or inhibitor) of a different chemotype. The conformational rearrangement is more pronounced in this case relative to the self refinement. These experiments required the selection of X-ray structures where a single GPCR was bound by ligands of different chemotype. The final scenario assessed was pharmacology switch that refined an inhibitor-bound binding pocket using an agonist for LDM refinement (or vice-versa). This requires larger rearrangements of the binding pocket's protein backbone and represents the most difficult task. Pharmacology switch experiments were performed using examples of aminergic GPCRs (B2AR and M2R) that are available in inactive and fully active conformations. A nucleotide GPCR (AA2AR) with available agonist-bound and inhibitor-bound structures was also chosen to complete this benchmark.

In one pharmacology switch LDM experiment, the origin and destination X-ray structures contained different mutations (introduced for stabilisation and crystallisation). One of the mutations was within 4 Å of the bound ligand in the destination X-ray structure. For this LDM experiment, four mutations were introduced in the origin X-ray structure 3EML [71] to match the sequence of the destination X-ray structure 2YDV [15] (S2 Table). In all other cases, gene sequences were either identical between origin and destination X-ray structure, or mutations occurred far from the binding pocket.

### Protein-ligand conformation analysis

As shown previously, the success of a GPCR binding pocket performance in VS is linked with the ICM interactive score and ligand/receptor interaction pattern [72]. The LDM workflow leverages the ICM interactive score during its iterative process, and combines this with the OPUS_PSP score for final ranking of LDM output models. To gain further insight into the outcome of the LDM workflow, LDM models were analysed using qualitative methods. For each LDM experiment described in this study, all LDM output models were superimposed onto the destination X-ray structure and the top 25 LDM models (models LDM 000 to LDM 024) along with the origin and destination X-ray structures were further analysed. Ligand/receptor interaction patterns were analysed using interaction fingerprints (IFPs) [73]. An IFP is a vector of booleans (True or False) that encode for the interaction type between each of the ordered list of residues lining the binding pocket and the bound ligand. Binding pocket conformational changes were also analysed using PCA, a dimensionality reduction algorithm. These analyses were performed using a set of Python [74] scripts developed in the laboratory: toolbox_pdb (https://github.com/thomas-coudrat/toolbx_pdb) [72]. These scripts use the open source libraries Matplotlib [75], Numpy [76], SciPy [77] and the PCA implementation uses scikit-learn [78] while the IFP implementation uses the OpenEye OEChem toolkit version 2014.10.2 [79].

#### Interaction fingerprints

IFPs were calculated for the top 25 LDM results and both origin and destination X-ray structures. IFPs were used as described by Marcou and Rognan [73] that include the presence or absence of hydrophobic, weak hydrogen bond (donor and acceptor), hydrogen bond (donor and acceptor), ionic (positive or negative) and aromatic interactions of each binding pocket residue with the ligand. Graphical outputs were generated for inspection of specific differences in interaction patterns, and IFPs were also clustered using the Jaccard distance with the result visualised as a dendrogram. The Jaccard distance is used as a measure of the dissimilarity between two boolean vectors of length *n* (IFPs), calculated using Eq 1 where the Jaccard index of co-occurence C_*ij*_ is the number of occurrences of *u*[*k*] = *i* and *v*[*k*] = *j* for *k*<*n*. In these dendrograms, a distance of 0 corresponds to identical IFPs. A cutoff Jaccard distance defines the number of clusters at that distance. In this study, a distance of 0.6 or higher was routinely assigned to signify two sufficiently different IFPs. This cutoff was lowered to a less stringent value in a few cases, when the number of different clusters generated was large at 0.6. Grouping the LDM models and X-ray structures using IFP clustering aided prioritisation of LDM binding pockets for further assessment. Each LDM model was scored, ranked and named based on the OPUS-ICM metric, thus the highest scoring LDM model in each IFP cluster could be identified by its name and was thereafter defined as the representative for that cluster. These representative LDM models were assessed in VS and their ligand/receptor conformations were further analysed and compared to the origin and destination X-ray structures.

$$d_{J}=\frac{C_{TF}+C_{FT}}{C_{TT}+C_{TF}+C_{FT}}$$

#### Principal component analysis

The binding pocket conformation of the top 25 LDM models and X-ray structures were examined by comparing the positions of carbon alphas of the binding pocket residues using PCA that extracts the main variance of a dataset revealing its internal structure [80]. Principal components (PCs) are ordered based on their percentage of cumulative variance explained from the original dataset. The first two PCs were plotted onto a PCA score that provides information on the relationship between the binding pockets analysed, but also the importance of these relationships relative to the total amount of variance in the original dataset. The percentage of variation explained by each PC is shown in parenthesis (e.g. PC1 explains 50% of the variance in the data). Finally, a colour map was generated to display the IFP Jaccard distance between each of the complexes and the destination X-ray structure on the PCA plot.

### Virtual screening

Retrospective VS performance was performed on the representative LDM binding pockets from each IFP cluster and compared to the VS performance of the origin and destination X-ray structures. The GLL/GDD small molecule libraries [70], which match known GPCR agonists and inhibitors with a respective sets of decoys of similar physical properties, was used for VS. The ligand libraries were optimised prior to use by deleting duplicates, adding X-ray co-crystal ligands from this study. The ligand library was then modified to a racemic library format, as described previously [72]. The activity profile of many ligands is experimentally determined on a racemic mix while only one enantiomer is active, thus considering all enantiomers in SBDD ensures the right active ligand is assessed. Finally, the ligand charge was set per the ICM pKa model and no additional tautomers were generated.

When screening the libraries, each ligand docking was repeated three times, and the best of the three scores was attributed to the ligand for ranking. Three metrics were established for evaluating VS performance [72]: recovery, selectivity and chemotype enrichment. Recovery and selectivity were visualised using receiver operating characteristic (ROC) curves that compare the recovery rate of known ligands against decoys (recovery) and inhibitors against agonists, or vice-versa (selectivity). Normalised square-root area under the curve (NSQ_AUC) [81] values were calculated for all ROC curves. In addition, the behaviour of all binding pockets with regards to specific chemotype recovery was also analysed. Dendrograms were developed for all known ligand chemotypes for GPCRs used in this study (S2 Fig). Enrichment factors (EFs) at 1, 5 and 10% of the total library were plotted for representative chemotype clusters when using a 0.5 pharmacophore cutoff. Representative clusters were selected on the following criteria; (i) if they contained a ligand that was present in an X-ray structure used in this study and (ii) if they contained a large number of the known ligands, thus offering a good coverage of the chemical diversity of the known ligand library (S2 Fig). The setup, management and analysis of VS experiments, including the plotting of ROC curves and EF bargraphs was performed using the open-source and available Python scripts toolbox_vs developed in the laboratory (https://github.com/thomas-coudrat/toolbx_vs) [72]. This package uses the following libraries: Matplotlib [75], Numpy [76] and scikit-learn [78].

## Results

### The influence of loops on X-ray structure VS performance

**Fig 2:**
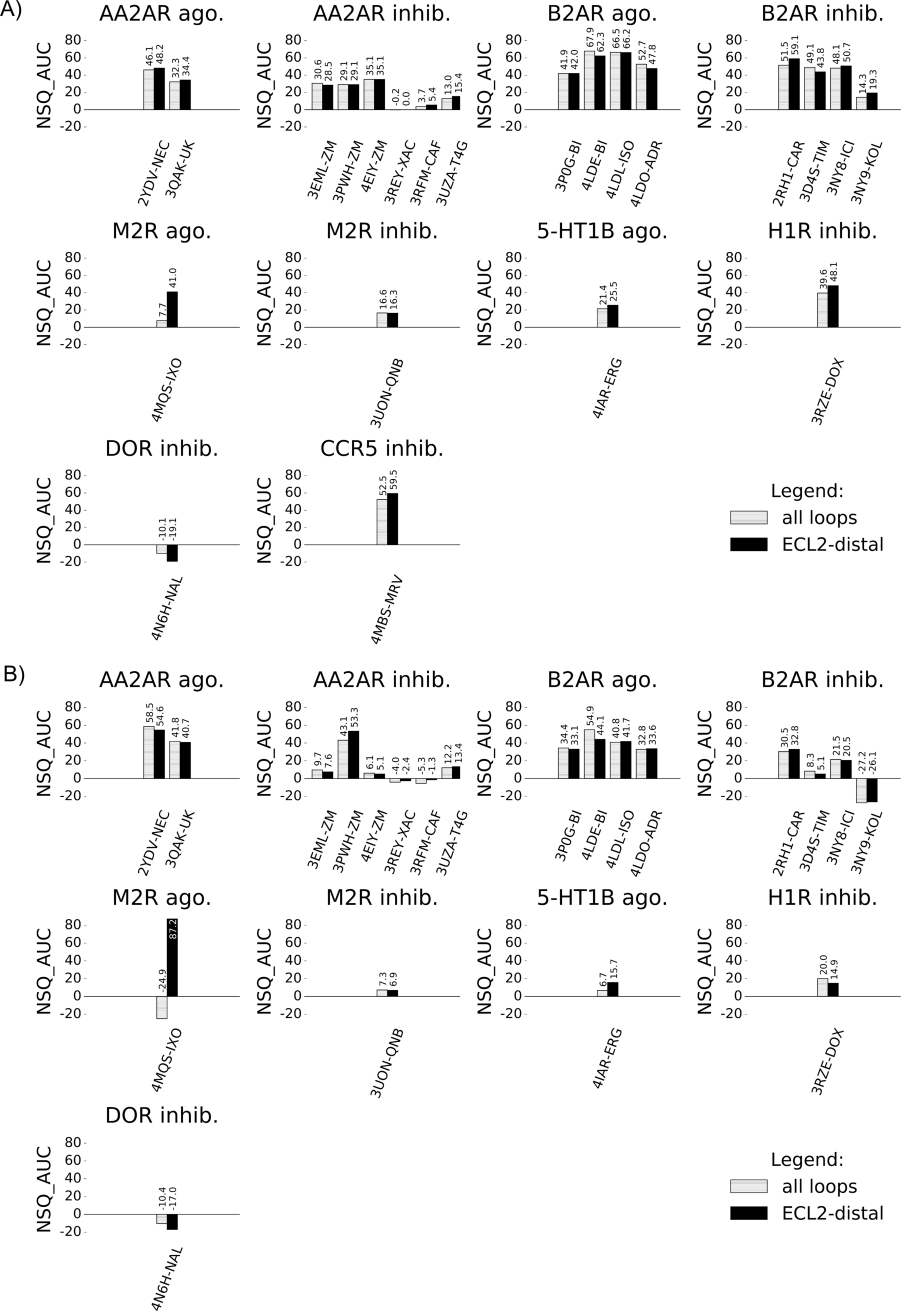
Comparison of VS performance for X-ray structures with all loops vs. ECL2-distal loop only. VS performance in NSQ_AUC values of X-ray structures measuring A) recovery of known ligands against decoys and B) selectivity of agonists vs. inhibitors (or vice-versa). X-ray structures with all loops are represented in dashed bars and X-ray structures with ECL2-distal only are represented in black bars.

The influence of the loops on VS performance was assessed for all X-ray structures used in this study. All X-ray structures where processed by ICM by adding hydrogens and missing side-chains and flipping asparagine, glutamine and histidine side-chains to improve molecular interactions. No further refinement or energy minimisation was performed on these X-ray structures. Two sets of binding pockets were generated; in the first set all X-ray structure ECLs were retained and in the second set all ECLs except for the distal part of ECL2 (ECL2-distal) were removed. The binding pocket pairs were compared using VS recovery and selectivity and plotted as ROC curves (S3 and S4 Figs) from which NSQ_AUC values were calculated (Fig 2). Overall, removing loops while leaving the ECL2-distal had little influence on VS recovery (Fig 2A) or selectivity (Fig 2B). However, a notable difference was identified with the M2R agonist-bound structure 4MQS-iperoxo (IXO), where the removal of loops with the exception of the ECL2-distal region resulted in a large improvement in VS performance, in both recovery and selectivity.

### Self refinement

In this scenario, the ligand used for LDM refinement was the ligand found in the origin X-ray structure. The LDM models were thus compared to a single X-ray structure. The analysis of the results for this scenario was separated into two groups. The first group is represented by experiments where the LDM models showed improved or similar VS performance in overall recovery and/or selectivity relative to the X-ray structure, and also with a ligand pose similar to that of the X-ray structure. The second group consists of experiments where LDM models had lower overall VS performance, even if their binding pose was similar to that of the X-ray structure. Despite this, in all self refinement experiments, the LDM workflow performed its task of refinement around the ligand used, either “narrowing” the chemotypes that were enriched in the LDM models so that only the chemotypes similar to the refinement ligand were identified or displaying improved enrichment for the refinement ligand chemotype relative to the origin X-ray structure.

#### Group A

**Fig 3:**
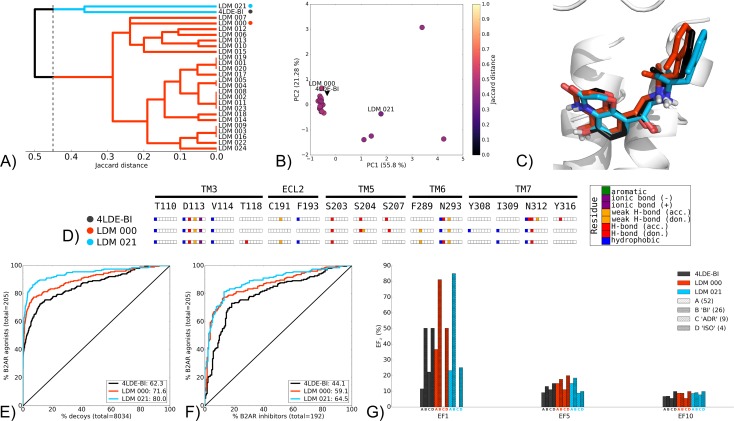
Self refinement LDM experiment on the B2AR 4LDE-BI, using BI as a refinement ligand. A) Dendrogram of the top 25 LDM models and the X-ray structures, a cut-off line identifies different LDM clusters and their representative LDM models are designated by a coloured dot. Representative LDM models are the highest scoring within the cluster based on the OPUS-ICM metric. B) PCA comparison of binding pocket conformation between the top 25 LDM models and the X-ray structures. LDM models are coloured based on their IFP Jaccard distance with the destination X-ray structure, 4LDE-BI. C) Binding poses of the representative LDM models and the destination X-ray structure with the X-ray ligand in black, LDM000 in red and LDM021 in blue. D) IFP of the representative LDM models and the X-ray structure. Interaction type is described for each residue of the binding pocket: hydrophobic interaction, hydrogen bond (H-bond) donor and acceptor, weak hydrogen bond (weak H-bond) donor and acceptor, ionic bond positive (+) and negative (-) and aromatic interaction. E, F, G) VS performance with the X-ray structure in black, LDM000 in red and LDM021 in blue. E) ROC curves visualising the recovery of B2AR agonists vs. decoys. F) ROC curves highlighting the selectivity of B2AR agonists over B2AR inhibitors. For E and F, the relative rank of the LDM refinement ligand is identified with a vertical dashed line. This vertical line is not visible as it overlays the axis indicating the refinement ligand is very highly ranked in all 3 cases. The ROC curve figure insets show NSQ_AUC values for each binding pocket. G) bar chart to visualise the EF for representative B2AR agonist chemotypes at EF1, EF5 and EF10. Chemotypes A, B ‘BI-like’, C ‘ADR-like’ and D ‘ISO-like’ represent only a subset of B2AR agonist ligands (S2C Fig). The EF bar chart inset shows the number of ligands for each of these chemotype clusters between parenthesis. X-ray structure chemotype EF shown in black bars, with the LDM models coloured based on their relative clusters identified in A.

The first group is described in detail using the LDM experiment B2AR 4LDE-BI-167,107 (BI) (Fig 3). Seven other LDM experiments were performed that displayed a similar pattern, including B2AR 3P0G-BI (S5 Fig), AA2AR 4EIY-ZM-241,385 (ZM) (S6 Fig), 3PWH-ZM (S7 Fig), M2R 3UON-3-quinuclidinyl-benzilate (QNB) (S8 Fig), M2R 4MQS-IXO (S9 Fig), histamine H1 receptor (H1R) 3RZE-doxepin (DOX) (S10 Fig) and 5-hydroxytryptamine receptor 1B (5-HT1B) 4IAR-ergotamine (ERG) (S11 Fig). The B2AR BI-refined LDM models converged towards a similar binding pose to that of the X-ray structure, as the IFP of the top 25 LDM models are within 0.5 in Jaccard distance (Fig 3A) and form two clusters at a 0.45 cut-off, with representatives LDM 000 and LDM 021 for each cluster. Although LDM 021 was the closest to the X-ray structure in terms of interaction pattern, its binding pocket conformation was more distant to the X-ray structure than LDM 000 (Fig 3A and 3B). The binding poses overlaid very closely (Fig 3C), and both LDM models displayed the same polar and ionic interaction pattern as the X-ray structure at D113, S203 and N312 (Fig 3D). The two LDM models differed in their polar contacts, with LDM 000 displaying the same interaction with S204 and S207 as the X-ray structure, whereas LDM 021 had the same interaction pattern with N293 as the X-ray structure and an additional polar contact with T118.

Both LDM models outperformed the X-ray structure in overall VS recovery and selectivity (Fig 3E and 3F). In both cases, LDM 021 was the best performer, with NSQ_AUC values of 80.0 and 64.5 in recovery and selectivity, respectively. The chemotype enrichment analysis revealed an improved enrichment for the chemotype of the refinement ligand BI, for both LDM models compared to the X-ray structure at EF1 (Fig 3G). Interestingly, the highly populated chemotype cluster A was also enriched in both LDM models compared to the X-ray structure, while the under-populated chemotype cluster C was enriched to a lesser extent compared to the X-ray structure at EF1.

Key polar, ionic and aromatic interactions were recovered by LDM models in all seven experiments from this group, as illustrated for B2AR 4LDE-BI. However, notable ionic contacts were absent from LDM models in three experiments from this group (S8–S10 Figs). Despite this, as was the case for B2AR 4LDE-BI, LDM models showed improved enrichment for the refinement ligand chemotype and for other chemotypes in two other experiments (S5 and S10 Figs). However, in the five other experiments from this subgroup overall VS was similar to the X-ray structure, but with improvement and/or narrowed enrichment towards the refinement ligand chemotype (S6–S8 and S11 Figs).

#### Group B

**Fig 4:**
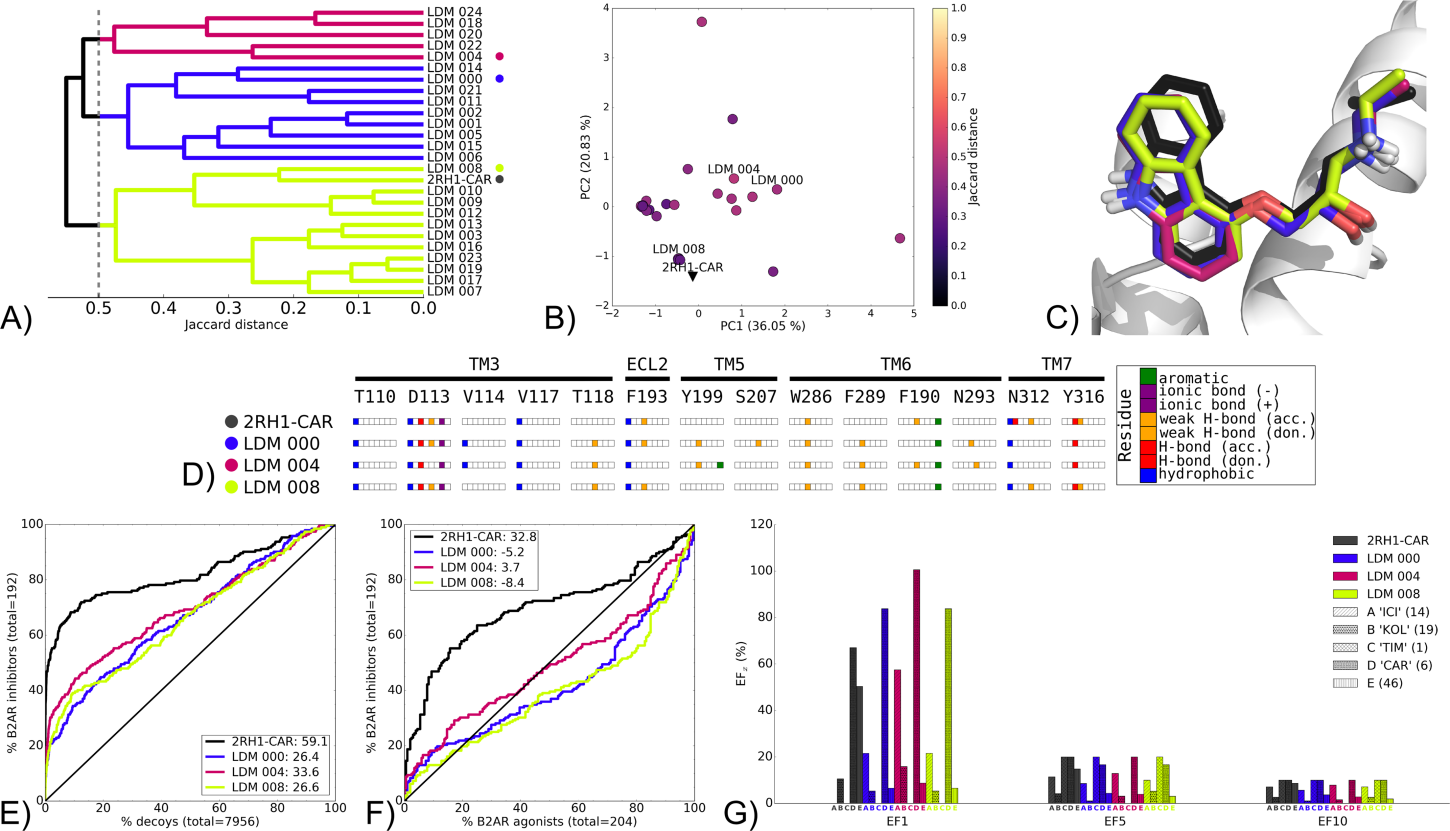
Self refinement LDM on the B2AR 2RH1-CAR, using CAR as the refinement ligand. A) Dendrogram of the top 25 LDM models and the X-ray structure, a cutoff line identifies different LDM clusters. Representative LDM models are the highest scoring within the cluster based on the OPUS-ICM metric and are designated by a coloured dot. B) Comparison of binding pocket conformation between the top 25 LDM models and the X-ray structure. LDM models are colored based on their IFP Jaccard distance with the destination X-ray structure, 2RH1-CAR. C) Binding poses of the representative LDM models and the destination X-ray structure with the X-ray ligand in black and the LDM models coloured based on their representative clusters defined in A. D) IFP of the representative LDM models and the X-ray structure. Interaction type is described for each residue of the binding pocket: hydrophobic interaction, hydrogen bond (H-bond) donor and acceptor, weak hydrogen bond (weak H-bond) donor and acceptor, ionic bond positive (+) and negative (-) and aromatic interaction. VS performance is described with ROC curves to visualise E) the recovery of B2AR inhibitors vs. decoys and F) the selectivity of B2AR inhibitors over B2AR agonists. X-ray structures are in black, with the LDM models coloured based on their relative clusters identified in A. The relative rank of the LDM refinement ligand is identified with a vertical dashed line. In all cases, this vertical line is masked by the axis indicating that the refinement ligand was very highly ranked. The ROC curve figure inset shows NSQ_AUC values for each binding pocket. G) bar chart is used to visualise the EF for representative B2AR inhibitor chemotypes at EF1, EF5 and EF10. Chemotypes A ‘ICI-like’, B ‘KOL-like’, C ‘TIM-like’, D ‘CAR-like’ and E represent only a subset of B2AR inhibitor ligands (S2D Fig). The EF bar chart inset shows the number of ligands for each chemotype cluster between parenthesis. X-ray structure chemotype EF shown in black bars, with the LDM models coloured based on their relative clusters identified in A.

The second group of self refinement LDM experiments is illustrated by B2AR 2RH1 refined by the ligand carazolol (CAR) (Fig 4). The three representative LDM models selected from IFP clustering were very similar to the X-ray structure at 0.5 cutoff (Fig 4A, and 4C). Interestingly, LDM 008 was closest to the X-ray structure in both IFP (Fig 4A) and in binding pocket conformation (Fig 4B). The three LDM models had similar IFPs with LDM 004 uniquely displaying an aromatic interaction with Y199 (Fig 4D). However, none of the LDM models featured the polar interaction with N312 observed in the X-ray structure 2RH1-CAR.

All LDM models had a similar VS performance pattern, with LDM 004 slightly outperforming LDM 000 and LDM 008 in recovery (Fig 4E) and selectivity (Fig 4E). However, the X-ray structure (2RH1) binding pocket outperformed all LDM models in both overall recovery and selectivity (Fig 4E and 4F). Nonetheless, in all LDM models, the binding pockets were more selective for the CAR chemotype, as identified by their higher EF1 values. In addition, the best VS performer amongst LDM models, LDM 004, also had an improved chemotype enrichment for ICI-like and KOL-like B2AR inhibitor chemotypes compared to the 2RH1-CAR (Fig 4G). In contrast, chemotype cluster E that represents a much larger number of known ligands was poorly enriched in all the LDM models relative to the X-ray structure.

The results of B2AR 2RH1-CAR were interesting as LDM models converged towards conformations that were very similar to the X-ray structure, yet overall VS performance was reduced for LDM models relative to the X-ray structure. However, chemotype enrichment for those related to the refinement ligand was higher. The same pattern was seen with AA2AR 2YDV-NEC (S12 Fig). While C-C chemokine receptor type 5 (CCR5) 4MBS-maraviroc (MRV) (S13 Fig) and δ-opioid receptor (DOR) 4N6H-naltrindole (NAL) (S14 Fig), did not converge to a unique binding pose, all experiments from this group had improved recovery for the refinement ligand chemotype (while also excluding other chemotypes, hence narrowing towards the refinement ligand chemotype) compared to their X-ray structure (Fig 4G and S12G, S13F and S14G Figs). In addition, similar to B2AR 2RH1-CAR, none of these LDM models outperformed the X-ray structures in overall VS performance.

### Chemotype switch

We next evaluated the capacity of the LDM workflow to refine a GPCR binding pocket using a ligand of the same pharmacology (agonist or inhibitor) to that of the starting structure but belonging to a distinct ligand chemotype. This represents a larger conformational change relative to the self refinement. As with the self refinement studies, the LDM outcomes are presented in two separate groups with the first group describing LDM experiments where LDM models exhibited a better VS performance than the origin X-ray structures, with a similar binding pose to that of the destination X-ray structure. The second group comprised LDM experiments where LDM models improved VS performance over the origin X-ray structure, but had binding poses that differed from that of the destination X-ray structure.

#### Group A

**Fig 5:**
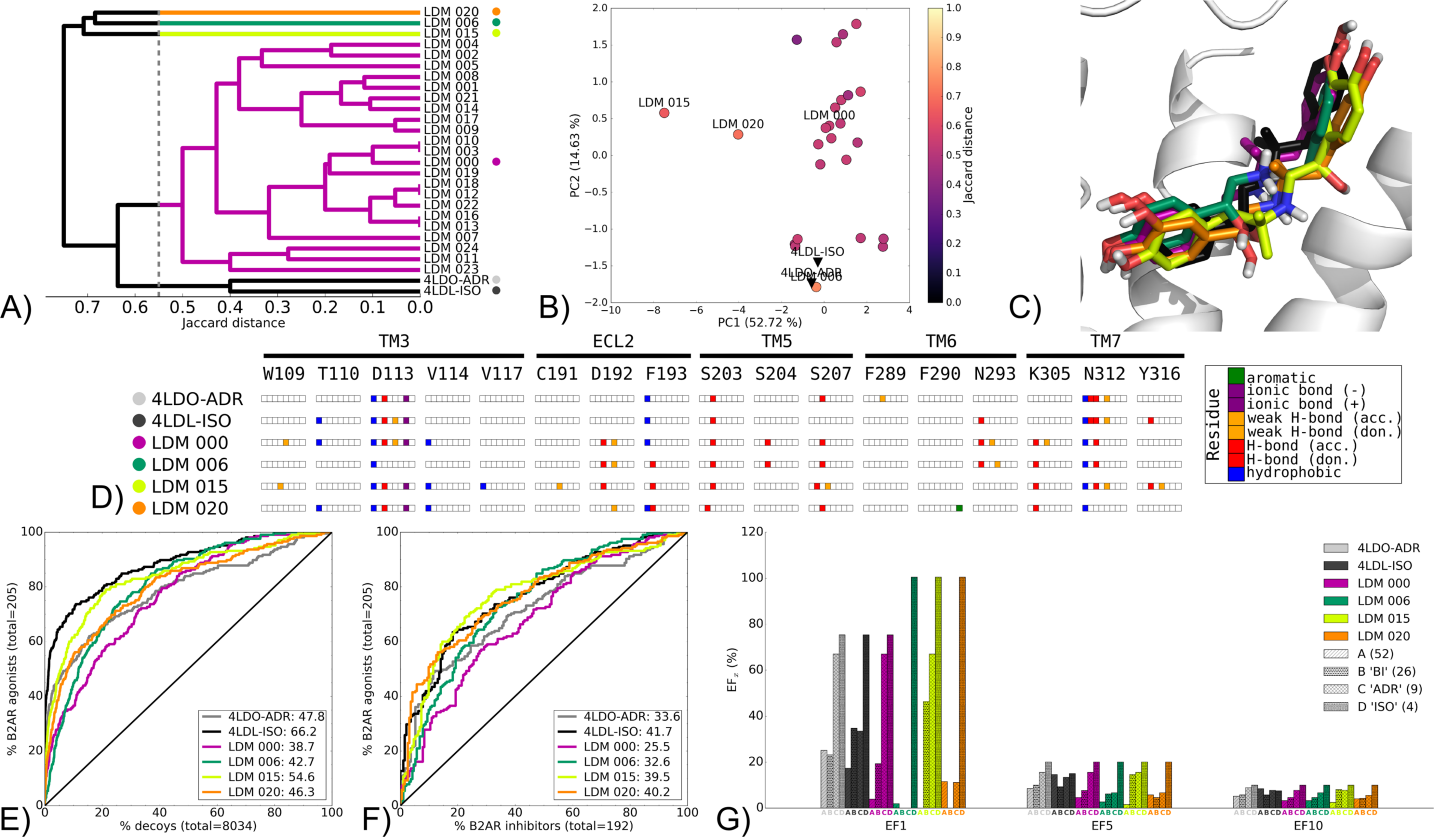
Chemotype switch LDM experiment on the B2AR from 4LDO-ADR to 4LDL-ISO, using ISO as a refinement ligand. A) Dendrogram of the top 25 LDM models and X-ray structures, a cutoff line identifies different LDM clusters and their representative LDM models are designated by a colored dot. Representative LDM models are the highest scoring within the cluster based on the OPUS-ICM metric. B) Comparison of binding pocket conformation between the top 25 LDM models and X-ray structures. LDM models are colored based on their IFP Jaccard distance with the destination X-ray structure, 4LDL-ISO. C) Binding poses of the representative LDM models and the destination X-ray structure with the X-ray ligand in black and the LDM models coloured based on their representative clusters defined in A. D) IFP of the representative LDM models and the X-ray structures. Interaction type is described for each residue of the binding pocket: hydrophobic interaction, hydrogen bond (H-bond) donor and acceptor, weak hydrogen bond (weak H-bond) donor and acceptor, ionic bond positive (+) and negative (-) and aromatic interaction. VS performance is described with ROC curves to visualise E) the recovery of B2AR agonists vs. decoys and F) the selectivity of B2AR agonists over B2AR inhibitors. X-ray structures are in black, with the LDM models coloured based on their relative clusters identified in A. The relative rank of the LDM refinement ligand is identified with a vertical dashed line. In all cases, this vertical line is masked by the axis indicating that the refinement ligand was very highly ranked. The ROC curve figure inset shows NSQ_AUC values for each binding pocket. G) bar chart is used to visualise the EF for representative B2AR agonist chemotypes at EF1, EF5 and EF10. Chemotypes A, B ‘BI-like’, C ‘ADR-like’ and D ‘ISO-like’ represent only a subset of B2AR agonist ligands (S2C Fig). The EF bar chart inset shows the number of ligands for each chemotype cluster between parenthesis.

This group is illustrated by the LDM experiment on the B2AR 4LDO X-ray structure bound by the agonist adrenaline (ADR), refined using the agonist hydroxybenzyl isoproterenol (ISO) found in the X-ray structure 4LDL (Fig 5). The group also includes the B2AR 3NY9 bound by the inhibitor described by Kolb et al. [82] (KOL), using CAR found in the X-ray structure 2RH1 (S15 Fig), AA2AR from 3QAK bound by UK-432,097 (UK) refined by NECA (NEC) found in the X-ray structure 2YDV (S16 Fig) and B2AR from 3D4S bound by timolol (TIM) refined by ICI-118,551 (ICI) found in the X-ray structure 3NY8 (S17 Fig).

The IFP clustering and visualization for B2AR 4LDO-ADR and 4LDL-ISO revealed the origin and destination X-ray structures shared a similar ligand/receptor interaction pattern (Fig 5A and 5D). Indeed, both complexes were characterised by polar contacts with D113, S203 and N312 and an ionic interaction with D113. Their conformational similarity was also illustrated by the relatively small distance in their PCA scores performed on binding pocket carbons alphas (Fig 5B). In this LDM experiment, the top 25 models mostly converged towards a large cluster represented by LDM 000, and three single leaf clusters containing LDM 006, LDM 015 and LDM 020 (Fig 5A). The binding poses for all these LDM models were close to that of the destination X-ray structure, with the exception of LDM 015 that was flipped around the anchor point formed by an ionic interaction between ISO’s tertiary amine and D113 (Fig 5C and 5D). While several LDM models contained interactions present in one or both of the X-ray structures, they also contain several additional polar interactions with ECL2 (D192 and F193), TM5 (S204) and TM6 (K305).

Both the origin and destination X-ray structures performed well in recovery and agonist selectivity (Fig 5E and 5F), and they both obtained high EF1 values for chemotypes of their respective crystal ligands (Fig 5G). Overall the LDM models also performed well in both VS performance evaluations. Specifically, when compared to the origin X-ray structure, LDM 015 was superior in both recovery and selectivity while LDM 020 was also good at recovery and superior in selectivity. Interestingly, LDM 015 had a flipped binding pose while maintaining a similar ligand/receptor interaction pattern to the destination X-ray and other LDM models. In terms of chemotype recovery, all LDM models outperformed the destination X-ray structure 4LDL-ISO at EF1 for the D “ISO-like” chemotype, except for LDM 000 that had the same EF1 score. The success of LDM 015 in overall VS performance can be linked to its superior EF1 score, relative to all other LDM models, for chemotype B “BI-like”, which is widely represented amongst B2AR agonists (26 ligands at 0.5 cutoff) (S2C Fig).

The other LDM experiments from this group exhibited a range of IFP distances between origin and destination X-ray structure (with Jacaard distances of ~0.6 for B2AR 3NY9-KOL to 2RH1-CAR, ~0.7 for AA2AR 3QAK-UK to 2YDV-NEC and ~0.3 for B2AR 3D4S-TIM to 3NY8-ICI, compared to ~0.4 for B2AR 4LDO-ADR to 4LDL-ISO (S15–S17A Figs and Fig 5A)). Two LDM experiments showed convergence towards the destination X-ray structure IFP (S15A and S16A Figs), while a third one (B2AR 3D4S-TIM to 3NY8-ICI) had its best performing LDM model appear in a small IFP cluster that was closest to the destination X-ray structure’s interaction pattern (S17A Fig). This was similar to the B2AR 4LDO-ADR 4LDL-ISO example described above. For the B2AR 3D4S-TIM to 3NY8-ICI LDM experiment, we decided to evaluate all LDM models from this minor IFP cluster (S17A Fig) for VS performance (S18 Fig). The best VS performer in this cluster was the lower ranked LDM 020. Interestingly, this model did not exhibit the ionic interaction between the ligand and D113 that was found in all the X-ray structures (S18D Fig).

In the three cases outlined above, the analysis contained LDM models that outperformed their respective origin X-ray structures in VS recovery and selectivity (S15–S17E and S15–S17F Figs). In addition, in all these examples all LDM models improved enrichment of the LDM refinement ligand and its chemotype compared to the origin X-ray structure (S15–S17G Figs). LDM models from the B2AR 3NY9-KOL to 2RH1-CAR experiment even outperformed the destination X-ray structure 2RH1-CAR in CAR-like chemotype enrichment, and also displayed superior enrichment for chemotypes ICI-like and KOL-like compared to both origin and destination X-ray structures (S15G Fig).

#### Group B

**Fig 6:**
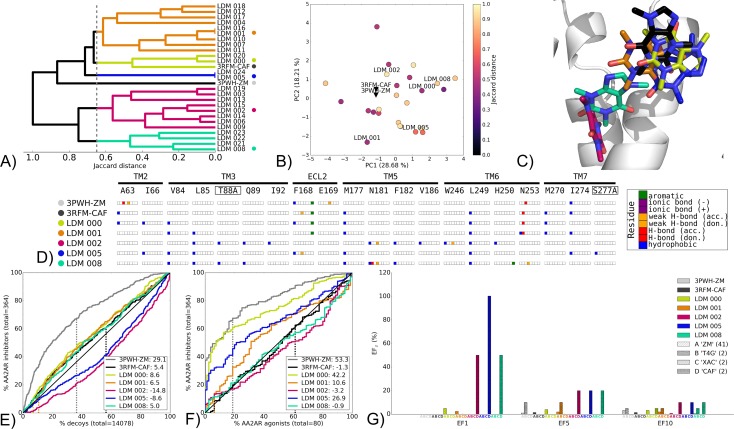
Chemotype switch LDM experiment on the AA2AR from 3PWH-ZM to 3RFM-CAF, using CAF as a refinement ligand. A) Dendrogram of the top 25 LDM models and X-ray structures, a cutoff line identifies different LDM clusters and their representative LDM models are designated by a colored dot. Representative LDM models are the highest scoring within the cluster based on the OPUS-ICM metric. B) Comparison of binding pocket conformation between the top 25 LDM models and X-ray structures. LDM models are colored based on their IFP Jaccard distance with the destination X-ray structure, 3RFM-CAF. C) Binding poses of the representative LDM models and the destination X-ray structure with the X-ray ligand in black and the LDM models coloured based on their representative clusters defined in A. D) IFP of the representative LDM models and the X-ray structures. Interaction type is described for each residue of the binding pocket: hydrophobic interaction, hydrogen bond (H-bond) donor and acceptor, weak hydrogen bond (weak H-bond) donor and acceptor, ionic bond positive (+) and negative (-) and aromatic interaction. VS performance is described with ROC curves to visualise E) the recovery of AA2AR inhibitors vs. decoys and F) the selectivity of AA2AR inhibitors over AA2AR agonists. X-ray structures are in black, with the LDM models coloured based on their relative clusters identified in A. The relative rank of the LDM refinement ligand is identified with a vertical dashed line. This vertical line for some models may be masked by others if the ligand is very highly ranked. The ROC curve figure inset shows NSQ_AUC values for each binding pocket. G) bar chart is used to visualise the EF for representative AA2AR inhibitor chemotypes at EF1, EF5 and EF10. Chemotypes A ‘ZM-like’, B ‘T4G-like’, C ‘XAC-like’ and D ‘CAF-like’ represent only a subset of AA2AR inhibitors ligands (S2B Fig). The EF bar chart inset shows the number of ligands for each chemotype cluster between parenthesis. Origin and destination X-ray structure chemotype EF shown in grey and black bars, respectively, with the LDM models coloured based on their relative clusters identified in A.

The second group of observed chemotype switch experiments is illustrated using the LDM experiment on AA2AR 3PWH-ZM using caffeine (CAF) for refinement with comparison of these LDM models to 3RFM-CAF (Fig 6). This group also contained the LDM experiment on AA2AR 3UZA-1,2,4-triazine 4g (T4G) using the xanthine amine congener (XAC) for refinement, with LDM models compared to 3REY-XAC (S19 Fig). In these studies, a selected LDM model improved VS performance over the origin X-ray structure, however with a ligand/receptor interaction pattern that was different to that of the destination X-ray structure.

For the featured experiment of AA2AR 3PWH-ZM to 3RFM-CAF, the origin and destination X-ray structures displayed markedly different ligand/receptor interaction patterns at ~0.9 Jaccard distance (Fig 6A), however their binding pocket conformations did not differ greatly (Fig 6B and S1A and S1B Fig). The LDM model IFPs displayed two distinct positions inside the binding pocket. A shallow binding pose that was shared by the X-ray structure 3RFM-CAF, and a deeper binding pose (Fig 6A and 6C). The group of shallow LDM models included representative models LDM 000, LDM 001 and LDM 005. The former two LDM models shared an aromatic contact with F168 and a polar interaction with N253 (Fig 6D) and these interactions were also present in both origin and destination X-ray structures.

Interestingly, the origin X-ray structure in this case outperformed the destination X-ray structure in VS recovery and selectivity, even for recovery of the destination ligand (Fig 6E, and 6F). Compared to the destination X-ray structure, several LDM models, including LDM 000 and LDM 001, had a similar recovery and all three shallow binding pose LDM models had an improved overall inhibitor versus agonist selectivity relative to the destination structure (Fig 6E and 6F). In terms of CAF chemotype enrichment at EF1, the LDM models that showed a deeper binding pose outperformed the ones with a shallower pose (Fig 6G), despite a distinct ligand pose to the destination structure. All models improved the relative ranking of CAF compared to both X-ray structures.

This group of chemotype switch LDM experiments was also represented by the LDM experiment AA2AR 3UZA-T4G to 3REY-XAC (S19 Fig). Relative to AA2AR 3PWH-ZM 3RFM-CAF described above, AA2AR 3UZA-T4G and 3REY-XAC origin and destination X-ray structures are more closely clustered in IFP (Fig 6A and S19A Fig). Another notable difference in this experiment relative to the previous was the refinement ligand size. CAF was the smallest ligand used in this entire study and XAC is a large ligand with several rotational degrees of freedom. Interestingly, as seen in the above example the origin X-ray structure outperformed the destination X-ray structure in VS recovery and selectivity (Fig 6E and 6F and S19E and S19F Fig). However, similar to the AA2AR 3PWH-ZM 3RFM-CAF experiment, LDM models in AA2AR 3UZA-T4G to 3REY-XAC LDM models outperformed the destination X-ray structure in VS recovery, selectivity and outperformed both the origin and destination structures in enrichment of the refinement ligand chemotype, XAC chemotype EF1 (S19E–S19G Fig).

### Pharmacology switch

**Fig 7:**
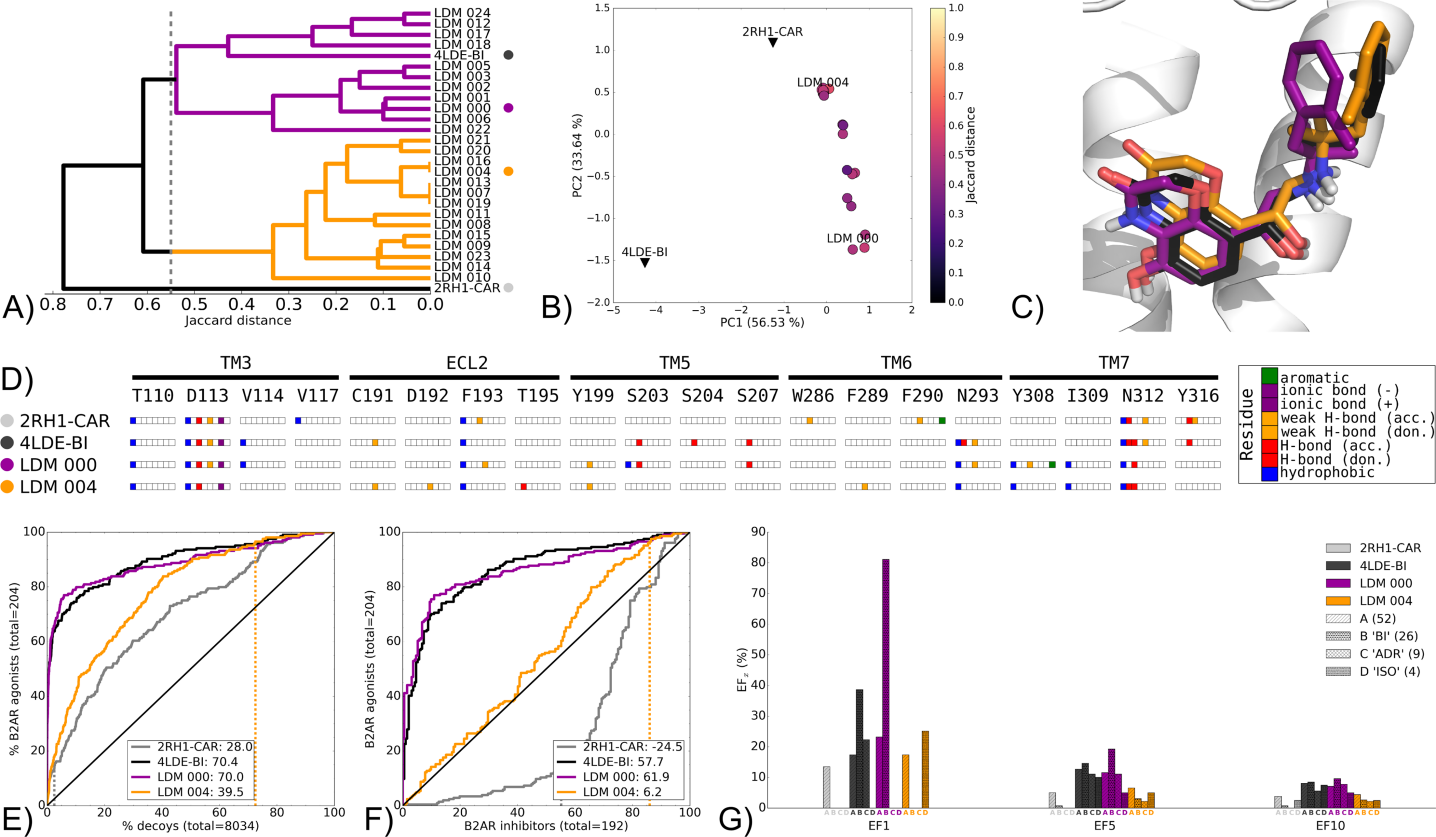
Pharmacology switch LDM experiment on the B2AR from 2RH1-CAR to 4LDE-BI, using BI as a refinement ligand. A) Dendrogram of the top 25 LDM models and X-ray structure(s), a cutoff line identifies different LDM clusters and their representative LDM models are designated by a colored dot. Representative LDM models are the highest scoring within the cluster based on the OPUS-ICM metric. B) Comparison of binding pocket conformation between the top 25 LDM models and X-ray structures. LDM models are colored based on their IFP Jaccard distance with the destination X-ray structure, 4LDE-BI. C) Binding poses of the representative LDM models and the destination X-ray structure with the X-ray ligand in black and the LDM models coloured based on their representative clusters defined in A. D) IFP of the representative LDM models and the X-ray structures. Interaction type is described for each residue of the binding pocket: hydrophobic interaction, hydrogen bond (H-bond) donor and acceptor, weak hydrogen bond (weak H-bond) donor and acceptor, ionic bond positive (+) and negative (-) and aromatic interaction. VS performance is described with ROC curves to visualise E) the recovery of B2AR agonists vs. decoys and F) the selectivity of B2AR agonists over B2AR inhibitors. The relative rank of the LDM refinement ligand is identified with a vertical dashed line. This vertical line may be masked by other curves if the ligand is very highly ranked. The ROC curve figure inset shows NSQ_AUC values for each binding pocket. Finally, a G) bar chart is used to visualise the EF for representative B2AR agonist chemotypes at EF1, EF5 and EF10. Chemotypes A, B ‘BI-like’, C ‘ADR-like’ and D ‘ISO-like’ represent only a subset of B2AR agonist ligands (S2C Fig). The EF bar chart inset shows the number of ligands for each chemotype cluster between parenthesis. Origin and destination X-ray structure chemotype EF shown in grey and black bars, respectively, with the LDM models coloured based on their relative clusters identified in A.

**Fig 8:**
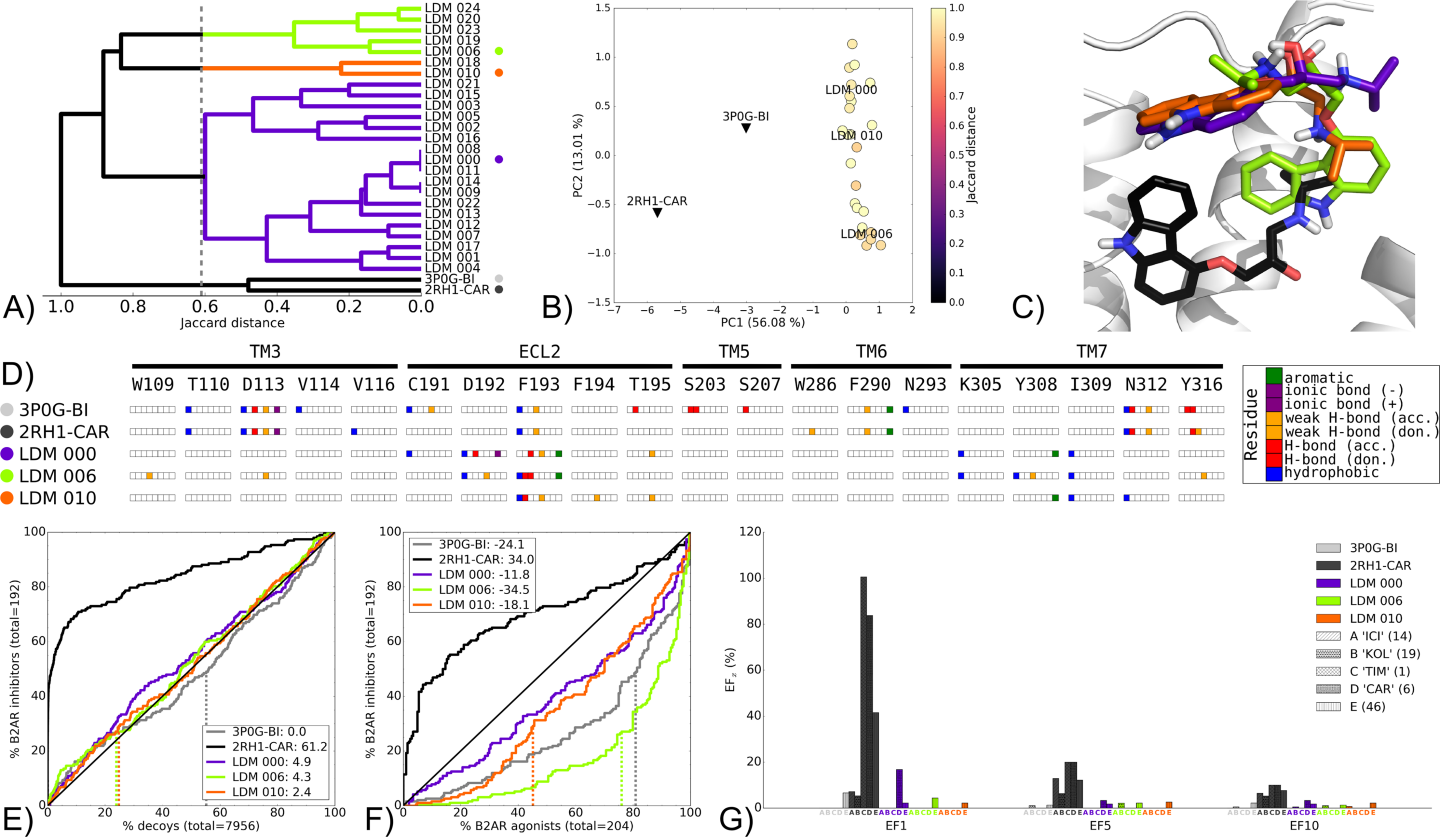
Pharmacology switch LDM experiment on the B2AR from 3P0G-BI to 2RH1-CAR, using CAR as a refinement ligand. A) Dendrogram of the top 25 LDM models and X-ray structures, a cutoff line identifies different LDM clusters and their representative LDM models are designated by a colored dot. Representative LDM models are the highest scoring within the cluster based on the OPUS-ICM metric. B) Comparison of binding pocket conformation between the top 25 LDM models and X-ray structures. LDM models are colored based on their IFP Jaccard distance with the destination X-ray structure, 2RH1-CAR. C) Binding poses of the representative LDM models and the destination X-ray structure with the X-ray ligand in black and the LDM models coloured based on their representative clusters defined in A. D) IFP of the representative LDM models and the X-ray structures. Interaction type is described for each residue of the binding pocket: hydrophobic interaction, hydrogen bond (H-bond) donor and acceptor, weak hydrogen bond (weak H-bond) donor and acceptor, ionic bond positive (+) and negative (-) and aromatic interaction. VS performance is described with ROC curves to visualise E) the recovery of B2AR inhibitors vs. decoys and F) the selectivity of B2AR inhibitors over B2AR agonists. The relative rank of the LDM refinement ligand is identified with a vertical dashed line. This vertical line may be masked by other curves if the ligand is very highly ranked. The ROC curve figure inset shows NSQ_AUC values for each binding pocket. Finally, a G) bar chart is used to visualise the EF for representative B2AR inhibitor chemotypes at EF1, EF5 and EF10. Chemotypes A ‘ICI-like’, B ‘KOL-like’, C ‘TIM-like’, D ‘CAR-like’ and E represent only a subset of B2AR inhibitor ligands (S2B Fig). The EF bar chart inset shows the number of ligands for each chemotype cluster between parenthesis. Origin and destination X-ray structure chemotype EF shown in grey and black bars, respectively, with the LDM models coloured based on their relative clusters identified in A.

Pharmacology switch LDM experiments represent the most complex task as they involve the largest binding pocket conformation rearrangement, from inhibitor-bound to agonist-bound or vice-versa. This includes the reorientation of residues that can interact with the bound ligand but also binding pocket backbone conformational changes. In all six pharmacology switch LDM experiments performed, a selected LDM model showed improved VS performance both in recovery and selectivity relative to the origin X-ray structure (Figs 7 and 8 and S20–S23 Figs). Additionally, these models had improved selectivity for the chemotype of the ligand used for refinement. Results of the pharmacology switch LDM scenario are divided into two groups that are explored in detail describing the two patterns identified.

#### Group A

This group includes LDM experiments for the B2AR and the M2R where pharmacology switch LDM experiments resulted in the LDM model sharing a similar binding pose to that of the destination X-ray structure. The experiments preformed include the M2R from inhibitor-bound 3UON-QNB refined using the agonist ligand found in 4MQS-IXO (S20 Fig) and the reverse experiment on M2R where 4MQS-IXO was refined with the ligand QNB, that was present in the X-ray structure 3UON-QNB (S21 Fig). The experiment chosen to describe this group is the B2AR LDM starting from the inhibitor CAR-bound X-ray structure 2RH1-CAR and using the refinement ligand BI167107 (BI) found in the destination X-ray structure 4LDE (Fig 7). The dendrogram of the ligand/receptor interaction patterns shows, as expected, separation of inhibitor-bound 2RH1-CAR and agonist-bound 4LDE-BI with each being located in two distinct clusters at a 0.55 Jaccard cutoff (Fig 7A). Two closely related groups of interaction patterns arose within the top 25 LDM models with the BI-bound X-ray structure (4LDE-BI) found in one of these clusters. The PCA plot provides a quantified representation of the collective binding pocket movement as a result of the LDM processing. The LDM models moved away from their origin X-ray structure 2RH1-CAR towards the destination X-ray structure 4LDE-BI, however, they did not reach the exact conformation of the destination X-ray structure due to insufficient sampling in PC1 (Fig 7B). The selected cluster representatives with the highest ranked scores within their respective IFP clusters (LDM 000 and LDM 004) had a similar pose to the destination 4LDE-BI (Fig 7C). The origin X-ray structure, destination X-ray structure and the LDM models all share a conserved ionic interaction between BI and D113, and a polar interaction was also shared with BI and N312 (Fig 7D). LDM 000 uniquely exhibited a similar polar interaction pattern with 4LDE-BI through S203 and S207, and it was the only complex to present a weak hydrogen bond and aromatic interaction between BI and Y316.

VS on these binding pockets showed that, expectedly, the agonist-bound 4LDE-BI is superior in recovery of B2AR agonists over decoys (NSQ_AUC: 70.4) relative to the inhibitor-bound 2RH1-CAR (NSQ_AUC: 28.0) (Fig 7E). This pattern was even more pronounced when comparing the two X-ray structures at B2AR agonist over inhibitor selectivity, with 4LDE-BI and 2RH1-CAR obtaining NSQ_AUC scores of 57.7 and -24.5, respectively (Fig 7F). Both screened LDM pockets outperformed the origin X-ray 2RH1-CAR in recovery and selectivity. Furthermore, LDM 000 achieved a high recovery NSQ_AUC score of 70.0, outperforming 4LDE-BI in early recovery. LDM 000 also outperformed 4LDE-BI in selectivity with a NSQ_AUC of 61.9. This superior VS performance can be explained in part by taking a closer look at the pattern of chemotype early enrichment. Indeed, LDM 000 was superior to both X-ray structures at recovering the B “BI-like” chemotype, with 21/26 identified at EF1 (Fig 7D). While LDM 004 showed improved VS performance over the origin structure, it did not reach the same VS performance as the destination X-ray structure or LDM 000. In addition, LDM 004 recovered the refinement ligand, BI, late in both VS recovery and selectivity (Fig 7E and 7F). But interestingly, it showed a different chemotype recovery pattern compared to the other binding pockets. There was no BI chemotype recovery and similar recovery as the origin, destination and LDM 000 in chemotype A recovery, however LDM 004 also displayed ISO chemotype ligands recovery at EF1, a feature not observed with the other structures or models (Fig 7D).

The other two LDM experiments from this group of pharmacology switch experiments refined the IXO-bound (agonist) M2R to the QNB-bound (inhibitor) conformation (S21 Fig), and vice-versa (S20 Fig). The interaction pattern distance between IXO-bound and QNB-bound complexes (S20A and S21A Figs) is smaller than between CAR-bound and BI-bound X-ray structures (Fig 7A). This did not influence convergence of LDM complexes for refinement of IXO-bound with QNB as the top 25 QNB-refined models were closely related (S21A Fig). In contrast, QNB-bound structures refined by the small ligand IXO resulted in LDM models with three distinct ligand poses. In both cases however, there were representative LDM models that had a similar ligand pose to the destination X-ray structure (S20A, S20C, S20D, S21A, S21C and S21D Figs). Amongst these, LDM models outperformed the origin X-ray structure in VS recovery (S20E and S21E Figs), selectivity (S20F and S21F Figs) and refinement ligand enrichment (S20G and S21G Figs). However, they did not outperform the destination X-ray structure. In both cases, the best scoring LDM model (LDM 000) showed improved enrichment for the chemotype of the LDM refinement ligand relative to both the origin and destination X-ray structures.

#### Group B

In the second group of pharmacology switch experiments, each of the best performing LDM models had a distinct ligand pose to that of the destination X-ray structure, but exhibited superior performance in VS relative to the origin X-ray structure. Within this group are LDM experiments on the AA2AR inhibitor-bound 3EML-ZM refined with the ligand NEC in the agonist-bound X-ray structure AA2AR-NEC (S22 Fig) and the AA2AR agonist-bound 3QAK-UK refined with the ligand ZM found in the inhibitor-bound X-ray structure 3EML-ZM (S23 Fig). The third LDM experiment from this group described in more detail here was performed on the B2AR agonist-bound 3P0G-BI refined using the inhibitor CAR, which can be found in the X-ray structure 2RH1-CAR (Fig 8). Interestingly, the two X-ray structures in this example that are bound by ligands of different pharmacology, grouped together in the interaction pattern dendrogram (Fig 8A). Indeed, the X-ray structures had a similar ligand interaction pattern with residues D113, F290, N312 and Y316 (Fig 8D). Notably, none of the representative LDM conformations exhibited this interaction pattern. Despite the absence of the ionic interaction with D113, an ionic interaction was identified in LDM 000 between the tertiary amine of CAR and D192.

The top 25 LDM models clustered into three groups with the largest convergence represented by LDM 000, followed by two smaller clusters each represented by LDM 006 and LDM 010 (Fig 8A). Interestingly, the collective conformational change in binding pockets induced by the LDM moves from 3P0G-BI away from 2RH1-CAR along PC1 (Fig 8B). However, in PC2, which explains an additional 13.01% of the ensemble’s binding pocket conformation variation, a movement towards the destination X-ray structure 2RH1-CAR is observed. Viewing the representative LDM binding poses revealed alternative ligand and binding pocket conformations to that of the 2RH1-CAR (Fig 8C).

The destination X-ray structure 2RH1-CAR displayed high performance in B2AR inhibitor recovery over decoys with a NSQ_AUC of 61.2, while the origin agonist-bound X-ray structure 3P0G-BI performed the same as random at 0.0 NSQ_AUC. While the representative LDM models did improve on the performance of the origin X-ray structure, they performed only slightly better than random, but notably they all recovered CAR earlier than the origin X-ray structure (Fig 8E). In terms of B2AR inhibitor over agonist selectivity, each X-ray structures was selective for the pharmacology of their bound ligand. In this case both LDM 000 and LDM 010 improved on the inhibitor selectivity compared to the origin X-ray structure, although both were still more selective towards agonists (Fig 8F). Looking at the chemotype enrichment, LDM 000 recovered 1/6 ligands from the D ‘CAR-like’ chemotype at EF1. This corresponds to the CAR ligand itself that was highly rank by LDM 000, however this was still an improvement compared to the origin X-ray structure 3P0G-BI that was unable to recover any CAR-like ligands, including CAR itself. The destination X-ray structure 2RH1-CAR, however, had a better EF1 value for the D ‘CAR-like’ chemotype with 5/6 and had overall a more versatile chemotype enrichment (Fig 8G).

The other two additional LDM experiments that made up this second pharmacology switch group were both performed on AA2AR. In each case, the interaction pattern distance between agonist-bound and inhibitor-bound complexes was greater than in the CAR-refinement experiment described above (S22A and S23A Figs). While the NEC-refined LDM models featured two distinct ligand poses (S22A, S22C and S22D Fig), the ZM-refined models converged towards a single one (S23A, S23C and S23D Fig). In both cases, these LDM complexes featured ligand poses that were different from their respective destination X-ray structures. Despite this, they displayed improved VS performance for recovery of known ligands over decoys (S22E and S23E Figs), selectivity (S22F and S23F Figs) and refinement ligand chemotype enrichment (S22G and S23G Figs) relative to their origin X-ray structures.

## Discussion

The potential of *in silico* virtual screening for GPCRs is limited by poor representation of conformational sampling of these receptors in experimentally derived structures and homology models. Here we have developed an LDM method using a computational workflow that refines a GPCR binding pocket around a known ligand for that GPCR using iterative rounds of conformational sampling and scoring methods to identify low energy minimums of the system. Each LDM experiment in this study generated close to 1 million GPCR/ligand complexes and outputted up to 320 complexes as selected by its scoring method. In contrast with MD-based methods, the LDM readily overcomes energy barriers that hamper the transition between conformational states as it relies on geometric sampling methods. Additionally, the workflow is highly parallelised running on multiple independent processes simultaneously that greatly accelerates the process of generating GPCR-ligand complexes compared to other available methods.

The performance of the LDM method was evaluated by designing LDM experiments where a GPCR X-ray binding pocket was refined using a ligand that was available in another GPCR X-ray co-crystal structure. Each LDM experiment thus consisted of a comparison between origin X-ray structure, LDM model(s) and destination X-ray structure in VS performance and GPCR/ligand conformation. For most LDM experiments performed in this study, the top 25 GPCR/ligand complexes of these LDM outputs contained a complex with a small heavy atom ligand root-mean-square deviation (RMSD) difference relative to the destination X-ray complex (S3 Table). Indeed, the LDM method successfully generated a ligand conformation that closely resembled the binding pose of the ligand in a solved X-ray structure in 18 out of 24 cases highlighting the overall success of the sampling performed by the LDM method. Interestingly, assessment of binding pocket conformation using PCA revealed that while agonist and inhibitor-bound X-ray binding pockets can be distinguished from one another (S1A and S1B Fig), the change in binding pocket conformation from origin to destination X-ray structure is not consistently indicative of improved VS performance and therefore additional metrics are required to evaluate LDM performance.

The scoring of LDM complexes uses both quantitative and qualitative scoring methods. During the LDM process, a quantitative scoring method is applied on GPCR/ligand complexes generated to iteratively select the most suitable candidate before it is used as the starting point for further sampling. The same quantitative ligand docking and protein geometry scores are combined in equal weights to generate a score that ranks the ~320 LDM results. As evidenced by the data in this study, the most highly scored LDM complex (LDM 000) was not consistently the best performer in retrospective VS. However, a complex was found within the top 25 ranked complexes in most LDM experiments that did outperform the origin X-ray structures, and even in some cases the destination X-ray structure, suggesting that the quantitative scoring method used is relevant for identification of a suitable binding pocket for VS. An additional qualitative IFP scoring method was employed for clustering based on GPCR/ligand interaction patterns. Although other more complex implementations of IFP were shown to perform better when used for scoring docked poses [83], we showed that the IFP implementation used in this study is useful for the identification of a GPCR binding pocket with good VS performance [72]. Furthermore, here we show that the IFP can capture differences between agonist-bound and inhibitor-bound X-ray structures (S1C and S1D Fig), as well as between structures and models that can distinguish between distinct ligand chemotypes. Using a combination of the quantitative scoring method and the qualitative IFP scoring was more effective at selecting LDM models for improved overall retrospective VS performance over the origin X-ray structure than the quantitative scoring alone with this method identifying improved binding pockets in 20/24 of the LDM experiments performed.

To evaluate the success of the LDM, LDM optimised GPCR binding pockets were screened with known ligands and decoys libraries developed by Cavasotto et al [70]. Known agonists or inhibitors for a given GPCR often share a common chemotype (S2 Fig). A key finding from this study was the ability of the refinement ligand used within the LDM method to influence the result of the LDM binding pocket’s recovery or selectivity. In some cases, this resulted in the LDM models being superior to the origin X-ray structure and in some cases, also the destination structure, observations that were most evident in the pharmacology switch studies (S3 Table). However, there were also occurrences of LDM models that outperformed the destination, but not the origin X-ray structure, despite the refinement ligand being recovered earlier in the VS with the LDM models. This scenario was observed in some of the chemotype switch LDM experiments. From these observations, it was apparent that when the refinement is performed with a ligand whose chemotype is highly represented amongst known ligands, a superior retrospective VS outcome is observed. In contrast, when the refinement ligand chemotype is poorly represented among known ligands, this often appears to result in an inferior retrospective VS outcome. The chemotype enrichment feature used in this study that evaluates the EF values for a representative set of chemotypes is an attractive tool to improve understanding of binding pocket ligand recognition in a VS setting. This revealed that in all cases, the LDM generates binding pockets that are either improved for identification of chemotypes related to the refinement ligand or narrowed in the identification towards the chemotype of the refinement ligand at the exclusion of other chemotypes, when compared to the origin X-ray structure (Table 1). Therefore, the LDM generates binding pockets that are more specific in their ligand recovery of particular chemotypes. This highlights the success of the LDM as the method is aimed at refining the binding pocket around a particular ligand, however this result also poses additional challenges and considerations around utilisation of LDM methods for prospective VS. Therefore, to maximise the chances of identifying new hits in a prospective VS, LDM refinement of GPCR X-ray structures using multiple known ligands with distinct chemotypes would generate a combination of LDM binding pockets, each biased to select for a complementary set of ligand chemotypes. This would likely result in improved VS outcomes, both in terms of numbers of compounds, but also with increased hit chemotype variety. In some cases, the chemotype preference of certain LDM models could even be leveraged in a SBDD program, for example using ligands exhibiting novel or underrepresented chemotypes, where the identification of novel scaffold is of paramount importance in the search of new lead compounds.

**Table 1:** Summary of the LDM benchmark outcomes. The results are organised by scenario: self refinement, chemotype switch and pharmacology switch. Each line describes an LDM experiment including the origin and destination X-ray structures as well as the overall VS performance outcome. The best LDM model is compared to the origin X-ray structure in recovery of known ligands vs. decoys, selectivity of agonists over inhibitors (or vice-versa) and chemotype enrichment. VS recovery and selectivity performance is improved ↑, similar → or worse ↓ using NSQ_AUC values. VS chemotype enrichment evaluates the LDM refinement ligand chemotype enrichment by comparing the EF1 values of LDM models with their origin X-ray structure. It is “improved” when an LDM model outperforms the origin X-ray structure, and it is “narrowed” when the same performance is observed and EF1 value for other chemotypes is worse for the LDM model. LDM experiments were assigned to two groups based on the similarity of their LDM model binding pose to that of the destination X-ray structure and their improvement in VS performance over the origin X-ray structure. Group A includes LDM models with similar binding poses and improved performance and group B includes LDM models with a different binding pose and improved performance or a similar binding pose and no performance improvement. CCR5 agonist ligands were not available from the GLL/GDD, hence VS selectivity for the self refinement CCR5-MRV experiment was not calculated and is marked N/A.

LDM benchmark scenarios	GPCR	Origin X-ray	Destination X-ray	Group	VS recovery	VS selectivity	VS chemotype enrichment	Figure
Self refinement	B2AR	4LDE-BI (ago.)	-	A	↑	↑	improved	Fig 3
	B2AR	3P0G-BI (ago.)	-	A	↑	↑	improved	S5 Fig
	AA2AR	4EIY-ZM (inhib.)	-	A	→	↑	narrowed	S6 Fig
	AA2AR	3PWH-ZM (inhib.)	-	A	→	↓	narrowed	S7 Fig
	M2R	3UON-QNB (inhib.)	-	A	→	↑	narrowed	S8 Fig
	M2R	4MQS-IXO (ago.)	-	A	↑	↑	narrowed	S9 Fig
	H1R	3RZE-DOX (inhib.)	-	A	→	↑	improved	S10 Fig
	5-HT1B	4IAR-ERG (inhib.)	-	A	↓	↑	narrowed	S11 Fig
	B2AR	2RH1-CAR (inhib.)	-	B	↓	↓	improved	Fig 4
	AA2AR	2YDV-NEC (ago.)	-	B	↓	↓	narrowed	S12 Fig
	CCR5	4MBS-MRV (inhib.)	-	B	→	N/A	narrowed	S13 Fig
	DOR	4N6H-NAL (inhib.)	-	B	↑	↑	improved	S14 Fig
Chemotype switch	B2AR	4LDO-ADR (ago.)	4LDL-ISO (ago.)	A	↑	↑	improved	Fig 5
	B2AR	3NY9-KOL (inhib.)	2RH1-CAR (inhib.)	A	↑	↑	improved	S15 Fig
	AA2AR	3QAK-UK (ago.)	2YDV-NEC (ago.)	A	↑	↑	improved	S16 Fig
	B2AR	3D4S-TIM (inhib.)	3NY8-ICI (inhib.)	A	↓	↑	narrowed	S17 and S18 Figs
	AA2AR	3PWH-ZM (inhib.)	3RFM-CAF (inhib.)	B	↑	↑	improved	Fig 6
	AA2AR	3UZA-T4G (inhib.)	3REY-XAC (inhib.)	B	↑	↑	improved	S19 Fig
Pharmacology switch	B2AR	2RH1-CAR (inhib.)	4LDE-BI (ago.)	A	↑	↑	improved	Fig 7
	M2R	3UON-QNB (inhib.)	4MQS-IXO (ago.)	A	↑	↑	improved	S20 Fig
	M2R	4MQS-IXO (ago.)	3UON-QNB (inhib.)	A	↑	↑	improved	S21 Fig
	B2AR	3P0G-BI (ago.)	2RH1-CAR (inhib.)	B	↑	↑	improved	Fig 8
	AA2AR	3EML-ZM (inhib.)	2YDV-NEC (ago.)	B	↑	↑	narrowed	S22 Fig
	AA2AR	3QAK-UK (ago.)	3EML-ZM (inhib.)	B	↑	↑	improved	S23 Fig

With the exception of a few receptors (for example B2AR, AA2A and M2R), the majority of GPCR X-ray structures are solved in inactive conformations, bound to inhibitor ligands. However, for many of these receptors, novel agonists are highly sought for drug development. As shown for the B2AR and AA2AR, GPCR binding pockets can be clustered based on their GPCR-ligand interaction pattern and their shapes that clearly separate agonist-bound from inhibitor-bound pockets (S1 Fig). The ability of the LDM to influence the types of ligand chemotype recovered in VS reveals a very powerful application of the LDM method for SBDD. Largely due to this feature, all pharmacology switch LDM experiments were successful in improving ligand selectivity (i.e. agonist vs inhibitor or vice-versa) relative to their origin X-ray structure, while also improving recovery of known ligands over decoys (Table 1). The pharmacology switch scenario represents the largest conformational change between origin and destination structures and the success of the method in terms of VS performance highlights the power and full potential of this LDM method for SBDD.

Within this study, there were some LDM experiments that were successful in terms of their VS performance, but where the LDM generated a binding pose different to that of the destination X-ray structure. The absence of convergence towards the putative global energy minimum represented by the destination X-ray structure could be due to several factors. While the LDM sampling and scoring scheme may have failed to converge towards the native binding pose, other factors may also be at play. During the docking stage of the LDM workflow, the ligand conformation search space is increased if the ligand is very small or if it contains many degrees of freedom. This may lead to an insufficient exploration of the ligand’s native binding pose, which can result in poor convergence of the LDM workflow (Fig 6 and S13 Fig). Furthermore, two LDM experiments (B2AR refined with CAR (Fig 4) and B2AR refined with ICI (S17 Fig)) featured inhibitor binding poses that were located above the X-ray binding pose in a vestibule formed by ECL2 and the top of TM 5–7 (Fig 4C and S17C Fig). This corresponds to a vestibule identified by Dror et al. in an unbiased molecular dynamics simulations performed with four different B2AR ligands [84]. Within these simulations each ligand associated with this vestibule, and exit from this pocket provided the largest energetic barrier for the ligand entry into the predominant binding pocket. These resulting LDM models may thus represent a valid but transient ligand pose. The identification of this transient ligand pose may have benefited from the inclusion of ECLs other than ECL2-distal, which are omitted in the LDM workflow to improve computational efficiency and focussed sampling of the binding pocket. However, this inclusion would not have contributed to LDM refinement goal, which is to identify the final ligand pose that initiates receptor activity and is observed in X-ray structures. Indeed, VS performance does not seem to be affected by the presence of ECLs other than ECL2-distal for X-ray structures used in this study, including B2AR (Fig 2). Although this may be true for most Class A GPCR orthosteric ligands, ECLs may be crucial for the binding of some ligands for example allosteric modulators of Class A GPCRs [22,85]. Thus, in some scenarios, loops should be built onto LDM models, for example if the end goal was to screen for allosteric ligands binding in the extracellular loop region. Finally, it should be noted that the LDM workflow does not take into account water molecules that can mediate and/or influence ligand/receptor interactions [86,87]. This may hinder the ability of the LDM workflow to identify a native ligand/receptor conformation that relies on such an interaction, as may be the case for opioid receptor bound by NAL (S14 Fig). Future improvements of our LDM method could take binding pocket waters into account, however this would be at a significant computational cost.

A range of different methods exist for the refinement of receptor models prior to prospective VS in GPCR SBDD. The conformational changes induced by ligand binding and receptor activation can be observed using unbiased MD [37,84]. However these microsecond timescale events require running these simulations over several days on special purpose hardware [88]. An alternative method uses multiple shorter MD runs stitched together using Markov state modeling and although this has been shown to be applicable to SBDD, large computing resources are still needed to develop such a statistical model [41]. And although progress has been made on enhanced sampling techniques such as accelerated MD which was applied to observe large GPCR conformational re-arrangements [89,90] and contributed to the preparation of receptor models for prospective VS [40], these methods still require several days of computing time to reach this result. In contrast, conventional MD has only been used to reach limited conformational changes for GPCR refinement [38,39], which have nonetheless seen applications in SBDD (e.g. [91,92]). A general issue that remains with MD simulations is the fact they are not designed to rapidly converge towards low energy minimums, which can lead to difficulties in identifying the receptor conformations that should be used for a subsequent VS. Other more computationally efficient methods such as the iterative use of the induced fit docking (IFD) protocol has also been successfully applied to refine GPCR binding pockets towards improved VS performance (e.g. [47,48]). However, these methods are not well suited to overcome the large energy barriers associated with the conformational rearrangements that occur during GPCR activation. Automated methods using alternative sampling strategies combined with molecular docking are better suited to such a task, such as that developed by Nguyen at al. [53]. This study generated models for a set of 14 available GPCRs X-ray structures, however it was validated only by structural comparisons to X-ray structures and no VS performance evaluation of the models was performed. Another such method is ALiBERO [50], which has been applied to generate neurotensin receptor 1 (NTSR1) models that outperformed the recently solved NTSR1 X-ray structure in a retrospective VS [51], and refine a homology model of the 5-hydroxytryptamine receptor 1A (5HT1A) that was used in a prospective VS to identify two new active compounds at the 5HT1A [52]. However, ALiBERO relies on the availability of multiple known binders and non-binders or decoys for the GPCR to be refined. This data is unavailable for many GPCR targets of therapeutic relevance, highlighting the importance the current LDM approach as a GPCR refinement method that requires only a single ligand for binding pocket refinement. Lastly, ALiBERO relies on the refinement of a binding pocket ensemble and a similar approach can be undertaken with the current LDM. LDM models show enrichment for the chemotype of their refinement ligand and as described above, an ensemble of carefully selected LDM models that can enrich for a complementary set of ligand chemotypes may greatly improve VS outcome in identifying hits of diverse chemistries.

## Conclusions

We present a new computational workflow that refines a GPCR binding pocket using a known ligand for that GPCR. This is achieved though an iterative process that relies on computationally inexpensive and highly parallelised methods including docking and geometric protein sampling. Thus, the LDM achieves relatively fast refinement of GPCR binding pockets and outputs LDM models that are ranked based on a quantitative docking and protein geometry score, and can be clustered based on a qualitative score, ligand/receptor interaction pattern. The LDM was evaluated with an extensive benchmark of 24 LDM experiments where in each experiment the binding pocket of an origin X-ray structure was refined using the ligand from a destination X-ray structure. These experiments were divided into three scenarios of increasing distance between the origin and destination X-ray structures, where the most complex task performs refinement from an inhibitor-bound to an agonist-bound binding pocket (or vice-versa). Overall an LDM model with ligand pose similar to that of the destination was reported in 18 out of 24 experiments, and an LDM model with improved VS performance over the origin X-ray structure was reported in 20 out of 24 experiments. One of the key findings of this study is that LDM models are found to be more selective for the chemotype of the ligand that was used for their binding pocket refinement. This may be a key factor in the LDM’s success for all pharmacology switch experiments, a valued feature for SBDD programs that seek agonists on targets that only have inhibitor-bound experimentally determined structures. But this chemotype preference of LDM models could also be leveraged to influence the identification of specific scaffolds in a prospective VS setting.

## Supporting information

S1 Table Description of the LDM method and parameters. Description of the method used at each step of the LDM workflow. Parameters used in the current study for each of these methods. CONCOORD parameters that were common in both step where the software is used: secondary conformation detection: DSSP, forcefield: OPLS-UA (united atom), bond and angle parameters: CONCOORD default.
(PDF)

S2 Table Information about X-ray structures used in the study. Details about ligand name and structure as well as mutations in the TM bundle and their proximity to the X-ray ligand.
(PDF)

S3 Table LDM models and X-ray structure ligand RMSD and NSQ_AUC values. Selected LDM models and X-ray origin and destination structures are compared by ligand heavy atom RMSD to the destination X-ray structure and VS performance by recovery NSQ_AUC and selectivity NSQ_AUC values.
(PDF)

S1 Text Executing the LDM scripts. Description of the required software, file preparation and parameters to run the LDM.
(PDF)

S1 Fig Agonist and inhibitor-bound X-ray binding pocket comparisons for B2AR and AA2AR. Principal component analysis on the binding pocket residue coordinates of A) B2AR and B) AA2AR X-ray structures used in this study. Principal components (e.g. PC1 and PC2) are ordered based on their percentage of cumulative variance explained from the original dataset (e.g. PC1 explains 50% of the data variance). An arbitrary line drawn along the PC1 axis, which explains most of the variance in the dataset, separates agonist-bound and inhibitor-bound X-ray structures based on their binding pocket conformations. Dendrogram of the IFP clustering and IFP diagrams on the ligand/receptor interaction patterns for C) B2AR and D) AA2AR X-ray structures used in this study. The IFP dendrogram branches and the PCA labels representing agonist-bound and inhibitor-bound X-ray structures are colored green and red, respectively.
(TIF)

S2 Fig Known agonist and inhibitor ligand library chemotypes. Ligand libraries were clustered based on pharmacophore properties and 0.5 cutoff was used to identify the different clusters. A limited number of representative chemotype clusters were identified by letters (A-E). Chemotype clusters that contain an X-ray ligand were chosen and additional populated clusters were added when increased chemotype coverage was necessary. The resulting chemotype clusters do not contain the entire ligand library but are used to represent its chemotype diversity. AA2AR agonists (A) and inhibitors (B), B2AR agonists (C) and inhibitors (D), M2R agonists (E) and inhibitors (F), CCR5 inhibitors (G), DOR inhibitors (H), H1R inhibitors (I), 5-HT1B inhibitors (J).
(PDF)

S3 Fig ROC curves of VS performance for X-ray structures with all loops vs. ECL2-distal loop only. VS performance evaluated as recovery of known ligands against decoys and selectivity of agonists over inhibitors (or vice-versa). All loop structures are represented by black dotted lines and ECL2-distal only structures are represented by solid black lines. NSQ_AUC values for each curve is shown in the figure inset. AA2AR 2YDV-NEC recovery (A) and selectivity (B), AA2AR 3QAK-UK recovery (C) and selectivity (D), AA2AR 3EML-ZM recovery (E) and selectivity (F), AA2AR 3PWH-ZM recovery (G) and selectivity (H), AA2AR 4EIY-ZM recovery (I) and selectivity (J), AA2AR 3REY-XAC recovery (K) and selectivity (L), AA2AR 3RFM-CAF recovery (M) and selectivity (N), AA2AR 3UZA-T4G recovery (O) and selectivity (P), B2AR 3P0G-BI recovery (Q) and selectivity (R), B2AR 4LDE-BI recovery (S) and selectivity (T), B2AR 4LDL-ISO recovery (U) and selectivity (V), B2AR 4LDO-ADR recovery (W) and selectivity (X).
(PDF)

S4 Fig ROC curves of VS performance for X-ray structures with all loops vs. ECL2-distal loop only. VS performance evaluated as recovery of known ligands against decoys and selectivity of agonists over inhibitors (or vice-versa). All loop structures are represented by black dotted lines and ECL2-distal only structures are represented by solid black lines. NSQ_AUC values for each curve is shown in the figure inset. B2AR 2RH1-CAR recovery (A) and selectivity (B), B2AR 3D4S-TIM recovery (C) and selectivity (D), B2AR 3NY8-ICI recovery (E) and selectivity (F), B2AR 3NY9-KOL recovery (G) and selectivity (H), M2R 4MQS-IXO recovery (I) and selectivity (J), M2R 3UON-QNB recovery (K) and selectivity (L), 5-HT1B 4IAR-ERG recovery (M) and selectivity (N), DOR 4N6H-NAL (3D library) recovery (O) and selectivity (P), H1R 3RZE-DOX recovery (Q) and selectivity (R), CCR5 4MBS-MRV recovery (S).
(PDF)

S5 Fig Self refinement LDM experiment on the B2AR 3P0G-BI, using BI as a refinement ligand. A) Dendrogram of the top 25 LDM models and X-ray structure(s), a cutoff line identifies different LDM clusters and their representative LDM models are designated by a colored dot. Representative LDM models are the highest scoring within the cluster based on the OPUS-ICM metric. B) Comparison of binding pocket conformation between the top 25 LDM models and X-ray structure(s). LDM models are colored based on their IFP Jaccard distance with the destination X-ray structure. C) Binding poses of the representative LDM model(s) and the destination X-ray structure. D) IFP of the representative LDM models and the X-ray structure. Interaction type is described for each residue of the binding pocket: hydrophobic interaction, hydrogen bond (H-bond) donor and acceptor, weak hydrogen bond (weak H-bond) donor and acceptor, ionic bond positive (+) and negative (-) and aromatic interaction. E) the recovery of known ligands vs. decoys and F) the selectivity of inhibitors over agonists (or vice-versa). The relative rank of the LDM refinement ligand is identified with a vertical dashed line. This vertical line may be masked by other curves if the ligand is very highly ranked. The ROC curve figure inset shows NSQ_AUC values for each binding pocket. Finally, a G) bar chart is used to visualise the EF for representative known ligand chemotypes at EF1, EF5 and EF10. Chemotypes A, B ‘BI-like’, C ‘ADR-like’ and D ‘ISO-like’ represent only a subset of B2AR agonist ligands (S2 Fig). The EF bar chart inset shows the number of ligands for each chemotype cluster between parenthesis. X-ray structure chemotype EF shown in black bars, with the LDM models coloured based on their relative clusters identified in A.
(TIF)

S6 Fig Self refinement LDM experiment on the AA2AR 4EIY-ZM, using ZM as a refinement ligand. A) Dendrogram of the top 25 LDM models and X-ray structure(s), a cutoff line identifies different LDM clusters and their representative LDM models are designated by a colored dot. Representative LDM models are the highest scoring within the cluster based on the OPUS-ICM metric. B) Comparison of binding pocket conformation between the top 25 LDM models and X-ray structure(s). LDM models are colored based on their IFP Jaccard distance with the destination X-ray structure. C) Binding poses of the representative LDM model(s) and the destination X-ray structure. D) IFP of the representative LDM models and the X-ray structure. Interaction type is described for each residue of the binding pocket: hydrophobic interaction, hydrogen bond (H-bond) donor and acceptor, weak hydrogen bond (weak H-bond) donor and acceptor, ionic bond positive (+) and negative (-) and aromatic interaction. VS performance is described with ROC curves to visualise E) the recovery of known ligands vs. decoys and F) the selectivity of inhibitors over agonists (or vice-versa). The relative rank of the LDM refinement ligand is identified with a vertical dashed line. This vertical line may be masked by other curves if the ligand is very highly ranked. The ROC curve figure inset shows NSQ_AUC values for each binding pocket. Finally, a G) bar chart is used to visualise the EF for representative known ligand chemotypes at EF1, EF5 and EF10. Chemotypes A ‘ZM-like’, B ‘T4G-like’, C ‘XAC-like’ and D ‘CAF-like’ represent only a subset of AA2AR inhibitors ligands (S2 Fig). The EF bar chart inset shows the number of ligands for each chemotype cluster between parenthesis. X-ray structure chemotype EF shown in black bars, with the LDM models coloured based on their relative clusters identified in A.
(TIF)

S7 Fig Self refinement LDM experiment on the AA2AR 3PWH-ZM, using ZM as a refinement ligand. A) Dendrogram of the top 25 LDM models and X-ray structure(s), a cutoff line identifies different LDM clusters and their representative LDM models are designated by a colored dot. Representative LDM models are the highest scoring within the cluster based on the OPUS-ICM metric. B) Comparison of binding pocket conformation between the top 25 LDM models and X-ray structure(s). LDM models are colored based on their IFP Jaccard distance with the destination X-ray structure. C) Binding poses of the representative LDM model(s) and the destination X-ray structure. D) IFP of the representative LDM models and the X-ray structure. Interaction type is described for each residue of the binding pocket: hydrophobic interaction, hydrogen bond (H-bond) donor and acceptor, weak hydrogen bond (weak H-bond) donor and acceptor, ionic bond positive (+) and negative (-) and aromatic interaction. VS performance is described with ROC curves to visualise E) the recovery of known ligands vs. decoys and F) the selectivity of inhibitors over agonists (or vice-versa). The relative rank of the LDM refinement ligand is identified with a vertical dashed line. This vertical line may be masked by other curves if the ligand is very highly ranked. The ROC curve figure inset shows NSQ_AUC values for each binding pocket. Finally, a G) bar chart is used to visualise the EF for representative known ligand chemotypes at EF1, EF5 and EF10. Chemotypes A ‘ZM-like’, B ‘T4G-like’, C ‘XAC-like’ and D ‘CAF-like’ represent only a subset of AA2AR inhibitors ligands (S2 Fig). The EF bar chart inset shows the number of ligands for each chemotype cluster between parenthesis. X-ray structure chemotype EF shown in black bars, with the LDM models coloured based on their relative clusters identified in A.
(TIF)

S8 Fig Self refinement LDM experiment on the M2R 3UON-QNB, using QNB as a refinement ligand. A) Dendrogram of the top 25 LDM models and X-ray structure(s), a cutoff line identifies different LDM clusters and their representative LDM models are designated by a colored dot. Representative LDM models are the highest scoring within the cluster based on the OPUS-ICM metric. B) Comparison of binding pocket conformation between the top 25 LDM models and X-ray structure(s). LDM models are colored based on their IFP Jaccard distance with the destination X-ray structure. C) Binding poses of the representative LDM model(s) and the destination X-ray structure. D) IFP of the representative LDM models and the X-ray structure. Interaction type is described for each residue of the binding pocket: hydrophobic interaction, hydrogen bond (H-bond) donor and acceptor, weak hydrogen bond (weak H-bond) donor and acceptor, ionic bond positive (+) and negative (-) and aromatic interaction. VS performance is described with ROC curves to visualise E) the recovery of known ligands vs. decoys and F) the selectivity of inhibitors over agonists (or vice-versa). The relative rank of the LDM refinement ligand is identified with a vertical dashed line. This vertical line may be masked by other curves if the ligand is very highly ranked. The ROC curve figure inset shows NSQ_AUC values for each binding pocket. Finally, a G) bar chart is used to visualise the EF for representative known ligand chemotypes at EF1, EF5 and EF10. Chemotypes A, B ‘QNB-like’ and C represent only a subset of M2R inhibitor ligands (S2 Fig). The EF bar chart inset shows the number of ligands for each chemotype cluster between parenthesis. X-ray structure chemotype EF shown in black bars, with the LDM models coloured based on their relative clusters identified in A.
(TIF)

S9 Fig Self refinement LDM experiment on the M2R 4MQS-IXO, using IXO as a refinement ligand. A) Dendrogram of the top 25 LDM models and X-ray structure(s), a cutoff line identifies different LDM clusters and their representative LDM models are designated by a colored dot. Representative LDM models are the highest scoring within the cluster based on the OPUS-ICM metric. B) Comparison of binding pocket conformation between the top 25 LDM models and X-ray structure(s). LDM models are colored based on their IFP Jaccard distance with the destination X-ray structure. C) Binding poses of the representative LDM model(s) and the destination X-ray structure. D) IFP of the representative LDM models and the X-ray structure. Interaction type is described for each residue of the binding pocket: hydrophobic interaction, hydrogen bond (H-bond) donor and acceptor, weak hydrogen bond (weak H-bond) donor and acceptor, ionic bond positive (+) and negative (-) and aromatic interaction. VS performance is described with ROC curves to visualise E) the recovery of known ligands vs. decoys and F) the selectivity of inhibitors over agonists (or vice-versa). The relative rank of the LDM refinement ligand is identified with a vertical dashed line. This vertical line may be masked by other curves if the ligand is very highly ranked. The ROC curve figure inset shows NSQ_AUC values for each binding pocket. Finally, a G) bar chart is used to visualise the EF for representative known ligand chemotypes at EF1, EF5 and EF10. Chemotypes A, B and C ‘IXO-like’ represent only a subset of M2R agonist ligands (S2 Fig). The EF bar chart inset shows the number of ligands for each chemotype cluster between parenthesis. X-ray structure chemotype EF shown in black bars, with the LDM models coloured based on their relative clusters identified in A.
(TIF)

S10 Fig Self refinement LDM experiment on the H1R 3RZE-DOX, using DOX as a refinement ligand. A) Dendrogram of the top 25 LDM models and X-ray structure(s), a cutoff line identifies different LDM clusters and their representative LDM models are designated by a colored dot. Representative LDM models are the highest scoring within the cluster based on the OPUS-ICM metric. B) Comparison of binding pocket conformation between the top 25 LDM models and X-ray structure(s). LDM models are colored based on their IFP Jaccard distance with the destination X-ray structure. C) Binding poses of the representative LDM model(s) and the destination X-ray structure. D) IFP of the representative LDM models and the X-ray structure. Interaction type is described for each residue of the binding pocket: hydrophobic interaction, hydrogen bond (H-bond) donor and acceptor, weak hydrogen bond (weak H-bond) donor and acceptor, ionic bond positive (+) and negative (-) and aromatic interaction. VS performance is described with ROC curves to visualise E) the recovery of known ligands vs. decoys and F) the selectivity of inhibitors over agonists (or vice-versa). The relative rank of the LDM refinement ligand is identified with a vertical dashed line. This vertical line may be masked by other curves if the ligand is very highly ranked. The ROC curve figure inset shows NSQ_AUC values for each binding pocket. Finally, a G) bar chart is used to visualise the EF for representative known ligand chemotypes at EF1, EF5 and EF10. Chemotypes A ‘DOX-like’, B, C and D represent only a subset of H1R inhibitor ligands (S2 Fig). The EF bar chart inset shows the number of ligands for each chemotype cluster between parenthesis. X-ray structure chemotype EF shown in black bars, with the LDM models coloured based on their relative clusters identified in A.
(TIF)

S11 Fig Self refinement LDM experiment on the 5-HT1B 4IAR-ERG, using ERG as a refinement ligand. A) Dendrogram of the top 25 LDM models and X-ray structure(s), a cutoff line identifies different LDM clusters and their representative LDM models are designated by a colored dot. Representative LDM models are the highest scoring within the cluster based on the OPUS-ICM metric. B) Comparison of binding pocket conformation between the top 25 LDM models and X-ray structure(s). LDM models are colored based on their IFP Jaccard distance with the destination X-ray structure. C) Binding poses of the representative LDM model(s) and the destination X-ray structure. D) IFP of the representative LDM models and the X-ray structure. Interaction type is described for each residue of the binding pocket: hydrophobic interaction, hydrogen bond (H-bond) donor and acceptor, weak hydrogen bond (weak H-bond) donor and acceptor, ionic bond positive (+) and negative (-) and aromatic interaction. VS performance is described with ROC curves to visualise E) the recovery of known ligands vs. decoys and F) the selectivity of inhibitors over agonists (or vice-versa). The relative rank of the LDM refinement ligand is identified with a vertical dashed line. This vertical line may be masked by other curves if the ligand is very highly ranked. The ROC curve figure inset shows NSQ_AUC values for each binding pocket. Finally, a G) bar chart is used to visualise the EF for representative known ligand chemotypes at EF1, EF5 and EF10. Chemotypes A ‘ERG-like’, B, C, D and E represent only a subset of 5-HT1B agonist ligands (S2 Fig). The EF bar chart inset shows the number of ligands for each chemotype cluster between parenthesis. X-ray structure chemotype EF shown in black bars, with the LDM models coloured based on their relative clusters identified in A.
(TIF)

S12 Fig Self refinement LDM experiment on the AA2AR 2YDV-NEC, using NEC as a refinement ligand. A) Dendrogram of the top 25 LDM models and X-ray structure(s), a cutoff line identifies different LDM clusters and their representative LDM models are designated by a colored dot. Representative LDM models are the highest scoring within the cluster based on the OPUS-ICM metric. B) Comparison of binding pocket conformation between the top 25 LDM models and X-ray structure(s). LDM models are colored based on their IFP Jaccard distance with the destination X-ray structure. C) Binding poses of the representative LDM model(s) and the destination X-ray structure. D) IFP of the representative LDM models and the X-ray structure. Interaction type is described for each residue of the binding pocket: hydrophobic interaction, hydrogen bond (H-bond) donor and acceptor, weak hydrogen bond (weak H-bond) donor and acceptor, ionic bond positive (+) and negative (-) and aromatic interaction. VS performance is described with ROC curves to visualise E) the recovery of known ligands vs. decoys and F) the selectivity of inhibitors over agonists (or vice-versa). The relative rank of the LDM refinement ligand is identified with a vertical dashed line. This vertical line may be masked by other curves if the ligand is very highly ranked. The ROC curve figure inset shows NSQ_AUC values for each binding pocket. Finally, a G) bar chart is used to visualise the EF for representative known ligand chemotypes at EF1, EF5 and EF10. Chemotypes A ‘UK-like’, B ‘ADE-like’ and C ‘NEC-like’ represent only a subset of AA2AR agonist ligands (S2 Fig). The EF bar chart inset shows the number of ligands for each chemotype cluster between parenthesis. X-ray structure chemotype EF shown in black bars, with the LDM models coloured based on their relative clusters identified in A.
(TIF)

S13 Fig Self refinement LDM experiment on the CCR5 4MBS-MRV, using MRV as a refinement ligand. A) Dendrogram of the top 25 LDM models and X-ray structure(s), a cutoff line identifies different LDM clusters and their representative LDM models are designated by a colored dot. Representative LDM models are the highest scoring within the cluster based on the OPUS-ICM metric. B) Comparison of binding pocket conformation between the top 25 LDM models and X-ray structure(s). LDM models are colored based on their IFP Jaccard distance with the destination X-ray structure. C) Binding poses of the representative LDM model(s) and the destination X-ray structure. D) IFP of the representative LDM models and the X-ray structure. Interaction type is described for each residue of the binding pocket: hydrophobic interaction, hydrogen bond (H-bond) donor and acceptor, weak hydrogen bond (weak H-bond) donor and acceptor, ionic bond positive (+) and negative (-) and aromatic interaction. VS performance is described with ROC curves to visualise E) the recovery of known ligands vs. decoys and F) the selectivity of inhibitors over agonists (or vice-versa). The relative rank of the LDM refinement ligand is identified with a vertical dashed line. This vertical line may be masked by other curves if the ligand is very highly ranked. The ROC curve figure inset shows NSQ_AUC values for each binding pocket. Finally, a G) bar chart is used to visualise the EF for representative known ligand chemotypes at EF1, EF5 and EF10. Chemotypes A and B ‘MRV-like’ represent only a subset of CCR5 inhibitor ligands (S2 Fig). The EF bar chart inset shows the number of ligands for each chemotype cluster between parenthesis. X-ray structure chemotype EF shown in black bars, with the LDM models coloured based on their relative clusters identified in A.
(TIF)

S14 Fig Self refinement LDM experiment on the DOR 4N6H-NAL, using NAL as a refinement ligand, using the 3D ligand library. A) Dendrogram of the top 25 LDM models and X-ray structure(s), a cutoff line identifies different LDM clusters and their representative LDM models are designated by a colored dot. Representative LDM models are the highest scoring within the cluster based on the OPUS-ICM metric. B) Comparison of binding pocket conformation between the top 25 LDM models and X-ray structure(s). LDM models are colored based on their IFP Jaccard distance with the destination X-ray structure. C) Binding poses of the representative LDM model(s) and the destination X-ray structure. D) IFP of the representative LDM models and the X-ray structure. Interaction type is described for each residue of the binding pocket: hydrophobic interaction, hydrogen bond (H-bond) donor and acceptor, weak hydrogen bond (weak H-bond) donor and acceptor, ionic bond positive (+) and negative (-) and aromatic interaction. VS performance is described with ROC curves to visualise E) the recovery of known ligands vs. decoys and F) the selectivity of inhibitors over agonists (or vice-versa). The relative rank of the LDM refinement ligand is identified with a vertical dashed line. This vertical line may be masked by other curves if the ligand is very highly ranked. The ROC curve figure inset shows NSQ_AUC values for each binding pocket. Finally, a G) bar chart is used to visualise the EF for representative known ligand chemotypes at EF1, EF5 and EF10. Chemotypes A ‘NAL-like’ and B represent only a subset of DOR inhibitor ligands (S2 Fig). The EF bar chart inset shows the number of ligands for each chemotype cluster between parenthesis. X-ray structure chemotype EF shown in black bars, with the LDM models coloured based on their relative clusters identified in A.
(TIF)

S15 Fig Chemotype switch LDM experiment on the B2AR 3NY9-KOL to 2RH1-CAR, using CAR as a refinement ligand. A) Dendrogram of the top 25 LDM models and X-ray structure(s), a cutoff line identifies different LDM clusters and their representative LDM models are designated by a colored dot. Representative LDM models are the highest scoring within the cluster based on the OPUS-ICM metric. B) Comparison of binding pocket conformation between the top 25 LDM models and X-ray structure(s). LDM models are colored based on their IFP Jaccard distance with the destination X-ray structure. C) Binding poses of the representative LDM model(s) and the destination X-ray structure. D) IFP of the representative LDM models and the X-ray structures. Interaction type is described for each residue of the binding pocket: hydrophobic interaction, hydrogen bond (H-bond) donor and acceptor, weak hydrogen bond (weak H-bond) donor and acceptor, ionic bond positive (+) and negative (-) and aromatic interaction. VS performance is described with ROC curves to visualise E) the recovery of known ligands vs. decoys and F) the selectivity of inhibitors over agonists (or vice-versa). The relative rank of the LDM refinement ligand is identified with a vertical dashed line. This vertical line may be masked by other curves if the ligand is very highly ranked. The ROC curve figure inset shows NSQ_AUC values for each binding pocket. Finally, a G) bar chart is used to visualise the EF for representative known ligand chemotypes at EF1, EF5 and EF10. Chemotypes A ‘ICI-like’, B ‘KOL-like’, C ‘TIM-like’, D ‘CAR-like’ and E represent only a subset of B2AR inhibitor ligands (S2 Fig). The EF bar chart inset shows the number of ligands for each chemotype cluster between parenthesis. Origin and destination X-ray structure chemotype EF shown in grey and black bars, respectively, with the LDM models coloured based on their relative clusters identified in A.
(TIF)

S16 Fig Chemotype switch LDM experiment on the AA2AR 3QAK-UK to 2YDV-NEC, using NEC as a refinement ligand. A) Dendrogram of the top 25 LDM models and X-ray structure(s), a cutoff line identifies different LDM clusters and their representative LDM models are designated by a colored dot. Representative LDM models are the highest scoring within the cluster based on the OPUS-ICM metric. B) Comparison of binding pocket conformation between the top 25 LDM models and X-ray structure(s). LDM models are colored based on their IFP Jaccard distance with the destination X-ray structure. C) Binding poses of the representative LDM model(s) and the destination X-ray structure. D) IFP of the representative LDM models and the X-ray structures. Interaction type is described for each residue of the binding pocket: hydrophobic interaction, hydrogen bond (H-bond) donor and acceptor, weak hydrogen bond (weak H-bond) donor and acceptor, ionic bond positive (+) and negative (-) and aromatic interaction. VS performance is described with ROC curves to visualise E) the recovery of known ligands vs. decoys and F) the selectivity of inhibitors over agonists (or vice-versa). The relative rank of the LDM refinement ligand is identified with a vertical dashed line. This vertical line may be masked by other curves if the ligand is very highly ranked. The ROC curve figure inset shows NSQ_AUC values for each binding pocket. Finally, a G) bar chart is used to visualise the EF for representative known ligand chemotypes at EF1, EF5 and EF10. Chemotypes A ‘UK-like’, B ‘ADE-like’ and C ‘NEC-like’ represent only a subset of AA2AR agonist ligands (S2 Fig). The EF bar chart inset shows the number of ligands for each chemotype cluster between parenthesis. Origin and destination X-ray structure chemotype EF shown in grey and black bars, respectively, with the LDM models coloured based on their relative clusters identified in A.
(TIF)

S17 Fig Chemotype switch LDM experiment on the B2AR 3D4S-TIM to 3NY8-ICI, using ICI as a refinement ligand. A) Dendrogram of the top 25 LDM models and X-ray structure(s), a cutoff line identifies different LDM clusters and their representative LDM models are designated by a colored dot. Representative LDM models are the highest scoring within the cluster based on the OPUS-ICM metric. B) Comparison of binding pocket conformation between the top 25 LDM models and X-ray structure(s). LDM models are colored based on their IFP Jaccard distance with the destination X-ray structure. C) Binding poses of the representative LDM model(s) and the destination X-ray structure. D) IFP of the representative LDM models and the X-ray structures. Interaction type is described for each residue of the binding pocket: hydrophobic interaction, hydrogen bond (H-bond) donor and acceptor, weak hydrogen bond (weak H-bond) donor and acceptor, ionic bond positive (+) and negative (-) and aromatic interaction. VS performance is described with ROC curves to visualise E) the recovery of known ligands vs. decoys and F) the selectivity of inhibitors over agonists (or vice-versa). The relative rank of the LDM refinement ligand is identified with a vertical dashed line. This vertical line may be masked by other curves if the ligand is very highly ranked. The ROC curve figure inset shows NSQ_AUC values for each binding pocket. Finally, a G) bar chart is used to visualise the EF for representative known ligand chemotypes at EF1, EF5 and EF10. Chemotypes A ‘ICI-like’, B ‘KOL-like’, C ‘TIM-like’, D ‘CAR-like’ and E represent only a subset of B2AR inhibitor ligands (S2 Fig). The EF bar chart inset shows the number of ligands for each chemotype cluster between parenthesis. Origin and destination X-ray structure chemotype EF shown in grey and black bars, respectively, with the LDM models coloured based on their relative clusters identified in A.
(TIF)

S18 Fig Chemotype switch LDM experiment on the B2AR 3D4S-TIM to 3NY8-ICI, using ICI as a refinement ligand, all LDM models from the best cluster. A) Dendrogram of the top 25 LDM models and X-ray structure(s), a cutoff line identifies different LDM clusters and their representative LDM models are designated by a colored dot. Representative LDM models are the highest scoring within the cluster based on the OPUS-ICM metric. B) Comparison of binding pocket conformation between the top 25 LDM models and X-ray structure(s). LDM models are colored based on their IFP Jaccard distance with the destination X-ray structure. C) Binding poses of the representative LDM model(s) and the destination X-ray structure. D) IFP of the representative LDM models and the X-ray structures. Interaction type is described for each residue of the binding pocket: hydrophobic interaction, hydrogen bond (H-bond) donor and acceptor, weak hydrogen bond (weak H-bond) donor and acceptor, ionic bond positive (+) and negative (-) and aromatic interaction. VS performance is described with ROC curves to visualise E) the recovery of known ligands vs. decoys and F) the selectivity of inhibitors over agonists (or vice-versa). The relative rank of the LDM refinement ligand is identified with a vertical dashed line. This vertical line may be masked by other curves if the ligand is very highly ranked. The ROC curve figure inset shows NSQ_AUC values for each binding pocket. Finally, a G) bar chart is used to visualise the EF for representative known ligand chemotypes at EF1, EF5 and EF10. Chemotypes A ‘ICI-like’, B ‘KOL-like’, C ‘TIM-like’, D ‘CAR-like’ and E represent only a subset of B2AR inhibitor ligands (S2 Fig). The EF bar chart inset shows the number of ligands for each chemotype cluster between parenthesis. Origin and destination X-ray structure chemotype EF shown in grey and black bars, respectively, with the LDM models coloured based on their relative clusters identified in A.
(TIF)

S19 Fig Chemotype switch LDM experiment on the AA2AR 3UZA-T4G to 3REY-XAC, using XAC as a refinement ligand. A) Dendrogram of the top 25 LDM models and X-ray structure(s), a cutoff line identifies different LDM clusters and their representative LDM models are designated by a colored dot. Representative LDM models are the highest scoring within the cluster based on the OPUS-ICM metric. B) Comparison of binding pocket conformation between the top 25 LDM models and X-ray structure(s). LDM models are colored based on their IFP Jaccard distance with the destination X-ray structure. C) Binding poses of the representative LDM model(s) and the destination X-ray structure. D) IFP of the representative LDM models and the X-ray structures. Interaction type is described for each residue of the binding pocket: hydrophobic interaction, hydrogen bond (H-bond) donor and acceptor, weak hydrogen bond (weak H-bond) donor and acceptor, ionic bond positive (+) and negative (-) and aromatic interaction. VS performance is described with ROC curves to visualise E) the recovery of known ligands vs. decoys and F) the selectivity of inhibitors over agonists (or vice-versa). The relative rank of the LDM refinement ligand is identified with a vertical dashed line. This vertical line may be masked by other curves if the ligand is very highly ranked. The ROC curve figure inset shows NSQ_AUC values for each binding pocket. Finally, a G) bar chart is used to visualise the EF for representative known ligand chemotypes at EF1, EF5 and EF10. Chemotypes A ‘ZM-like’, B ‘T4G-like’, C ‘XAC-like’ and D ‘CAF-like’ represent only a subset of AA2AR inhibitors ligands (S2 Fig). The EF bar chart inset shows the number of ligands for each chemotype cluster between parenthesis. Origin and destination X-ray structure chemotype EF shown in grey and black bars, respectively, with the LDM models coloured based on their relative clusters identified in A.
(TIF)

S20 Fig Pharmacology switch LDM experiment on the M2R 3UON-QNB to 4MQS-IXO, using IXO as a refinement ligand. A) Dendrogram of the top 25 LDM models and X-ray structure(s), a cutoff line identifies different LDM clusters and their representative LDM models are designated by a colored dot. Representative LDM models are the highest scoring within the cluster based on the OPUS-ICM metric. B) Comparison of binding pocket conformation between the top 25 LDM models and X-ray structure(s). LDM models are colored based on their IFP Jaccard distance with the destination X-ray structure. C) Binding poses of the representative LDM model(s) and the destination X-ray structure. D) IFP of the representative LDM models and the X-ray structures. Interaction type is described for each residue of the binding pocket: hydrophobic interaction, hydrogen bond (H-bond) donor and acceptor, weak hydrogen bond (weak H-bond) donor and acceptor, ionic bond positive (+) and negative (-) and aromatic interaction. VS performance is described with ROC curves to visualise E) the recovery of known ligands vs. decoys and F) the selectivity of inhibitors over agonists (or vice-versa). The relative rank of the LDM refinement ligand is identified with a vertical dashed line. This vertical line may be masked by other curves if the ligand is very highly ranked. The ROC curve figure inset shows NSQ_AUC values for each binding pocket. Finally, a G) bar chart is used to visualise the EF for representative known ligand chemotypes at EF1, EF5 and EF10. Chemotypes A, B and C ‘IXO-like’ represent only a subset of M2R agonist ligands (S2 Fig). The EF bar chart inset shows the number of ligands for each chemotype cluster between parenthesis. Origin and destination X-ray structure chemotype EF shown in grey and black bars, respectively, with the LDM models coloured based on their relative clusters identified in A.
(TIF)

S21 Fig Pharmacology switch LDM experiment on the M2R 4MQS-IXO to 3UON-QNB, using QNB as a refinement ligand. A) Dendrogram of the top 25 LDM models and X-ray structure(s), a cutoff line identifies different LDM clusters and their representative LDM models are designated by a colored dot. Representative LDM models are the highest scoring within the cluster based on the OPUS-ICM metric. B) Comparison of binding pocket conformation between the top 25 LDM models and X-ray structure(s). LDM models are colored based on their IFP Jaccard distance with the destination X-ray structure. C) Binding poses of the representative LDM model(s) and the destination X-ray structure. D) IFP of the representative LDM models and the X-ray structures. Interaction type is described for each residue of the binding pocket: hydrophobic interaction, hydrogen bond (H-bond) donor and acceptor, weak hydrogen bond (weak H-bond) donor and acceptor, ionic bond positive (+) and negative (-) and aromatic interaction. VS performance is described with ROC curves to visualise E) the recovery of known ligands vs. decoys and F) the selectivity of inhibitors over agonists (or vice-versa). The relative rank of the LDM refinement ligand is identified with a vertical dashed line. This vertical line may be masked by other curves if the ligand is very highly ranked. The ROC curve figure inset shows NSQ_AUC values for each binding pocket. Finally, a G) bar chart is used to visualise the EF for representative known ligand chemotypes at EF1, EF5 and EF10. Chemotypes A, B ‘QNB-like’ and C represent only a subset of M2R inhibitor ligands (S2 Fig). The EF bar chart inset shows the number of ligands for each chemotype cluster between parenthesis. Origin and destination X-ray structure chemotype EF shown in grey and black bars, respectively, with the LDM models coloured based on their relative clusters identified in A.
(TIF)

S22 Fig Pharmacology switch LDM experiment on the AA2AR 3EML-ZM to 2YDV-NEC, using NEC as a refinement ligand. A) Dendrogram of the top 25 LDM models and X-ray structure(s), a cutoff line identifies different LDM clusters and their representative LDM models are designated by a colored dot. Representative LDM models are the highest scoring within the cluster based on the OPUS-ICM metric. B) Comparison of binding pocket conformation between the top 25 LDM models and X-ray structure(s). LDM models are colored based on their IFP Jaccard distance with the destination X-ray structure. C) Binding poses of the representative LDM model(s) and the destination X-ray structure. D) IFP of the representative LDM models and the X-ray structures. Interaction type is described for each residue of the binding pocket: hydrophobic interaction, hydrogen bond (H-bond) donor and acceptor, weak hydrogen bond (weak H-bond) donor and acceptor, ionic bond positive (+) and negative (-) and aromatic interaction. VS performance is described with ROC curves to visualise E) the recovery of known ligands vs. decoys and F) the selectivity of inhibitors over agonists (or vice-versa). The relative rank of the LDM refinement ligand is identified with a vertical dashed line. This vertical line may be masked by other curves if the ligand is very highly ranked. The ROC curve figure inset shows NSQ_AUC values for each binding pocket. Finally, a G) bar chart is used to visualise the EF for representative known ligand chemotypes at EF1, EF5 and EF10. Chemotypes A ‘UK-like’, B ‘ADE-like’ and C ‘NEC-like’ represent only a subset of AA2AR agonist ligands (S2 Fig). The EF bar chart inset shows the number of ligands for each chemotype cluster between parenthesis. Origin and destination X-ray structure chemotype EF shown in grey and black bars, respectively, with the LDM models coloured based on their relative clusters identified in A.
(TIF)

S23 Fig Pharmacology switch LDM experiment on the AA2AR 3QAK-UK to 3EML-ZM, using ZM as a refinement ligand. A) Dendrogram of the top 25 LDM models and X-ray structure(s), a cutoff line identifies different LDM clusters and their representative LDM models are designated by a colored dot. Representative LDM models are the highest scoring within the cluster based on the OPUS-ICM metric. B) Comparison of binding pocket conformation between the top 25 LDM models and X-ray structure(s). LDM models are colored based on their IFP Jaccard distance with the destination X-ray structure. C) Binding poses of the representative LDM model(s) and the destination X-ray structure. D) IFP of the representative LDM models and the X-ray structures. Interaction type is described for each residue of the binding pocket: hydrophobic interaction, hydrogen bond (H-bond) donor and acceptor, weak hydrogen bond (weak H-bond) donor and acceptor, ionic bond positive (+) and negative (-) and aromatic interaction. VS performance is described with ROC curves to visualise E) the recovery of known ligands vs. decoys and F) the selectivity of inhibitors over agonists (or vice-versa). The relative rank of the LDM refinement ligand is identified with a vertical dashed line. This vertical line may be masked by other curves if the ligand is very highly ranked. The ROC curve figure inset shows NSQ_AUC values for each binding pocket. Finally, a G) bar chart is used to visualise the EF for representative known ligand chemotypes at EF1, EF5 and EF10. Chemotypes A ‘ZM-like’, B ‘T4G-like’, C ‘XAC-like’ and D ‘CAF-like’ represent only a subset of AA2AR inhibitors ligands (S2 Fig). The EF bar chart inset shows the number of ligands for each chemotype cluster between parenthesis. Origin and destination X-ray structure chemotype EF shown in grey and black bars, respectively, with the LDM models coloured based on their relative clusters identified in A.
(TIF)
