# Supplementary material for: Improving virtual screening of G protein-coupled receptors via ligand-directed modeling
Source: PLoS Comput Biol. 2017 Nov 13;13(11):e1005819. doi: 10.1371/journal.pcbi.1005819 (PMC5708846; doi:10.1371/journal.pcbi.1005819)
Supplement: S1 Table — Description of the method used at each step of the LDM workflow. Parameters used in the current study for each of these methods. CONCOORD parameters that were common in both step where the software is used: secondary conformation detection: DSSP, forcefield: OPLS-UA (united atom), bond and angle parameters: CONCOORD default. (PDF) [file pcbi.1005819.s001.pdf]

Step	Method	Parameters
● Preparation		
○ Binding pocket	Run ICMPocketFinder, select residues around pocket	1.5 Å radius around pocket.
○ Cytoplasmic residues	Define cytoplasmic domain by selecting Ballosteros-Weinstein (BW) residues	BW cytoplasmic residues: 1.48, 2.51, 3.38, 4.51, 5.50, 6.43, 7.45
● Independent replica		80 replicas
○ Initialise		
■ Minimise	GROMACS with explicit waters	FFGMX forcefield, water model SPC, Electrostatics: PME and epsilon_r=1, default pdb2gmx residue charge, BFGS method, 5000 steps.
■ Starting structure	CONCOORD generate conformation	x1 conformation. Use input as reference. 10000 regularisation steps.
○ Iterative rounds		8 iterative rounds
■ Directory		20 directories
● Sample receptor main chain	CONCOORD generate conformations	x30 conformations. Minimum 200 distance definition for each atom. Fix cytoplasmic residues. Use random seed.
● Select receptor	Select conformations under a maximum RMSD from the input	< 1.2 Å RMSD.
● Sample binding pocket side chains	tCONCOORD generate conformations	x5 conformations. Sample binding pocket side chains only.
● Select binding pocket	Select conformations that conserved % of binding pocket polar residues from input	8/10 polar residues.
● Rebuild	Add cytoplasm side chains with scoomp	Consider ligand in energy calculations
● Minimise	GROMACS with explicit waters	(Identical to minimisation above)
● Dock	ICM docking, binding pocket refinement	Flexible ligand docking to pre-calculated energy grid. Docking effort: 5. Select best scored conformation. Refine residues 2 Å around ligand.
● Score	Score docked pose with ICM, receptor with OPUS_PSP	
■ Scoring		
● Select	Best ICM scored from complexes in this directory	
● Final scoring (quantitative and qualitative)	OPUS-ICM rank all complexes and cluster by IFP	Up to 1,920,000 complexes generated and up to 640 LDM complexes ranked by OPUS-ICM. The top 25 LDM complexes are clustered by IFP. Analyse best scoring LDM complex from each cluster at cutoff ~0.7 Jaccard distance.

References

1. Kabsch W, Sander C. Dictionary of protein secondary structure: pattern recognition of hydrogen-bonded and geometrical features. Biopolymers. 1983;22: 2577–2637.
2. Jorgensen WL, Tirado-Rives J. The OPLS [optimized potentials for liquid simulations] potential functions for proteins, energy minimizations for crystals of cyclic peptides and crambin. J Am Chem Soc. 1988;110: 1657–1666.
