# Supplementary material for: Improving virtual screening of G protein-coupled receptors via ligand-directed modeling
Source: PLoS Comput Biol. 2017 Nov 13;13(11):e1005819. doi: 10.1371/journal.pcbi.1005819 (PMC5708846; doi:10.1371/journal.pcbi.1005819)
Supplement: S2 Table — Details about ligand name and structure as well as mutations in the TM bundle and their proximity to the X-ray ligand. (PDF) [file pcbi.1005819.s002.pdf]

GPCR (abbreviation and full name)	Bound ligand pharmacology	PDB code	Resolution (Å)	Stabilization					Reference
				**Ligand** (abbreviation and full name)	**Ligand structure**	**Mutations in the TM and ECL2-distal domain** (Residues within a 4 Å radius of the bound X-ray ligand are marked in bold)	**Insertion** (insertion protein [1] and location)	**Other protein**	
AA2AR (adenosine A2a receptor)	agonist	2YDV	2.6	NEC (NECA)	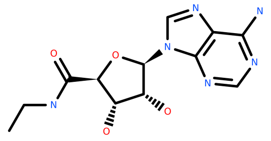	L48A, A54L, T65A, **Q89A**	-	-	Lebon et al. 2011 [2]
		3QAK	2.7	UK (UK-432,097)	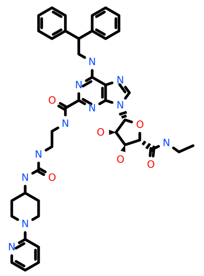	-	T4L (ICL3)	-	Xu et al. 2011 [3]

	inhibitor	3PWH	3.3	ZM (ZM-241,385)	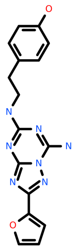	A54L, T88A, K122A, L202A, L235A, V239A, S277A	-	-	Doré et al. 2011 [4]
		4EIY	1.8	ZM (ZM-241,385)	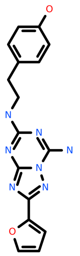	-	BRIL (ICL3)	-	Liu et al. 2012 [5]
		3EML	2.6	ZM (ZM-241,385)	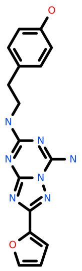	- (mutations introduced for LDM experiment 3EML-ZM to 2YDV-NEC: L48A, A54L, T65A, Q89A)	T4L (ICL3)	-	Jaakola et al. 2008 [6]

		3RFM	3.6	CAF (caffeine)	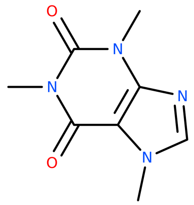	A54L, T88A, K122A, L202A, L235A, V239A, S277A	-	-	Doré et al. 2011 [4]
		3UZA	3.3	T4G (1,2,4-triazine 4g)	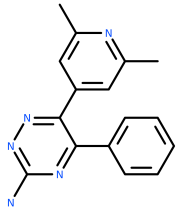	A54L, T88A, K122A, L202A, L235A, V239A, **S277A**	-	-	Congreve et al. 2012 [7]
		3REY	3.3	XAC (xanthine amine congener)	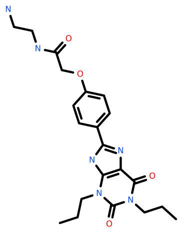	A54L, T88A, K122A, L202A, L235A, V239A, S277A	-	-	Doré et al. 2011 [4]
B2AR (beta-2 adrenergic receptor)	agonist	3P0G	3.5	BI (BI-167,107)	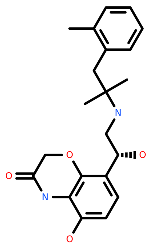	-	-	Nanobody	Rasmussen et al. 2011 [8]

		4LDE	2.8	BI (BI-167,107)	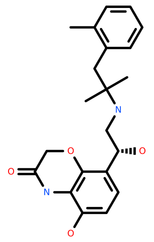	-	T4L (N-term)	Nanobody	Ring et al. 2013 [9]
		4LDO	3.2	ADR (adrenaline)	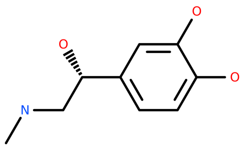	-	T4L (N-term)	Nanobody	Ring et al. 2013 [9]
		4LDL	3.1	ISO (hydroxybenzyl isoproterenol)	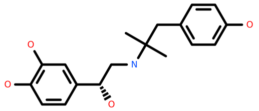	-	T4L (N-term)	Nanobody	Ring et al. 2013 [9]

	inhibitor	2RH1	2.4	CAR (carazolol)	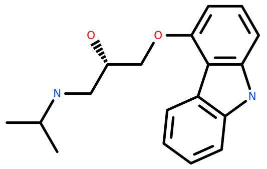	-	T4L (ICL3)	-	Cherezov et al. 2007 [10]
		3D4S	2.8	TIM (timolol)	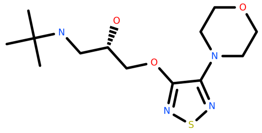	E122W	T4L (ICL3)	-	Hanson et al. 2008 [11]
		3NY8	2.8	ICI (ICI-118,551)	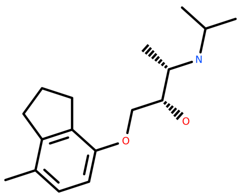	E122W	T4L (ICL3)	-	Wacker et al. 2010 [12]

		3NY9	2.8	KOL (compound 2 from Kolb et al. 2009)	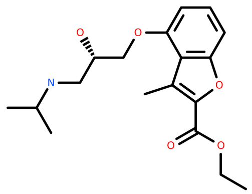	E122W	T4L (ICL3)	-	Wacker et al. 2010 [12]
M2R (muscarinic acetylcholine receptor M2)	agonist	4MQS	3.5	IXO (iperoxo)	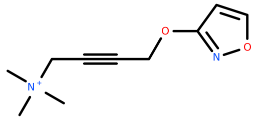	-	-	Nanobody	Kruse et al. 2013 [13]
	inhibitor	3UON	3.0	QNB (3-quinuclidinyl-benzilate)	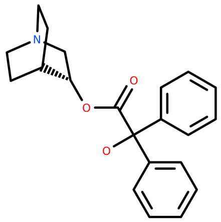	-	T4L (ICL3)	-	Haga et al. 2012 [14]

CCR5 (C-C chemokine receptor type 5)	inhibitor	4MBS		MRV (maraviroc)	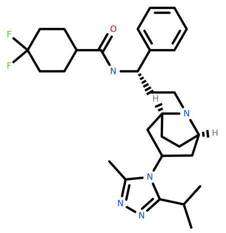	G163N, A233D, K303E	Rubredoxin (ICL3)	-	Tan et al. 2013 [15]
H1R (histamine H1 receptor)	inhibitor	3RZE		DOX (doxepin)	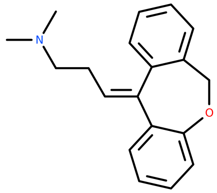	-	T4L (ICL3)	-	Shimamura et al. 2011 [16]
DOR (delta opioid receptor)	inhibitor	4N6H		NAL (naltrindole)	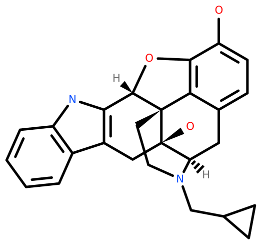	-	BRIL (N-term)	-	Fenalti et al. 2014 [17]

5-HT1B (5-hydroxytryptamine receptor 1B)	inhibitor	4IAR		ERG (ergotamine)	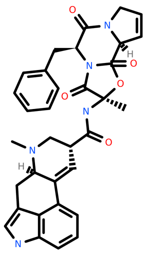	L138W	BRIL (ICL3)	-	Wang et al. 2013 [18]

References

1. Chun E, Thompson AA, Liu W, Roth CB, Griffith MT, Katritch V, et al. Fusion partner toolchest for the stabilization and crystallization of G protein-coupled receptors. Structure. Elsevier Ltd; 2012;20: 967–976.

2. Lebon G, Warne T, Edwards PC, Bennett K, Langmead CJ, Leslie AGW, et al. Agonist-bound adenosine A2A receptor structures reveal common features of GPCR activation. Nature. Nature Publishing Group; 2011;474: 521–525.

3. Xu F, Wu H, Katritch V, Han GW, Jacobson KA, Gao Z-G, et al. Structure of an agonist-bound human A2A adenosine receptor. Science. American Association for the Advancement of Science; 2011;332: 322–327.

4. Doré AS, Robertson N, Errey JC, Ng I, Hollenstein K, Tehan B, et al. Structure of the adenosine A(2A) receptor in complex with ZM241385 and the xanthines XAC and caffeine. Structure. 2011;19: 1283–1293.

5. Liu W, Chun E, Thompson AA, Chubukov P, Xu F, Katritch V, et al. Structural basis for allosteric regulation of GPCRs by sodium ions. Science. 2012;337: 232–236.

6. Jaakola V-P, Griffith MT, Hanson MA, Cherezov V, Chien EYT, Lane JR, et al. The 2.6 angstrom crystal structure of a human A2A adenosine receptor bound to an antagonist. Science. 2008;322: 1211–1217.

7. Congreve M, Andrews SP, Doré AS, Hollenstein K, Hurrell E, Langmead CJ, et al. Discovery of 1,2,4-Triazine Derivatives as Adenosine A2A Antagonists using Structure Based Drug Design. J Med Chem. 2012;55: 1898–1903.

8. Rasmussen SGF, Choi H-J, Fung JJ, Pardon E, Casarosa P, Chae PS, et al. Structure of a nanobody-stabilized active state of

the β(2) adrenoceptor. Nature. Nature Publishing Group; 2011;469: 175–180.

9. Ring AM, Manglik A, Kruse AC, Enos MD, Weis WI, Garcia KC, et al. Adrenaline-activated structure of β2-adrenoceptor stabilized by an engineered nanobody. Nature. Nature Publishing Group; 2013;502: 575–579.

10. Cherezov V, Rosenbaum DM, Hanson MA, Rasmussen SGF, Thian FS, Kobilka TS, et al. High-resolution crystal structure of an engineered human beta2-adrenergic G protein-coupled receptor. Science. 2007;318: 1258–1265.

11. Hanson MA, Cherezov V, Griffith MT, Roth CB, Jaakola V-P, Chien EYT, et al. A specific cholesterol binding site is established by the 2.8 A structure of the human beta2-adrenergic receptor. Structure. 2008;16: 897–905.

12. Wacker D, Fenalti G, Brown MA, Katritch V, Abagyan R, Cherezov V, et al. Conserved binding mode of human beta2 adrenergic receptor inverse agonists and antagonist revealed by X-ray crystallography. J Am Chem Soc. 2010;132: 11443–11445.

13. Kruse AC, Ring AM, Manglik A, Hu J, Hu K, Eitel K, et al. Activation and allosteric modulation of a muscarinic acetylcholine receptor. Nature. 2013;504: 101–106.

14. Haga K, Kruse AC, Asada H, Yurugi-Kobayashi T, Shiroishi M, Zhang C, et al. Structure of the human M2 muscarinic acetylcholine receptor bound to an antagonist. Nature. Nature Publishing Group; 2012;482: 547–551.

15. Tan Q, Zhu Y, Li J, Chen Z, Han GW, Kufareva I. Structure of the CCR5 Chemokine receptor–HIV entry inhibitor maraviroc complex. Science. 2013;341: 1387–1390.

16. Shimamura T, Shiroishi M, Weyand S, Tsujimoto H, Winter G, Katritch V, et al. Structure of the human histamine H1 receptor complex with doxepin. Nature. Nature Publishing Group; 2011;475: 65–70.

17. Fenalti G, Giguere PM, Katritch V, Huang X-P, Thompson AA, Cherezov V, et al. Molecular control of δ-opioid receptor signalling. Nature. Nature Publishing Group; 2014;506: 191–196.

18. Wang C, Jiang Y, Ma J, Wu H, Wacker D, Katritch V, et al. Structural basis for molecular recognition at serotonin receptors. Science. 2013;340: 610–614.
