# Supplementary material for: Improving virtual screening of G protein-coupled receptors via ligand-directed modeling
Source: PLoS Comput Biol. 2017 Nov 13;13(11):e1005819. doi: 10.1371/journal.pcbi.1005819 (PMC5708846; doi:10.1371/journal.pcbi.1005819)
Supplement: S3 Table — Selected LDM models and X-ray origin and destination structures are compared by ligand heavy atom RMSD to the destination X-ray structure and VS performance by recovery NSQ_AUC and selectivity NSQ_AUC values. (PDF) [file pcbi.1005819.s003.pdf]

Scenario	GPCR	LDM experiment	LDM model	RMSD to destination X-ray structure on ligand heavy atoms (Å)	NSQ_AUC recovery	NSQ_AUC selectivity	LDM experiment figure
self refinement	AA2AR	2YDV-NEC	2YDV-NEC	0.0	48.2	54.6	S12 Fig
self refinement	AA2AR	2YDV-NEC	LDM 000	0.77	9.6	9.8	S12 Fig
self refinement	AA2AR	2YDV-NEC	LDM 005	0.53	14.7	3.3	S12 Fig
self refinement	AA2AR	2YDV-NEC	LDM 008	0.89	17.8	14.9	S12 Fig
self refinement	AA2AR	3PWH-ZM	3PWH-ZM	0.0	29.1	53.3	S7 Fig
self refinement	AA2AR	3PWH-ZM	LDM 000	3.33	12.7	-6.2	S7 Fig
self refinement	AA2AR	3PWH-ZM	LDM 001	1.36	16.2	-4.0	S7 Fig
self refinement	AA2AR	3PWH-ZM	LDM 006	2.07	12.5	22.3	S7 Fig
self refinement	AA2AR	3PWH-ZM	LDM 010	5.43	30.5	23.1	S7 Fig
self refinement	AA2AR	4EIY-ZM	4EIY-ZM	0.0	35.1	5.1	S6 Fig
self refinement	AA2AR	4EIY-ZM	LDM 000	9.34	17.2	6.0	S6 Fig
self refinement	AA2AR	4EIY-ZM	LDM 004	9.25	11.2	-4.0	S6 Fig
self refinement	AA2AR	4EIY-ZM	LDM 008	3.35	34.8	20.4	S6 Fig
self refinement	AA2AR	4EIY-ZM	LDM 011	5.45	-1.0	17.9	S6 Fig
self refinement	AA2AR	4EIY-ZM	LDM 013	3.33	31.2	15.3	S6 Fig
self refinement	AA2AR	4EIY-ZM	LDM 015	3.48	9.5	18.4	S6 Fig
self refinement	B2AR	2RH1-CAR	2RH1-CAR	0.0	59.1	32.8	Fig 4
self refinement	B2AR	2RH1-CAR	LDM 000	0.70	26.4	-5.2	Fig 4
self refinement	B2AR	2RH1-CAR	LDM 004	0.66	33.6	3.7	Fig 4
self refinement	B2AR	2RH1-CAR	LDM 008	0.70	26.6	-8.4	Fig 4
self refinement	B2AR	3P0G-BI	3P0G-BI	0.0	42.0	33.1	S5 Fig
self refinement	B2AR	3P0G-BI	LDM 000	1.71	73.9	68.7	S5 Fig
self refinement	B2AR	3P0G-BI	LDM 021	1.75	50.9	32.8	S5 Fig
self refinement	B2AR	4LDE-BI	4LDE-BI	0.0	62.3	44.1	Fig 3
self refinement	B2AR	4LDE-BI	LDM 000	1.58	71.6	59.1	Fig 3
self refinement	B2AR	4LDE-BI	LDM 021	1.56	80.0	64.5	Fig 3
self refinement	CCR5	4MBS-MRV	4MBS-MRV	0.0	59.5	-	S13 Fig
self refinement	CCR5	4MBS-MRV	LDM 000	3.68	20.4	-	S13 Fig
self refinement	CCR5	4MBS-MRV	LDM 003	6.42	8.5	-	S13 Fig
self refinement	CCR5	4MBS-MRV	LDM 004	10.42	58.1	-	S13 Fig
self refinement	CCR5	4MBS-MRV	LDM 008	9.88	6.5	-	S13 Fig
self refinement	CCR5	4MBS-MRV	LDM 009	8.62	-13.5	-	S13 Fig

self refinement	CCR5	4MBS-MRV	LDM 012	6.32	-8.4	-	S13 Fig
self refinement	CCR5	4MBS-MRV	LDM 013	1.61	12.0	-	S13 Fig
self refinement	CCR5	4MBS-MRV	LDM 015	8.30	8.3	-	S13 Fig
self refinement	CCR5	4MBS-MRV	LDM 019	1.61	-11.1	-	S13 Fig
self refinement	H1R	3RZE-DOX	3RZE-DOX	0.0	48.1	14.9	S10 Fig
self refinement	H1R	3RZE-DOX	LDM 000	0.96	43.9	38.9	S10 Fig
self refinement	H1R	3RZE-DOX	LDM 001	5.42	17.8	10.5	S10 Fig
self refinement	H1R	3RZE-DOX	LDM 004	0.96	32.4	38.8	S10 Fig
self refinement	H1R	3RZE-DOX	LDM 007	1.15	38.0	30.7	S10 Fig
self refinement	H1R	3RZE-DOX	LDM 010	2.37	7.4	55.9	S10 Fig
self refinement	H1R	3RZE-DOX	LDM 018	5.57	19.2	38.9	S10 Fig
self refinement	M2R	3UON-QNB	3UON-QNB	0.0	16.3	6.9	S8 Fig
self refinement	M2R	3UON-QNB	LDM 000	2.12	-3.5	0.2	S8 Fig
self refinement	M2R	3UON-QNB	LDM 001	2.95	9.7	36.2	S8 Fig
self refinement	M2R	4MQS-IXO	4MQS-IXO	0.0	41.0	87.2	S9 Fig
self refinement	M2R	4MQS-IXO	LDM 000	1.50	44.7	91.2	S9 Fig
self refinement	M2R	4MQS-IXO	LDM 009	1.23	53.0	85.3	S9 Fig
self refinement	M2R	4MQS-IXO	LDM 022	6.00	2.1	77.2	S9 Fig
self refinement	DOR	4N6H-NAL	4N6H-NAL	0.0	-19.1	-17	S14 Fig
self refinement	DOR	4N6H-NAL	LDM 000	8.16	-30.2	-26.2	S14 Fig
self refinement	DOR	4N6H-NAL	LDM 001	8.17	-31.8	-32.2	S14 Fig
self refinement	DOR	4N6H-NAL	LDM 003	3.88	-26.9	-33.6	S14 Fig
self refinement	DOR	4N6H-NAL	LDM 008	8.41	-20.0	-16.6	S14 Fig
self refinement	DOR	4N6H-NAL	LDM 014	4.25	-13.7	-13.6	S14 Fig
self refinement	5HT1B	4IAR-ERG	4IAR-ERG	0.0	25.5	15.7	S11 Fig
self refinement	5HT1B	4IAR-ERG	LDM 000	1.85	10.4	4.7	S11 Fig
self refinement	5HT1B	4IAR-ERG	LDM 001	5.83	-0.6	-7.1	S11 Fig
self refinement	5HT1B	4IAR-ERG	LDM 002	1.52	20.0	10.7	S11 Fig
self refinement	5HT1B	4IAR-ERG	LDM 015	1.96	31.6	18.3	S11 Fig
same pharmacology	AA2AR	3PWH-ZM > 3RFM-CAF	3PWH-ZM	-	29.1	53.3	Fig 6
same pharmacology	AA2AR	3PWH-ZM > 3RFM-CAF	3RFM-CAF	0.0	5.4	-1.3	Fig 6
same pharmacology	AA2AR	3PWH-ZM > 3RFM-CAF	LDM 000	4.29	8.6	42.2	Fig 6
same pharmacology	AA2AR	3PWH-ZM > 3RFM-CAF	LDM 001	3.85	6.5	10.6	Fig 6
same pharmacology	AA2AR	3PWH-ZM > 3RFM-CAF	LDM 002	9.75	-14.8	-3.2	Fig 6
same pharmacology	AA2AR	3PWH-ZM > 3RFM-CAF	LDM 005	4.42	-8.6	26.9	Fig 6
same pharmacology	AA2AR	3PWH-ZM > 3RFM-CAF	LDM 008	7.79	5.0	-0.9	Fig 6
same pharmacology	AA2AR	3QAK-UK > 2YDV-NEC	3QAK-UK	-	34.4	40.7	S14 Fig
same pharmacology	AA2AR	3QAK-UK > 2YDV-NEC	2YDV-NEC	0.0	50.6	55.7	S14 Fig

same pharmacology	AA2AR	3QAK-UK > 2YDV-NEC	LDM 000	0.81	25.0	30.2	S14 Fig
same pharmacology	AA2AR	3QAK-UK > 2YDV-NEC	LDM 001	0.58	12.2	9.5	S14 Fig
same pharmacology	AA2AR	3QAK-UK > 2YDV-NEC	LDM 002	0.97	41.7	46.8	S14 Fig
same pharmacology	AA2AR	3QAK-UK > 2YDV-NEC	LDM 009	0.96	32.0	31.6	S14 Fig
same pharmacology	AA2AR	3UZA-T4G > 3REY-XAC	3UZA-T4G	-	15.4	13.4	S19 Fig
same pharmacology	AA2AR	3UZA-T4G > 3REY-XAC	3REY-XAC	0.0	0.0	-2.4	S19 Fig
same pharmacology	AA2AR	3UZA-T4G > 3REY-XAC	LDM 000	6.18	6.4	-4.6	S19 Fig
same pharmacology	AA2AR	3UZA-T4G > 3REY-XAC	LDM 001	11.16	5.0	21.8	S19 Fig
same pharmacology	AA2AR	3UZA-T4G > 3REY-XAC	LDM 004	12.44	9.8	19.0	S19 Fig
same pharmacology	AA2AR	3UZA-T4G > 3REY-XAC	LDM 008	9.09	4.9	15.2	S19 Fig
same pharmacology	B2AR	3D4S-TIM > 3NY8-ICI	3D4S-TIM	-	43.8	5.1	S17 and S18 Figs
same pharmacology	B2AR	3D4S-TIM > 3NY8-ICI	3NY8-ICI	0.0	50.7	20.5	S17 and S18 Figs
same pharmacology	B2AR	3D4S-TIM > 3NY8-ICI	LDM 000	10.07	8.3	-10.6	S17 and S18 Figs
same pharmacology	B2AR	3D4S-TIM > 3NY8-ICI	LDM 001	9.67	6.6	-10.3	S17 and S18 Figs
same pharmacology	B2AR	3D4S-TIM > 3NY8-ICI	LDM 017	1.21	22.0	-12.0	S17 and S18 Figs
same pharmacology	B2AR	3D4S-TIM > 3NY8-ICI	LDM 019	1.12	27.9	-0.6	S17 and S18 Figs
same pharmacology	B2AR	3D4S-TIM > 3NY8-ICI	LDM 020	1.42	33.3	9.2	S17 and S18 Figs
same pharmacology	B2AR	3D4S-TIM > 3NY8-ICI	LDM 023	1.06	37.1	4.4	S17 and S18 Figs
same pharmacology	B2AR	3D4S-TIM > 3NY8-ICI	LDM 024	8.98	17.0	-25.7	S17 and S18 Figs
same pharmacology	B2AR	3NY9-KOL > 2RH1-CAR	3NY9-KOL	-	14.3	-27.2	S15 Fig
same pharmacology	B2AR	3NY9-KOL > 2RH1-CAR	2RH1-CAR	0.0	61.2	34.0	S15 Fig
same pharmacology	B2AR	3NY9-KOL > 2RH1-CAR	LDM 000	1.45	26.3	10.9	S15 Fig
same pharmacology	B2AR	3NY9-KOL > 2RH1-CAR	LDM 003	0.90	26.7	5.3	S15 Fig
same pharmacology	B2AR	3NY9-KOL > 2RH1-CAR	LDM 004	0.85	24.5	9.5	S15 Fig
same pharmacology	B2AR	4LDO-ADR > 4LDL-ISO	4LDO-ADR	-	47.8	33.6	Fig 5
same pharmacology	B2AR	4LDO-ADR > 4LDL-ISO	4LDL-ISO	0.0	66.2	41.7	Fig 5
same pharmacology	B2AR	4LDO-ADR > 4LDL-ISO	LDM 000	0.92	38.7	25.5	Fig 5
same pharmacology	B2AR	4LDO-ADR > 4LDL-ISO	LDM 006	1.54	42.7	32.6	Fig 5
same pharmacology	B2AR	4LDO-ADR > 4LDL-ISO	LDM 015	8.99	54.6	39.5	Fig 5
same pharmacology	B2AR	4LDO-ADR > 4LDL-ISO	LDM 020	1.69	46.3	40.2	Fig 5
pharmacology switch	AA2AR	3QAK-UK > 3EML-ZM	3QAK-UK	-	9.1	-22.0	S23 Fig
pharmacology switch	AA2AR	3QAK-UK > 3EML-ZM	3EML-ZM	0.0	28.5	7.6	S23 Fig
pharmacology switch	AA2AR	3QAK-UK > 3EML-ZM	LDM 000	9.80	13.9	-6.6	S23 Fig
pharmacology switch	AA2AR	3QAK-UK > 3EML-ZM	LDM 016	9.64	1.1	-20.0	S23 Fig
pharmacology switch	AA2AR	3EML-ZM > 2YDV-NEC	3EML-ZM	-	9.5	-5.7	S22 Fig
pharmacology switch	AA2AR	3EML-ZM > 2YDV-NEC	2YDV-NEC	0.0	51.2	57.3	S22 Fig
pharmacology switch	AA2AR	3EML-ZM > 2YDV-NEC	LDM 000	8.79	14.7	22.4	S22 Fig
pharmacology switch	AA2AR	3EML-ZM > 2YDV-NEC	LDM 002	4.40	-3.1	1.4	S22 Fig

pharmacology switch	AA2AR	3EML-ZM > 2YDV-NEC	LDM 009	4.77	16.7	21.5	S22 Fig
pharmacology switch	B2AR	3P0G-BI > 2RH1-CAR	3P0G-BI	-	0.0	-24.1	Fig 8
pharmacology switch	B2AR	3P0G-BI > 2RH1-CAR	2RH1-CAR	0.0	61.2	34.0	Fig 8
pharmacology switch	B2AR	3P0G-BI > 2RH1-CAR	LDM 000	8.34	4.9	-11.8	Fig 8
pharmacology switch	B2AR	3P0G-BI > 2RH1-CAR	LDM 006	8.75	4.3	-34.5	Fig 8
pharmacology switch	B2AR	3P0G-BI > 2RH1-CAR	LDM 010	8.14	2.4	-18.1	Fig 8
pharmacology switch	B2AR	2RH1-CAR > 3P0G-BI	2RH1-CAR	-	28.0	-24.5	Fig 7
pharmacology switch	B2AR	2RH1-CAR > 3P0G-BI	3P0G-BI	0.0	70.4	57.7	Fig 7
pharmacology switch	B2AR	2RH1-CAR > 3P0G-BI	LDM 000	2.15	70.0	61.9	Fig 7
pharmacology switch	B2AR	2RH1-CAR > 3P0G-BI	LDM 004	1.79	39.5	6.2	Fig 7
pharmacology switch	M2R	4MQS-IXO > 3UON-QNB	4MQS-IXO	-	1.9	-46.6	S21 Fig
pharmacology switch	M2R	4MQS-IXO > 3UON-QNB	3UON-QNB	0.0	18.1	12.0	S21 Fig
pharmacology switch	M2R	4MQS-IXO > 3UON-QNB	LDM 000	2.75	5.5	-26.8	S21 Fig
pharmacology switch	M2R	3UON-QNB > 4MQS-IXO	3UON-QNB	-	-2.6	3.7	S20 Fig
pharmacology switch	M2R	3UON-QNB > 4MQS-IXO	4MQS-IXO	0.0	34.4	78.0	S20 Fig
pharmacology switch	M2R	3UON-QNB > 4MQS-IXO	LDM 000	8.63	6.6	12.5	S20 Fig
pharmacology switch	M2R	3UON-QNB > 4MQS-IXO	LDM 001	2.92	23.4	18.4	S20 Fig
pharmacology switch	M2R	3UON-QNB > 4MQS-IXO	LDM 002	2.92	20.1	14.1	S20 Fig
pharmacology switch	M2R	3UON-QNB > 4MQS-IXO	LDM 003	8.39	18.7	12.3	S20 Fig
pharmacology switch	M2R	3UON-QNB > 4MQS-IXO	LDM 018	3.05	-16.9	6.2	S20 Fig
