# Supplementary material for: Improving virtual screening of G protein-coupled receptors via ligand-directed modeling
Source: PLoS Comput Biol. 2017 Nov 13;13(11):e1005819. doi: 10.1371/journal.pcbi.1005819 (PMC5708846; doi:10.1371/journal.pcbi.1005819)
Supplement: S1 Text — Description of the required software, file preparation and parameters to run the LDM. (PDF) [file pcbi.1005819.s004.pdf]

**S1 Text. Executing the LDM scripts**. Description of the required software, file preparation and parameters to run the LDM.

## Program installation and set up for use in LDM

Install Molsoft ICM [1], FFTW 3.3.3 (http://www.fftw.org/), GROMACS 4.0.7 [2], CONCOORD 2.1 [3], tCONCOORD 1.0 [4], OPUS_PSP 1.0 [5] and multiscale modeling tool for structural biology (MMTSB) [6]. Set environment variables and source the edited run command files where required by the documentation of these software. In order to execute the LDM scripts, the binaries for CONCOORD must be renamed from dist and disco to dist-concoord and disco-concoord, respectively.

## File preparations for LDM

Edit the control-script found in the LDM scripts to contain the paths for ICM and OPUS_PSP software and insert the username that will run the LDM on the computer cluster employed.

The receptor file should be edited to remove residues that are not to be included in the simulation (such as extended loops) and residue numbering should be updated to start at 1 with each residue numbered without any gaps. Parameters from the receptor file that are required to run the LDM include (i) residue numbers that correspond to the start and end of each TM region, this also includes any loop portion that is to be included in the simulation, (ii) residue numbers that form the binding pocket, (iii) residue numbers of the cysteines that form the conserved disulfide bond between ECL2 and TM3, (iv) residue numbers that define the cytoplasmic end of the receptor, which can be identified based on their Ballesteros-Weinstein nomenclature as follows 1.48, 2.51, 3.38, 4.51, 5.50, 6.43, 7.45. The number of the last residue of the receptor should be added to this list of cytoplasmic residues. The receptor file name and residue numbers for the TM start and ends, docking region, disulfide bond and cytoplasmic residues should be entered in the control-script by modifying the variables receptor=, tm1s=, tm1e=, …, tm7s=, tm7e=, disul1=a_1/ and disul2=a_2/, cytoplasmicresidues= and dockingregion=a_1/, respectively.

Prepare the ligand by running energy minimisation and saving it in .mol2 format, which can be performed in ICM. Save the ligand with a three letter name (e.g. XXX) and edit that name in the .mol2 file by changing every instance of RES1 to the ligand name (e.g. XXX1). The ligand file name and ligand name in the .mol2 file should be edited in the control-script by modifying the variables ligand= and ligandname=, respectively.

Finally, the run name should be edited by modifying the runname= variable in the control-script, while leaving the "-xxxyyyxxx-" as a suffix (e.g. runname=my_new_run-xxxyyyxxx).

## LDM parameters

The user can modify parameters within the LDM workflow by editing the control-script file. The following parameters were used in this study: dirs=20 (number of independent directories), concoordruns=30 (number of CONCOORD receptor conformations sampled), concoordsides=5 (number of tCONCOORD side chain conformations sampled) and noofrounds=8 (number of iterative rounds). Finally, the number of independent replicas can be edited by modifying the “mkdir” file. 40 independent replicas were used in this study.

### LDM run and analysis

The LDM independent replicas do not need to be ran concurrently, providing more flexibility to users that do not have access to many compute cores at one time. On average one replica runs for 30±5 hours on 1120 Intel Sandybridge compute cores running at 2.7GHz. Once the LDM run has completed the desired number of replicas, which was set at 40 for this study, the ldm_results.py script is used to extract the ranked LDM complexes. The OPUS-ICM scoring was used in this study. The conformations of a top ranked set of LDM complexes can be analysed using the toolbx_pdb package (https://github.com/thomas-coudrat/toolbx_pdb) [7].

## References


1. Abagyan R, Totrov M, Kuznetsov D. ICM—A new method for protein modeling and design: Applications to docking and structure prediction from the distorted native conformation. J Comput Chem. 1994;15: 488–506.

2. Hess B, Kutzner C, van der Spoel D, Lindahl E. GROMACS 4: algorithms for highly efficient, load-balanced, and scalable molecular simulation. J Chem Theory Comput. 2008;4: 435–447.

3. de Groot BL, van Aalten DM, Scheek RM, Amadei A, Vriend G, Berendsen HJ. Prediction of protein conformational freedom from distance constraints. Proteins. 1997;29: 240–251.

4. Seeliger D, Haas J, de Groot BL. Geometry-based sampling of conformational transitions in proteins. Structure. 2007;15: 1482–1492.

5. Lu M, Dousis AD, Ma J. OPUS-PSP: an orientation-dependent statistical all-atom potential derived from side-chain packing. J Mol Biol. 2008;376: 288–301.

6. Feig M, Karanicolas J, Brooks CL. MMTSB Tool Set: enhanced sampling and multiscale modeling methods for applications in structural biology. J Mol Graph Model. 2004;22: 377–395.

7. Coudrat T, Christopoulos A, Sexton PM, Wootten D. Structural features embedded in G protein-coupled receptor co-crystal structures are key to their success in virtual screening. PLoS One. 2017;12: e0174719.
