# Supplementary figures and images for: Improving virtual screening of G protein-coupled receptors via ligand-directed modeling
Source: PLoS Comput Biol. 2017 Nov 13;13(11):e1005819. doi: 10.1371/journal.pcbi.1005819 (PMC5708846; doi:10.1371/journal.pcbi.1005819)

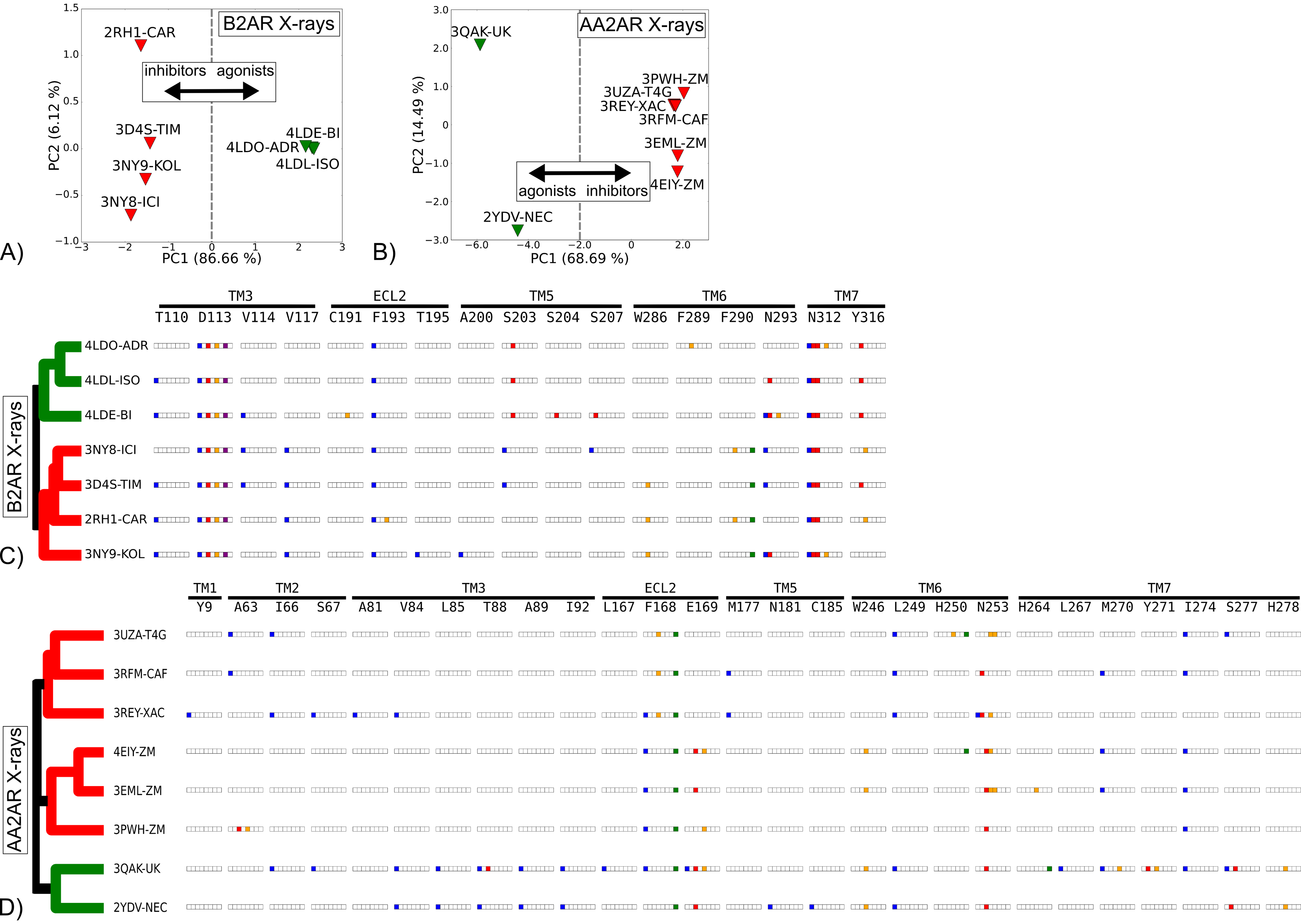

Supplement: S1 Fig — Principal component analysis on the binding pocket residue coordinates of A) B2AR and B) AA2AR X-ray structures used in this study. Principal components (e.g. PC1 and PC2) are ordered based on their percentage of cumulative variance explained from the original dataset (e.g. PC1 explains 50% of the data variance). An arbitrary line drawn along the PC1 axis, which explains most of the variance in the dataset, separates agonist-bound and inhibitor-bound X-ray structures based on their binding pocket conformations. Dendrogram of the IFP clustering and IFP diagrams on the ligand/receptor interaction patterns for C) B2AR and D) AA2AR X-ray structures used in this study. The IFP dendrogram branches and the PCA labels representing agonist-bound and inhibitor-bound X-ray structures are colored green and red, respectively. (TIF) [file pcbi.1005819.s005.tif]

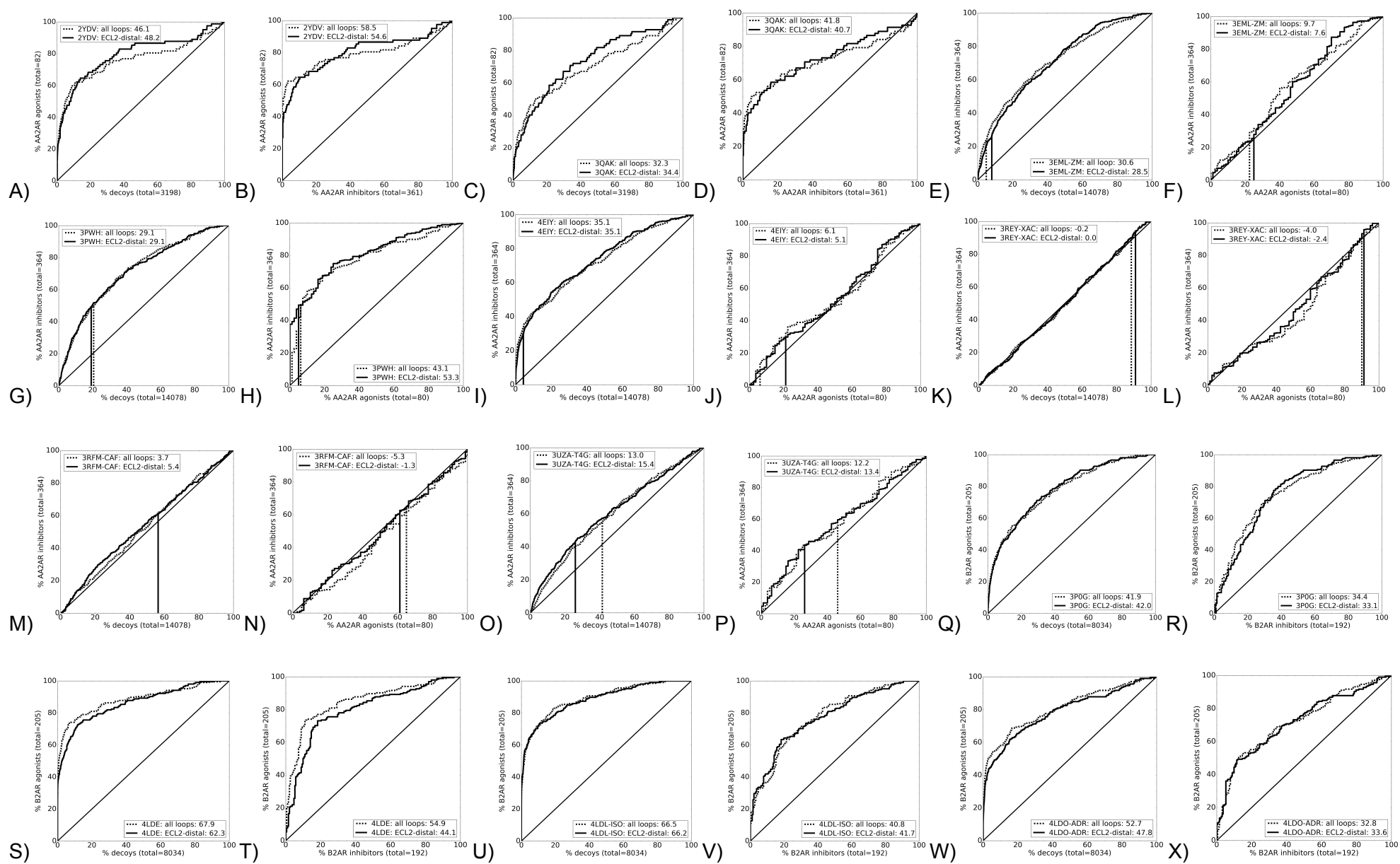

Supplement: S3 Fig — VS performance evaluated as recovery of known ligands against decoys and selectivity of agonists over inhibitors (or vice-versa). All loop structures are represented by black dotted lines and ECL2-distal only structures are represented by solid black lines. NSQ_AUC values for each curve is shown in the figure inset. AA2AR 2YDV-NEC recovery (A) and selectivity (B), AA2AR 3QAK-UK recovery (C) and selectivity (D), AA2AR 3EML-ZM recovery (E) and selectivity (F), AA2AR 3PWH-ZM recovery (G) and selectivity (H), AA2AR 4EIY-ZM recovery (I) and selectivity (J), AA2AR 3REY-XAC recovery (K) and selectivity (L), AA2AR 3RFM-CAF recovery (M) and selectivity (N), AA2AR 3UZA-T4G recovery (O) and selectivity (P), B2AR 3P0G-BI recovery (Q) and selectivity (R), B2AR 4LDE-BI recovery (S) and selectivity (T), B2AR 4LDL-ISO recovery (U) and selectivity (V), B2AR 4LDO-ADR recovery (W) and selectivity (X). (PDF) [file pcbi.1005819.s007.pdf]

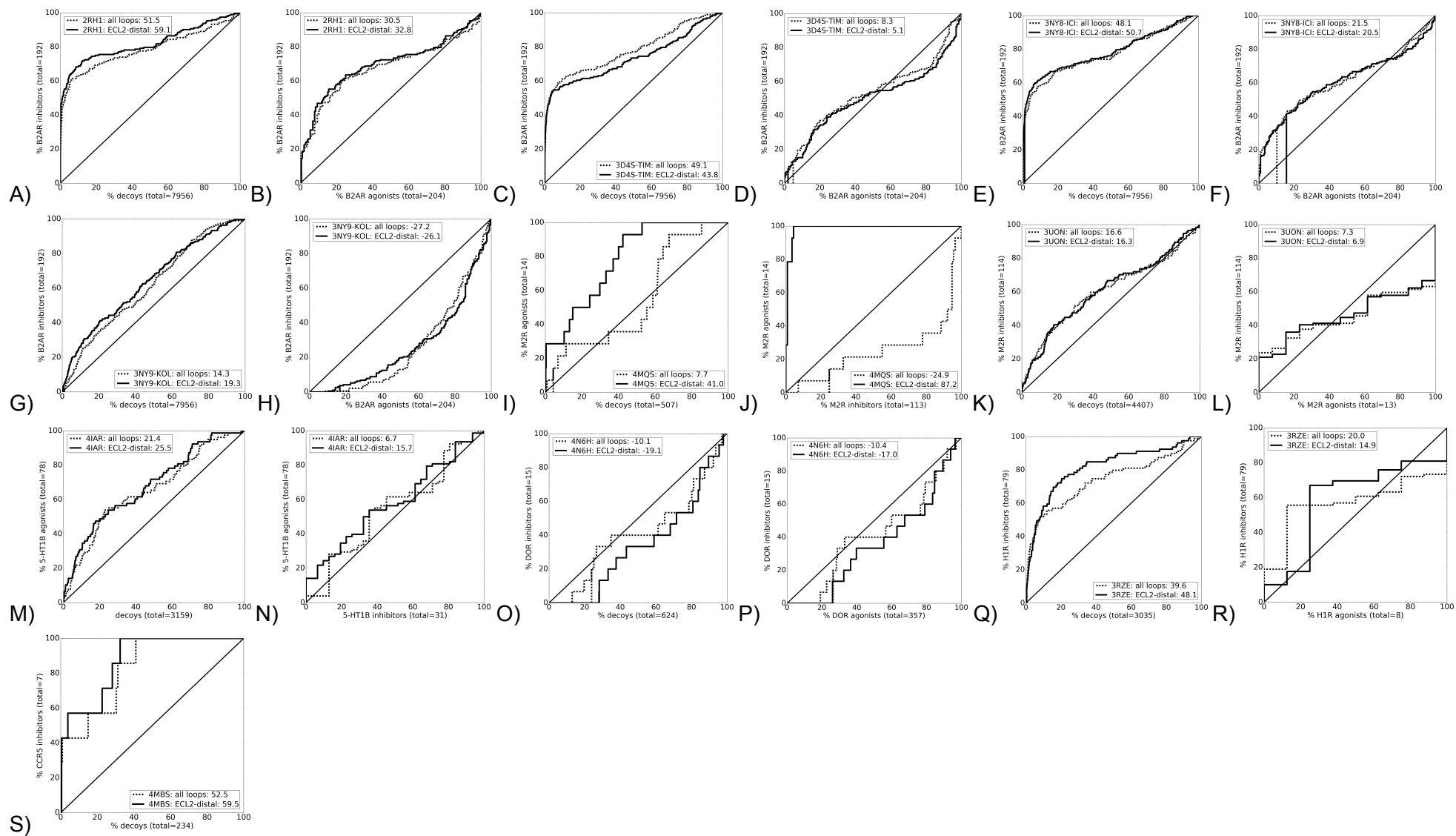

Supplement: S4 Fig — VS performance evaluated as recovery of known ligands against decoys and selectivity of agonists over inhibitors (or vice-versa). All loop structures are represented by black dotted lines and ECL2-distal only structures are represented by solid black lines. NSQ_AUC values for each curve is shown in the figure inset. B2AR 2RH1-CAR recovery (A) and selectivity (B), B2AR 3D4S-TIM recovery (C) and selectivity (D), B2AR 3NY8-ICI recovery (E) and selectivity (F), B2AR 3NY9-KOL recovery (G) and selectivity (H), M2R 4MQS-IXO recovery (I) and selectivity (J), M2R 3UON-QNB recovery (K) and selectivity (L), 5-HT1B 4IAR-ERG recovery (M) and selectivity (N), DOR 4N6H-NAL (3D library) recovery (O) and selectivity (P), H1R 3RZE-DOX recovery (Q) and selectivity (R), CCR5 4MBS-MRV recovery (S). (PDF) [file pcbi.1005819.s008.pdf]

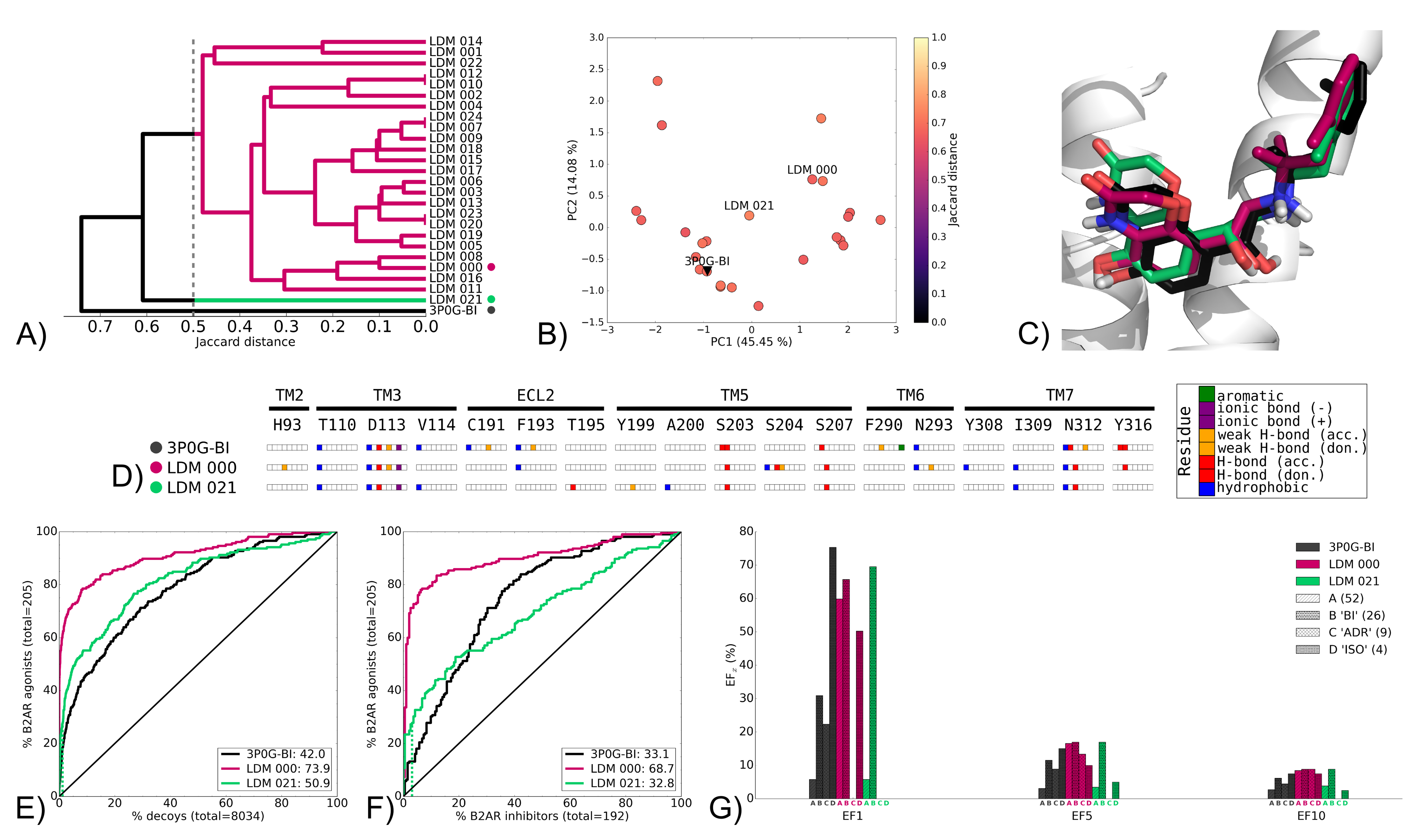

Supplement: S5 Fig — A) Dendrogram of the top 25 LDM models and X-ray structure(s), a cutoff line identifies different LDM clusters and their representative LDM models are designated by a colored dot. Representative LDM models are the highest scoring within the cluster based on the OPUS-ICM metric. B) Comparison of binding pocket conformation between the top 25 LDM models and X-ray structure(s). LDM models are colored based on their IFP Jaccard distance with the destination X-ray structure. C) Binding poses of the representative LDM model(s) and the destination X-ray structure. D) IFP of the representative LDM models and the X-ray structure. Interaction type is described for each residue of the binding pocket: hydrophobic interaction, hydrogen bond (H-bond) donor and acceptor, weak hydrogen bond (weak H-bond) donor and acceptor, ionic bond positive (+) and negative (-) and aromatic interaction. E) the recovery of known ligands vs. decoys and F) the selectivity of inhibitors over agonists (or vice-versa). The relative rank of the LDM refinement ligand is identified with a vertical dashed line. This vertical line may be masked by other curves if the ligand is very highly ranked. The ROC curve figure inset shows NSQ_AUC values for each binding pocket. Finally, a G) bar chart is used to visualise the EF for representative known ligand chemotypes at EF1, EF5 and EF10. Chemotypes A, B ‘BI-like’, C ‘ADR-like’ and D ‘ISO-like’ represent only a subset of B2AR agonist ligands (S2 Fig). The EF bar chart inset shows the number of ligands for each chemotype cluster between parenthesis. X-ray structure chemotype EF shown in black bars, with the LDM models coloured based on their relative clusters identified in A. (TIF) [file pcbi.1005819.s009.tif]

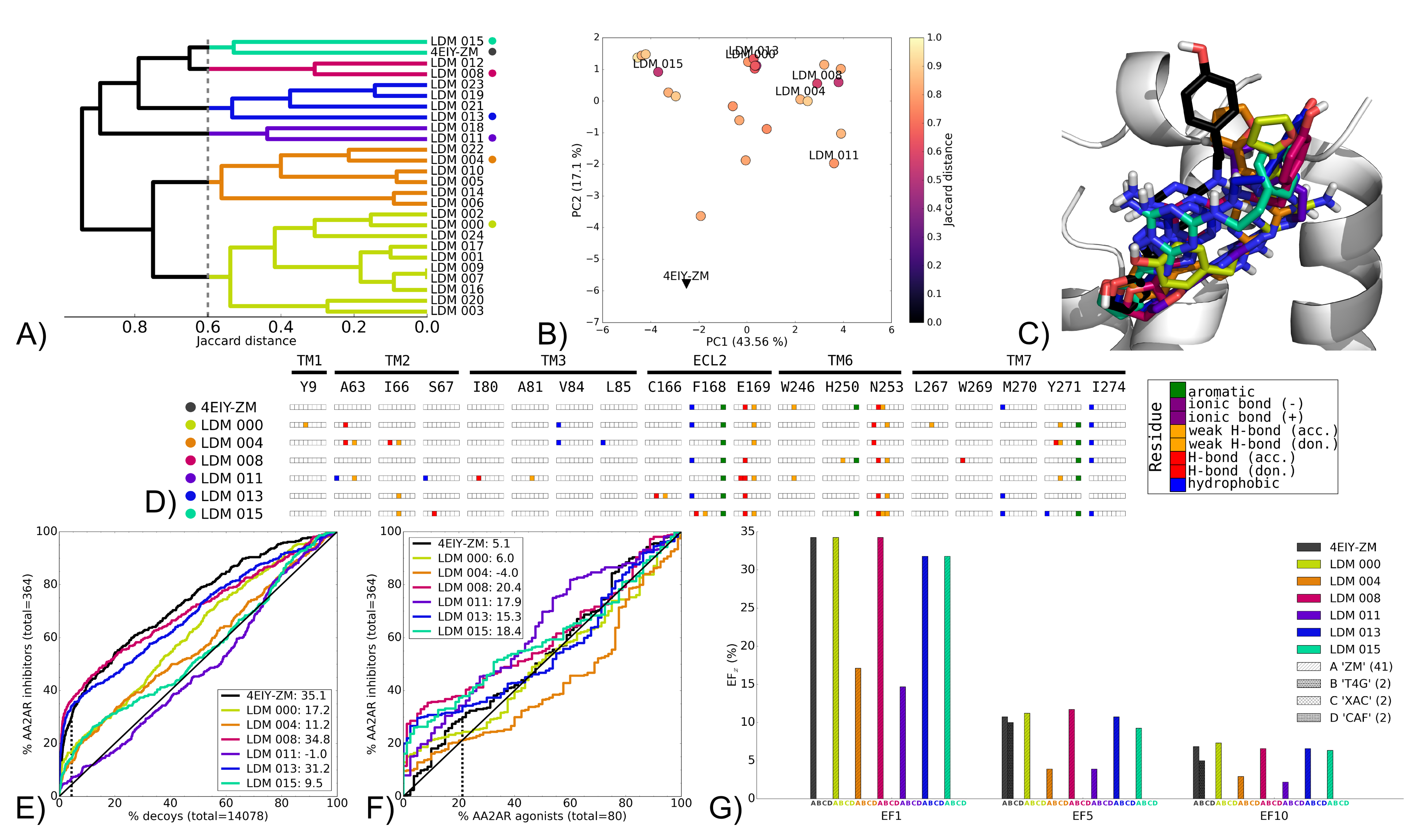

Supplement: S6 Fig — A) Dendrogram of the top 25 LDM models and X-ray structure(s), a cutoff line identifies different LDM clusters and their representative LDM models are designated by a colored dot. Representative LDM models are the highest scoring within the cluster based on the OPUS-ICM metric. B) Comparison of binding pocket conformation between the top 25 LDM models and X-ray structure(s). LDM models are colored based on their IFP Jaccard distance with the destination X-ray structure. C) Binding poses of the representative LDM model(s) and the destination X-ray structure. D) IFP of the representative LDM models and the X-ray structure. Interaction type is described for each residue of the binding pocket: hydrophobic interaction, hydrogen bond (H-bond) donor and acceptor, weak hydrogen bond (weak H-bond) donor and acceptor, ionic bond positive (+) and negative (-) and aromatic interaction. VS performance is described with ROC curves to visualise E) the recovery of known ligands vs. decoys and F) the selectivity of inhibitors over agonists (or vice-versa). The relative rank of the LDM refinement ligand is identified with a vertical dashed line. This vertical line may be masked by other curves if the ligand is very highly ranked. The ROC curve figure inset shows NSQ_AUC values for each binding pocket. Finally, a G) bar chart is used to visualise the EF for representative known ligand chemotypes at EF1, EF5 and EF10. Chemotypes A ‘ZM-like’, B ‘T4G-like’, C ‘XAC-like’ and D ‘CAF-like’ represent only a subset of AA2AR inhibitors ligands (S2 Fig). The EF bar chart inset shows the number of ligands for each chemotype cluster between parenthesis. X-ray structure chemotype EF shown in black bars, with the LDM models coloured based on their relative clusters identified in A. (TIF) [file pcbi.1005819.s010.tif]

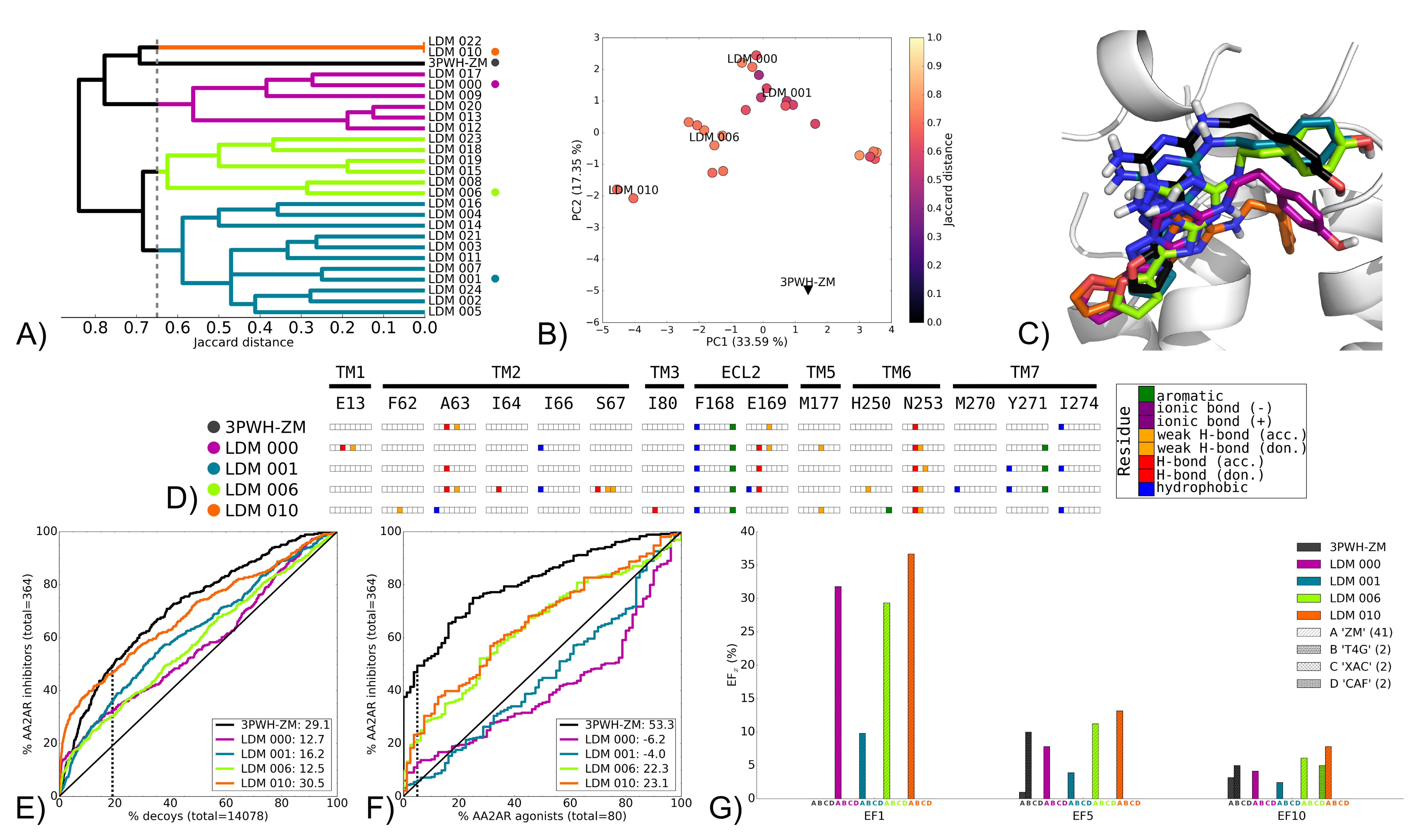

Supplement: S7 Fig — A) Dendrogram of the top 25 LDM models and X-ray structure(s), a cutoff line identifies different LDM clusters and their representative LDM models are designated by a colored dot. Representative LDM models are the highest scoring within the cluster based on the OPUS-ICM metric. B) Comparison of binding pocket conformation between the top 25 LDM models and X-ray structure(s). LDM models are colored based on their IFP Jaccard distance with the destination X-ray structure. C) Binding poses of the representative LDM model(s) and the destination X-ray structure. D) IFP of the representative LDM models and the X-ray structure. Interaction type is described for each residue of the binding pocket: hydrophobic interaction, hydrogen bond (H-bond) donor and acceptor, weak hydrogen bond (weak H-bond) donor and acceptor, ionic bond positive (+) and negative (-) and aromatic interaction. VS performance is described with ROC curves to visualise E) the recovery of known ligands vs. decoys and F) the selectivity of inhibitors over agonists (or vice-versa). The relative rank of the LDM refinement ligand is identified with a vertical dashed line. This vertical line may be masked by other curves if the ligand is very highly ranked. The ROC curve figure inset shows NSQ_AUC values for each binding pocket. Finally, a G) bar chart is used to visualise the EF for representative known ligand chemotypes at EF1, EF5 and EF10. Chemotypes A ‘ZM-like’, B ‘T4G-like’, C ‘XAC-like’ and D ‘CAF-like’ represent only a subset of AA2AR inhibitors ligands (S2 Fig). The EF bar chart inset shows the number of ligands for each chemotype cluster between parenthesis. X-ray structure chemotype EF shown in black bars, with the LDM models coloured based on their relative clusters identified in A. (TIF) [file pcbi.1005819.s011.tif]

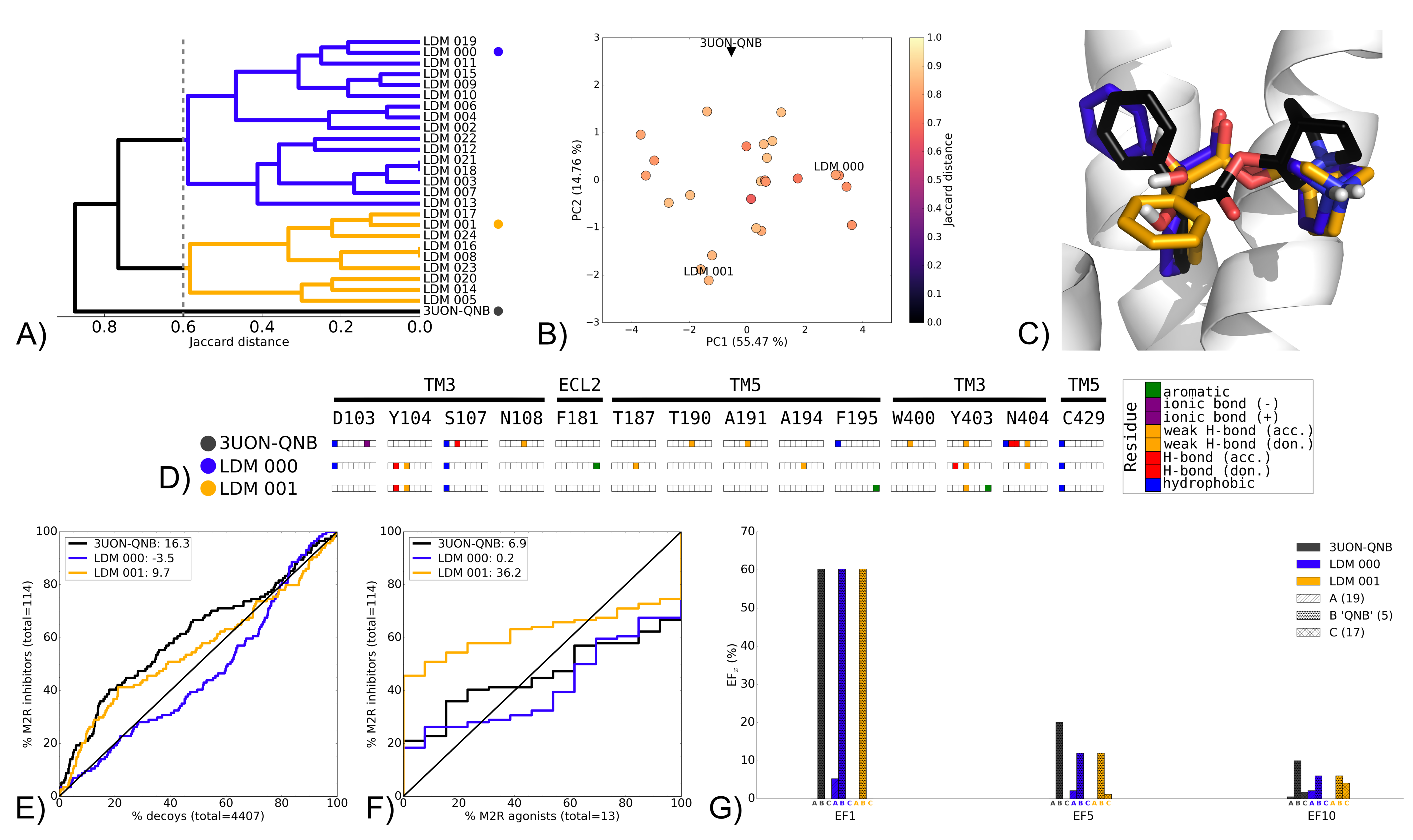

Supplement: S8 Fig — A) Dendrogram of the top 25 LDM models and X-ray structure(s), a cutoff line identifies different LDM clusters and their representative LDM models are designated by a colored dot. Representative LDM models are the highest scoring within the cluster based on the OPUS-ICM metric. B) Comparison of binding pocket conformation between the top 25 LDM models and X-ray structure(s). LDM models are colored based on their IFP Jaccard distance with the destination X-ray structure. C) Binding poses of the representative LDM model(s) and the destination X-ray structure. D) IFP of the representative LDM models and the X-ray structure. Interaction type is described for each residue of the binding pocket: hydrophobic interaction, hydrogen bond (H-bond) donor and acceptor, weak hydrogen bond (weak H-bond) donor and acceptor, ionic bond positive (+) and negative (-) and aromatic interaction. VS performance is described with ROC curves to visualise E) the recovery of known ligands vs. decoys and F) the selectivity of inhibitors over agonists (or vice-versa). The relative rank of the LDM refinement ligand is identified with a vertical dashed line. This vertical line may be masked by other curves if the ligand is very highly ranked. The ROC curve figure inset shows NSQ_AUC values for each binding pocket. Finally, a G) bar chart is used to visualise the EF for representative known ligand chemotypes at EF1, EF5 and EF10. Chemotypes A, B ‘QNB-like’ and C represent only a subset of M2R inhibitor ligands (S2 Fig). The EF bar chart inset shows the number of ligands for each chemotype cluster between parenthesis. X-ray structure chemotype EF shown in black bars, with the LDM models coloured based on their relative clusters identified in A. (TIF) [file pcbi.1005819.s012.tif]

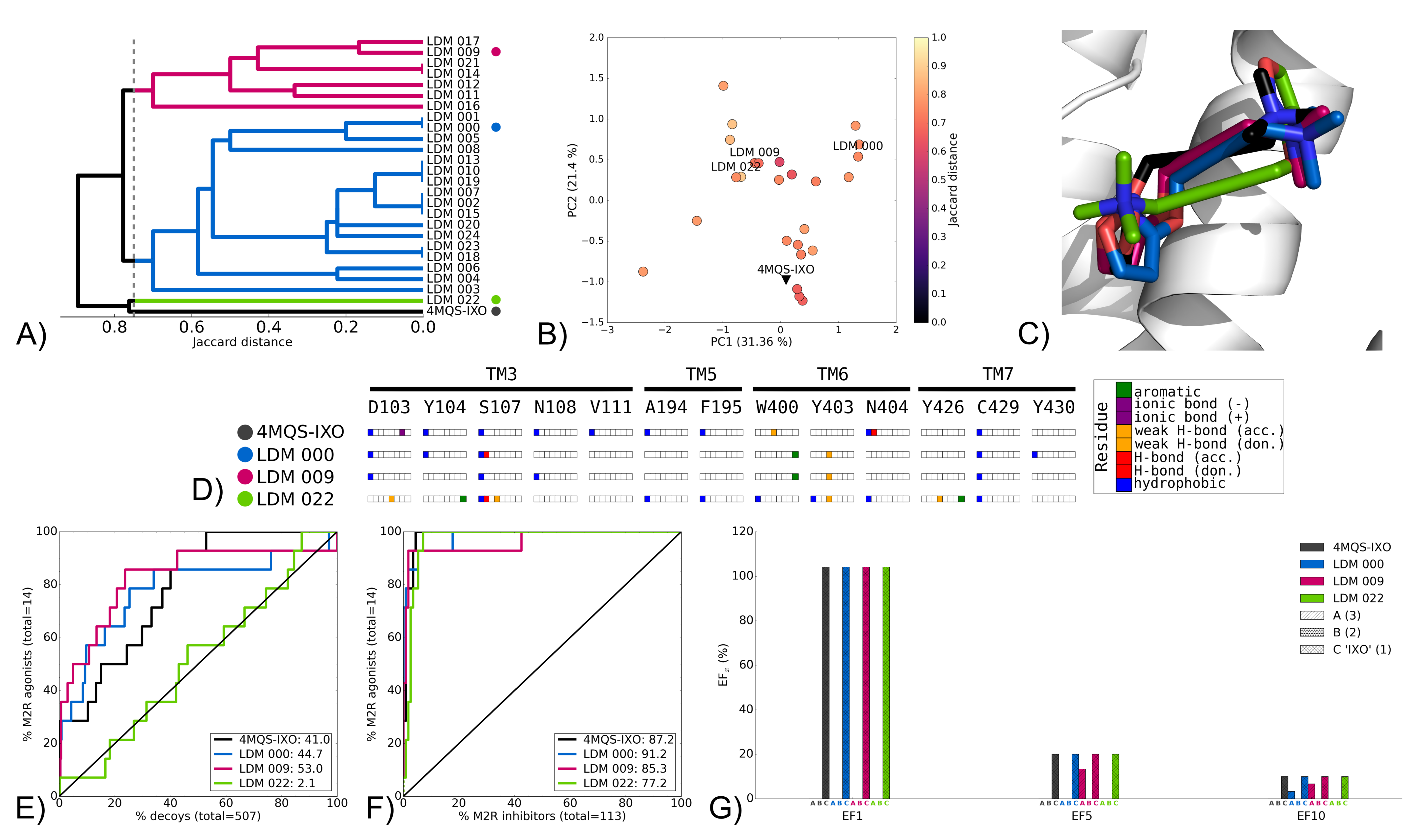

Supplement: S9 Fig — A) Dendrogram of the top 25 LDM models and X-ray structure(s), a cutoff line identifies different LDM clusters and their representative LDM models are designated by a colored dot. Representative LDM models are the highest scoring within the cluster based on the OPUS-ICM metric. B) Comparison of binding pocket conformation between the top 25 LDM models and X-ray structure(s). LDM models are colored based on their IFP Jaccard distance with the destination X-ray structure. C) Binding poses of the representative LDM model(s) and the destination X-ray structure. D) IFP of the representative LDM models and the X-ray structure. Interaction type is described for each residue of the binding pocket: hydrophobic interaction, hydrogen bond (H-bond) donor and acceptor, weak hydrogen bond (weak H-bond) donor and acceptor, ionic bond positive (+) and negative (-) and aromatic interaction. VS performance is described with ROC curves to visualise E) the recovery of known ligands vs. decoys and F) the selectivity of inhibitors over agonists (or vice-versa). The relative rank of the LDM refinement ligand is identified with a vertical dashed line. This vertical line may be masked by other curves if the ligand is very highly ranked. The ROC curve figure inset shows NSQ_AUC values for each binding pocket. Finally, a G) bar chart is used to visualise the EF for representative known ligand chemotypes at EF1, EF5 and EF10. Chemotypes A, B and C ‘IXO-like’ represent only a subset of M2R agonist ligands (S2 Fig). The EF bar chart inset shows the number of ligands for each chemotype cluster between parenthesis. X-ray structure chemotype EF shown in black bars, with the LDM models coloured based on their relative clusters identified in A. (TIF) [file pcbi.1005819.s013.tif]

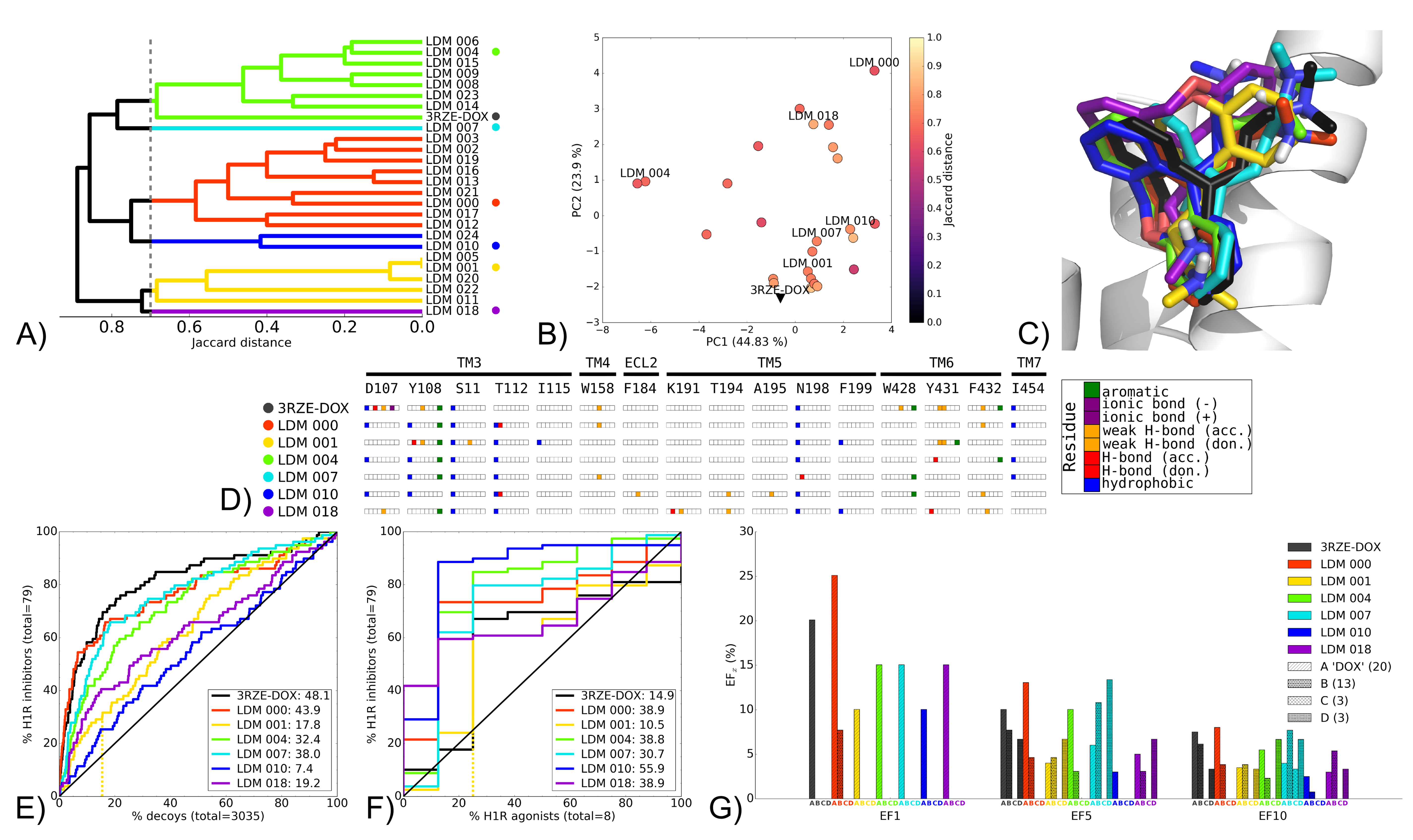

Supplement: S10 Fig — A) Dendrogram of the top 25 LDM models and X-ray structure(s), a cutoff line identifies different LDM clusters and their representative LDM models are designated by a colored dot. Representative LDM models are the highest scoring within the cluster based on the OPUS-ICM metric. B) Comparison of binding pocket conformation between the top 25 LDM models and X-ray structure(s). LDM models are colored based on their IFP Jaccard distance with the destination X-ray structure. C) Binding poses of the representative LDM model(s) and the destination X-ray structure. D) IFP of the representative LDM models and the X-ray structure. Interaction type is described for each residue of the binding pocket: hydrophobic interaction, hydrogen bond (H-bond) donor and acceptor, weak hydrogen bond (weak H-bond) donor and acceptor, ionic bond positive (+) and negative (-) and aromatic interaction. VS performance is described with ROC curves to visualise E) the recovery of known ligands vs. decoys and F) the selectivity of inhibitors over agonists (or vice-versa). The relative rank of the LDM refinement ligand is identified with a vertical dashed line. This vertical line may be masked by other curves if the ligand is very highly ranked. The ROC curve figure inset shows NSQ_AUC values for each binding pocket. Finally, a G) bar chart is used to visualise the EF for representative known ligand chemotypes at EF1, EF5 and EF10. Chemotypes A ‘DOX-like’, B, C and D represent only a subset of H1R inhibitor ligands (S2 Fig). The EF bar chart inset shows the number of ligands for each chemotype cluster between parenthesis. X-ray structure chemotype EF shown in black bars, with the LDM models coloured based on their relative clusters identified in A. (TIF) [file pcbi.1005819.s014.tif]

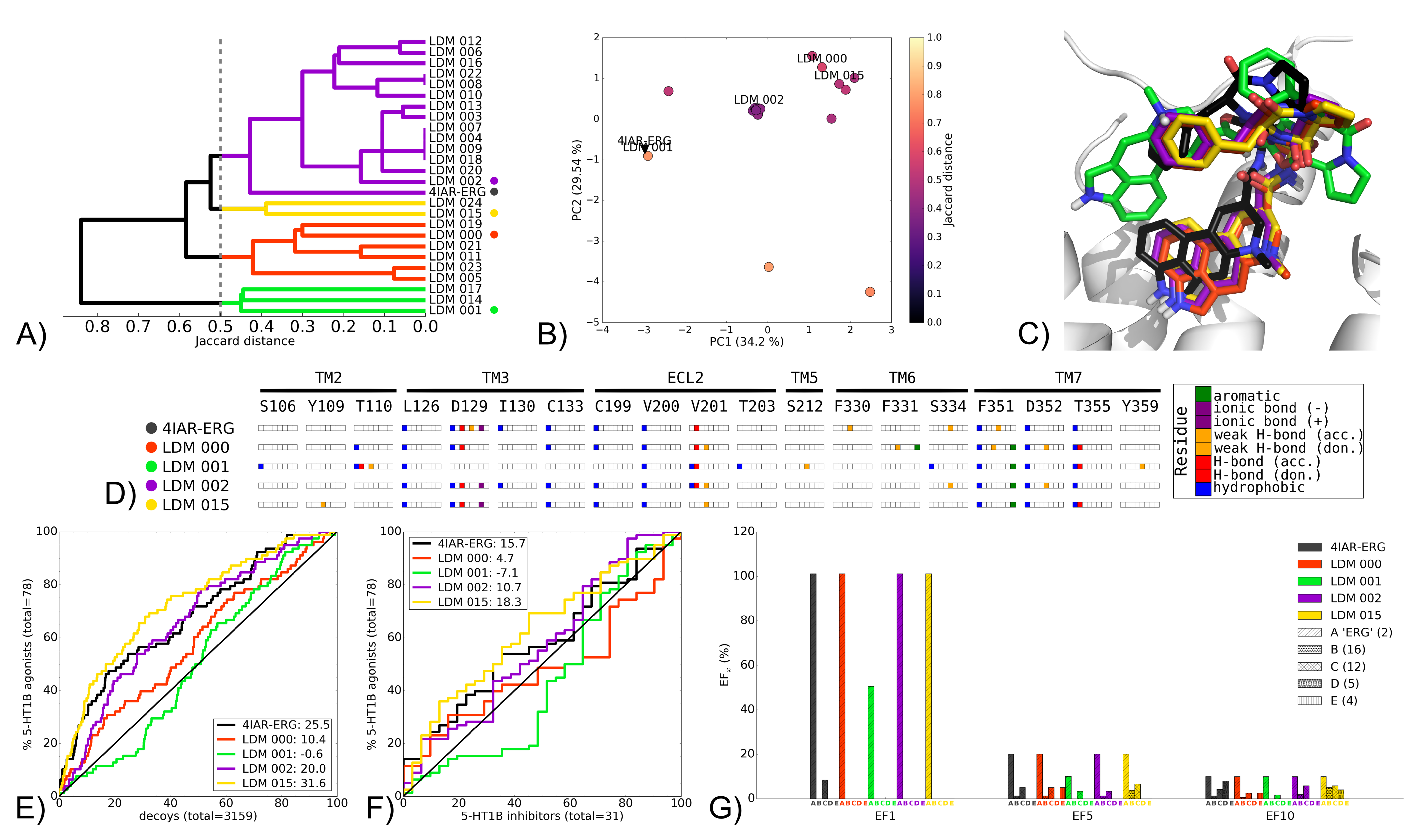

Supplement: S11 Fig — A) Dendrogram of the top 25 LDM models and X-ray structure(s), a cutoff line identifies different LDM clusters and their representative LDM models are designated by a colored dot. Representative LDM models are the highest scoring within the cluster based on the OPUS-ICM metric. B) Comparison of binding pocket conformation between the top 25 LDM models and X-ray structure(s). LDM models are colored based on their IFP Jaccard distance with the destination X-ray structure. C) Binding poses of the representative LDM model(s) and the destination X-ray structure. D) IFP of the representative LDM models and the X-ray structure. Interaction type is described for each residue of the binding pocket: hydrophobic interaction, hydrogen bond (H-bond) donor and acceptor, weak hydrogen bond (weak H-bond) donor and acceptor, ionic bond positive (+) and negative (-) and aromatic interaction. VS performance is described with ROC curves to visualise E) the recovery of known ligands vs. decoys and F) the selectivity of inhibitors over agonists (or vice-versa). The relative rank of the LDM refinement ligand is identified with a vertical dashed line. This vertical line may be masked by other curves if the ligand is very highly ranked. The ROC curve figure inset shows NSQ_AUC values for each binding pocket. Finally, a G) bar chart is used to visualise the EF for representative known ligand chemotypes at EF1, EF5 and EF10. Chemotypes A ‘ERG-like’, B, C, D and E represent only a subset of 5-HT1B agonist ligands (S2 Fig). The EF bar chart inset shows the number of ligands for each chemotype cluster between parenthesis. X-ray structure chemotype EF shown in black bars, with the LDM models coloured based on their relative clusters identified in A. (TIF) [file pcbi.1005819.s015.tif]

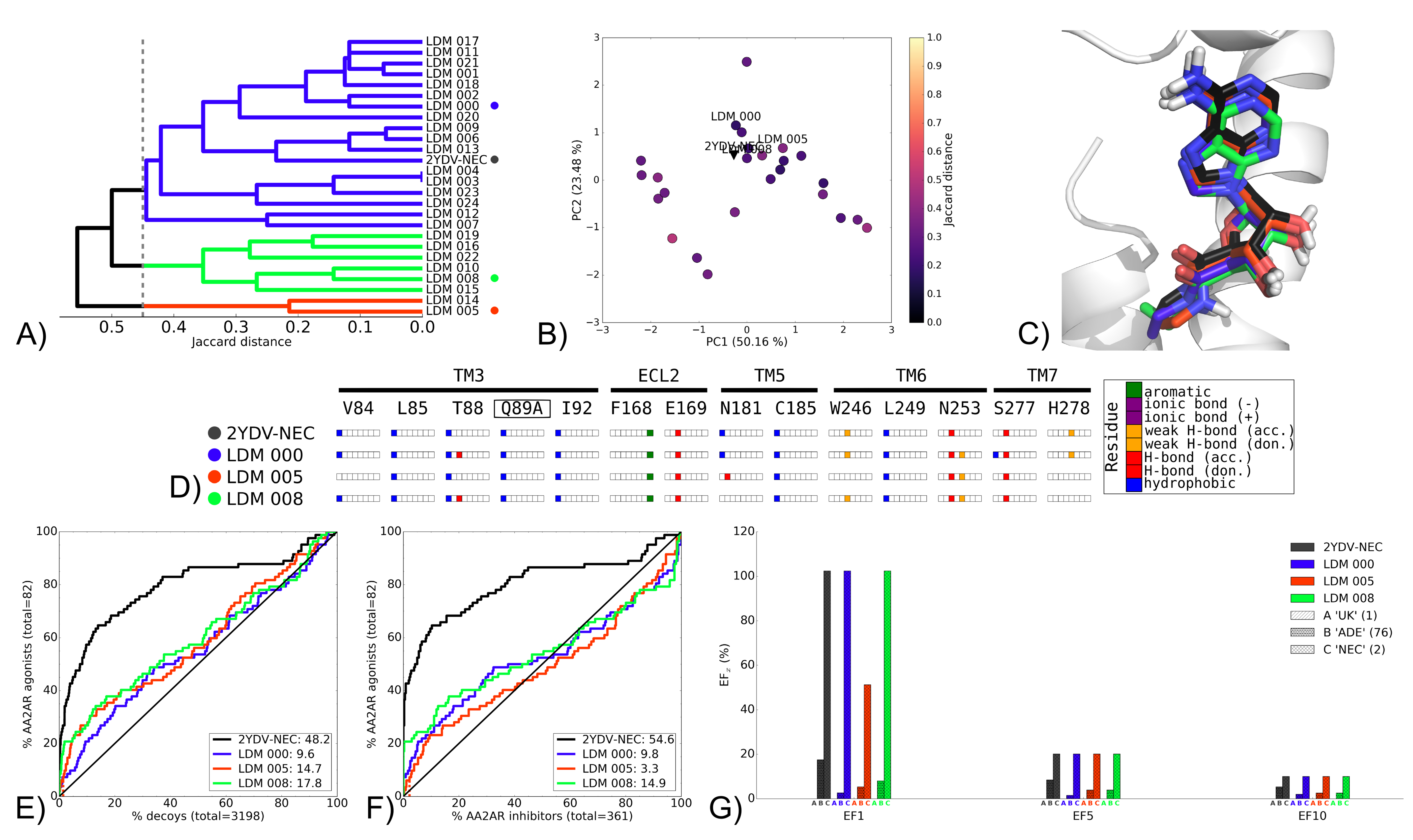

Supplement: S12 Fig — A) Dendrogram of the top 25 LDM models and X-ray structure(s), a cutoff line identifies different LDM clusters and their representative LDM models are designated by a colored dot. Representative LDM models are the highest scoring within the cluster based on the OPUS-ICM metric. B) Comparison of binding pocket conformation between the top 25 LDM models and X-ray structure(s). LDM models are colored based on their IFP Jaccard distance with the destination X-ray structure. C) Binding poses of the representative LDM model(s) and the destination X-ray structure. D) IFP of the representative LDM models and the X-ray structure. Interaction type is described for each residue of the binding pocket: hydrophobic interaction, hydrogen bond (H-bond) donor and acceptor, weak hydrogen bond (weak H-bond) donor and acceptor, ionic bond positive (+) and negative (-) and aromatic interaction. VS performance is described with ROC curves to visualise E) the recovery of known ligands vs. decoys and F) the selectivity of inhibitors over agonists (or vice-versa). The relative rank of the LDM refinement ligand is identified with a vertical dashed line. This vertical line may be masked by other curves if the ligand is very highly ranked. The ROC curve figure inset shows NSQ_AUC values for each binding pocket. Finally, a G) bar chart is used to visualise the EF for representative known ligand chemotypes at EF1, EF5 and EF10. Chemotypes A ‘UK-like’, B ‘ADE-like’ and C ‘NEC-like’ represent only a subset of AA2AR agonist ligands (S2 Fig). The EF bar chart inset shows the number of ligands for each chemotype cluster between parenthesis. X-ray structure chemotype EF shown in black bars, with the LDM models coloured based on their relative clusters identified in A. (TIF) [file pcbi.1005819.s016.tif]

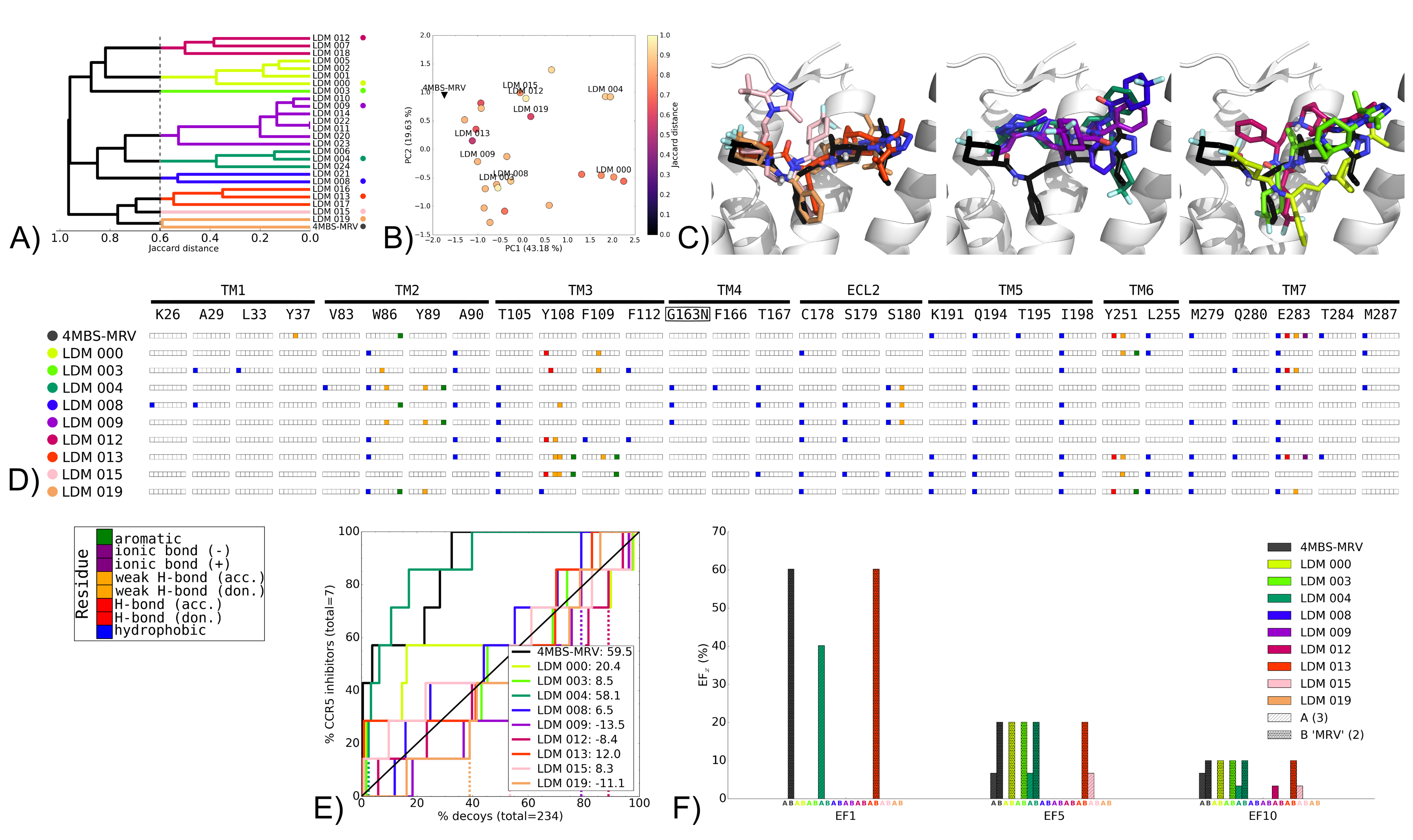

Supplement: S13 Fig — A) Dendrogram of the top 25 LDM models and X-ray structure(s), a cutoff line identifies different LDM clusters and their representative LDM models are designated by a colored dot. Representative LDM models are the highest scoring within the cluster based on the OPUS-ICM metric. B) Comparison of binding pocket conformation between the top 25 LDM models and X-ray structure(s). LDM models are colored based on their IFP Jaccard distance with the destination X-ray structure. C) Binding poses of the representative LDM model(s) and the destination X-ray structure. D) IFP of the representative LDM models and the X-ray structure. Interaction type is described for each residue of the binding pocket: hydrophobic interaction, hydrogen bond (H-bond) donor and acceptor, weak hydrogen bond (weak H-bond) donor and acceptor, ionic bond positive (+) and negative (-) and aromatic interaction. VS performance is described with ROC curves to visualise E) the recovery of known ligands vs. decoys and F) the selectivity of inhibitors over agonists (or vice-versa). The relative rank of the LDM refinement ligand is identified with a vertical dashed line. This vertical line may be masked by other curves if the ligand is very highly ranked. The ROC curve figure inset shows NSQ_AUC values for each binding pocket. Finally, a G) bar chart is used to visualise the EF for representative known ligand chemotypes at EF1, EF5 and EF10. Chemotypes A and B ‘MRV-like’ represent only a subset of CCR5 inhibitor ligands (S2 Fig). The EF bar chart inset shows the number of ligands for each chemotype cluster between parenthesis. X-ray structure chemotype EF shown in black bars, with the LDM models coloured based on their relative clusters identified in A. (TIF) [file pcbi.1005819.s017.tif]

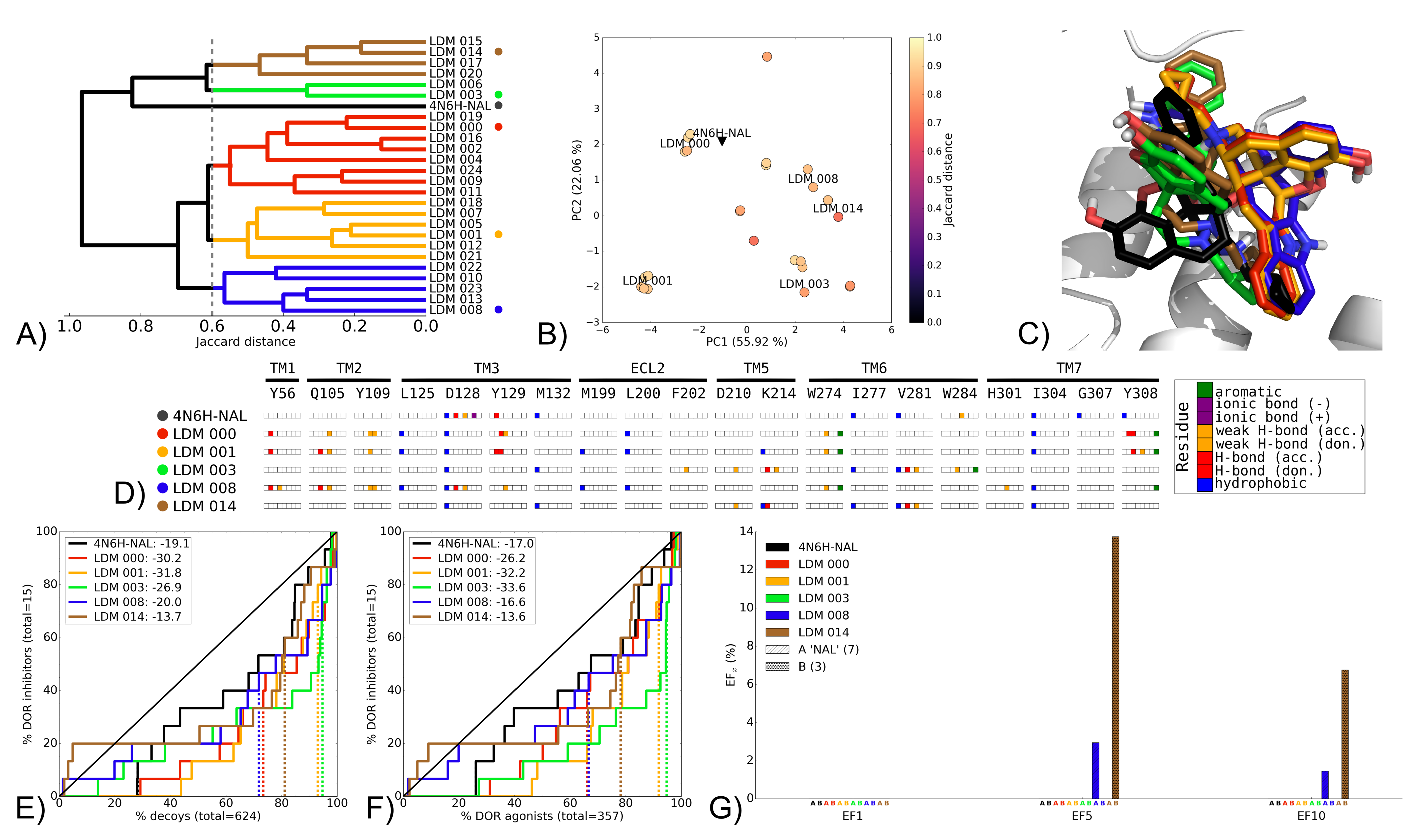

Supplement: S14 Fig — A) Dendrogram of the top 25 LDM models and X-ray structure(s), a cutoff line identifies different LDM clusters and their representative LDM models are designated by a colored dot. Representative LDM models are the highest scoring within the cluster based on the OPUS-ICM metric. B) Comparison of binding pocket conformation between the top 25 LDM models and X-ray structure(s). LDM models are colored based on their IFP Jaccard distance with the destination X-ray structure. C) Binding poses of the representative LDM model(s) and the destination X-ray structure. D) IFP of the representative LDM models and the X-ray structure. Interaction type is described for each residue of the binding pocket: hydrophobic interaction, hydrogen bond (H-bond) donor and acceptor, weak hydrogen bond (weak H-bond) donor and acceptor, ionic bond positive (+) and negative (-) and aromatic interaction. VS performance is described with ROC curves to visualise E) the recovery of known ligands vs. decoys and F) the selectivity of inhibitors over agonists (or vice-versa). The relative rank of the LDM refinement ligand is identified with a vertical dashed line. This vertical line may be masked by other curves if the ligand is very highly ranked. The ROC curve figure inset shows NSQ_AUC values for each binding pocket. Finally, a G) bar chart is used to visualise the EF for representative known ligand chemotypes at EF1, EF5 and EF10. Chemotypes A ‘NAL-like’ and B represent only a subset of DOR inhibitor ligands (S2 Fig). The EF bar chart inset shows the number of ligands for each chemotype cluster between parenthesis. X-ray structure chemotype EF shown in black bars, with the LDM models coloured based on their relative clusters identified in A. (TIF) [file pcbi.1005819.s018.tif]

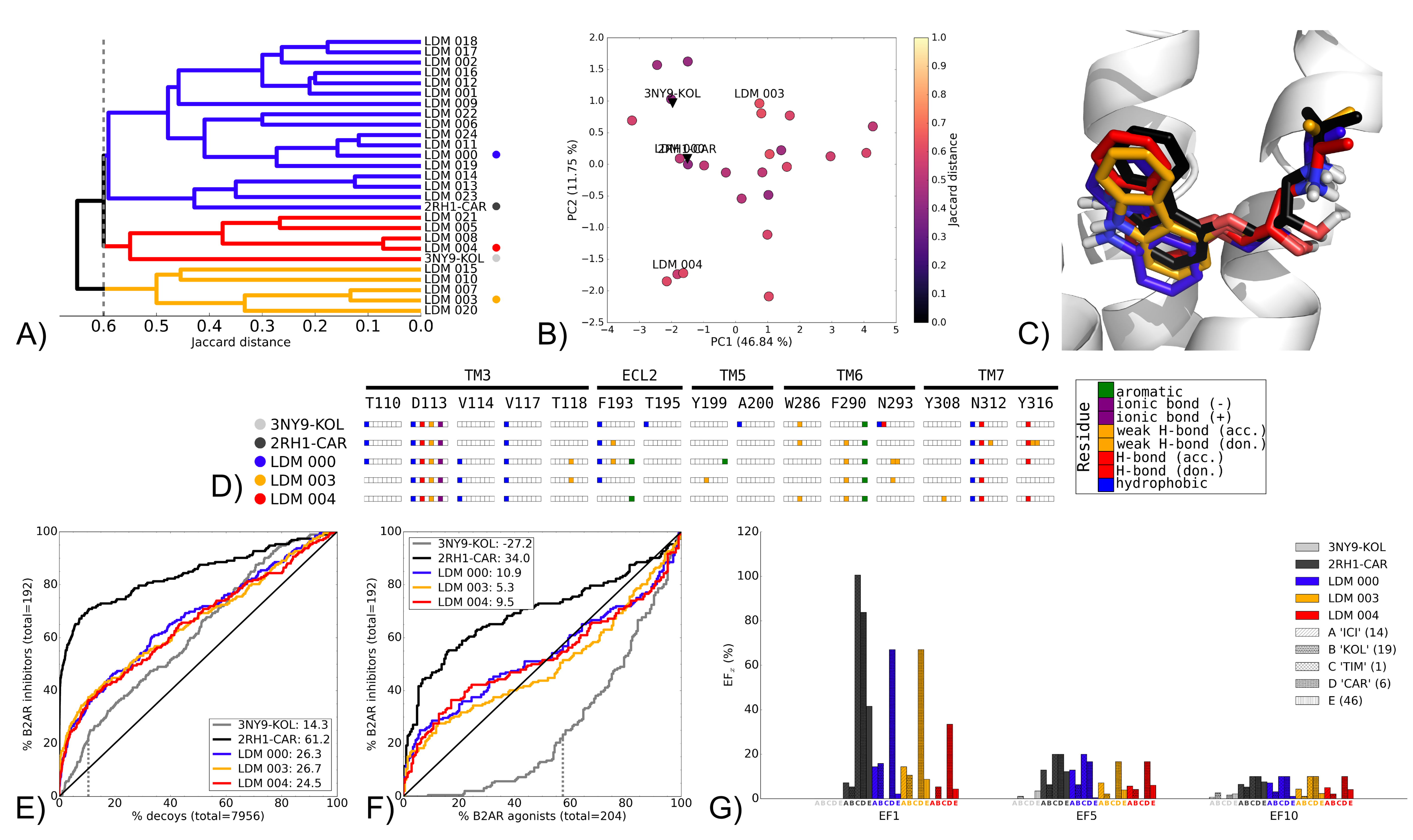

Supplement: S15 Fig — A) Dendrogram of the top 25 LDM models and X-ray structure(s), a cutoff line identifies different LDM clusters and their representative LDM models are designated by a colored dot. Representative LDM models are the highest scoring within the cluster based on the OPUS-ICM metric. B) Comparison of binding pocket conformation between the top 25 LDM models and X-ray structure(s). LDM models are colored based on their IFP Jaccard distance with the destination X-ray structure. C) Binding poses of the representative LDM model(s) and the destination X-ray structure. D) IFP of the representative LDM models and the X-ray structures. Interaction type is described for each residue of the binding pocket: hydrophobic interaction, hydrogen bond (H-bond) donor and acceptor, weak hydrogen bond (weak H-bond) donor and acceptor, ionic bond positive (+) and negative (-) and aromatic interaction. VS performance is described with ROC curves to visualise E) the recovery of known ligands vs. decoys and F) the selectivity of inhibitors over agonists (or vice-versa). The relative rank of the LDM refinement ligand is identified with a vertical dashed line. This vertical line may be masked by other curves if the ligand is very highly ranked. The ROC curve figure inset shows NSQ_AUC values for each binding pocket. Finally, a G) bar chart is used to visualise the EF for representative known ligand chemotypes at EF1, EF5 and EF10. Chemotypes A ‘ICI-like’, B ‘KOL-like’, C ‘TIM-like’, D ‘CAR-like’ and E represent only a subset of B2AR inhibitor ligands (S2 Fig). The EF bar chart inset shows the number of ligands for each chemotype cluster between parenthesis. Origin and destination X-ray structure chemotype EF shown in grey and black bars, respectively, with the LDM models coloured based on their relative clusters identified in A. (TIF) [file pcbi.1005819.s019.tif]

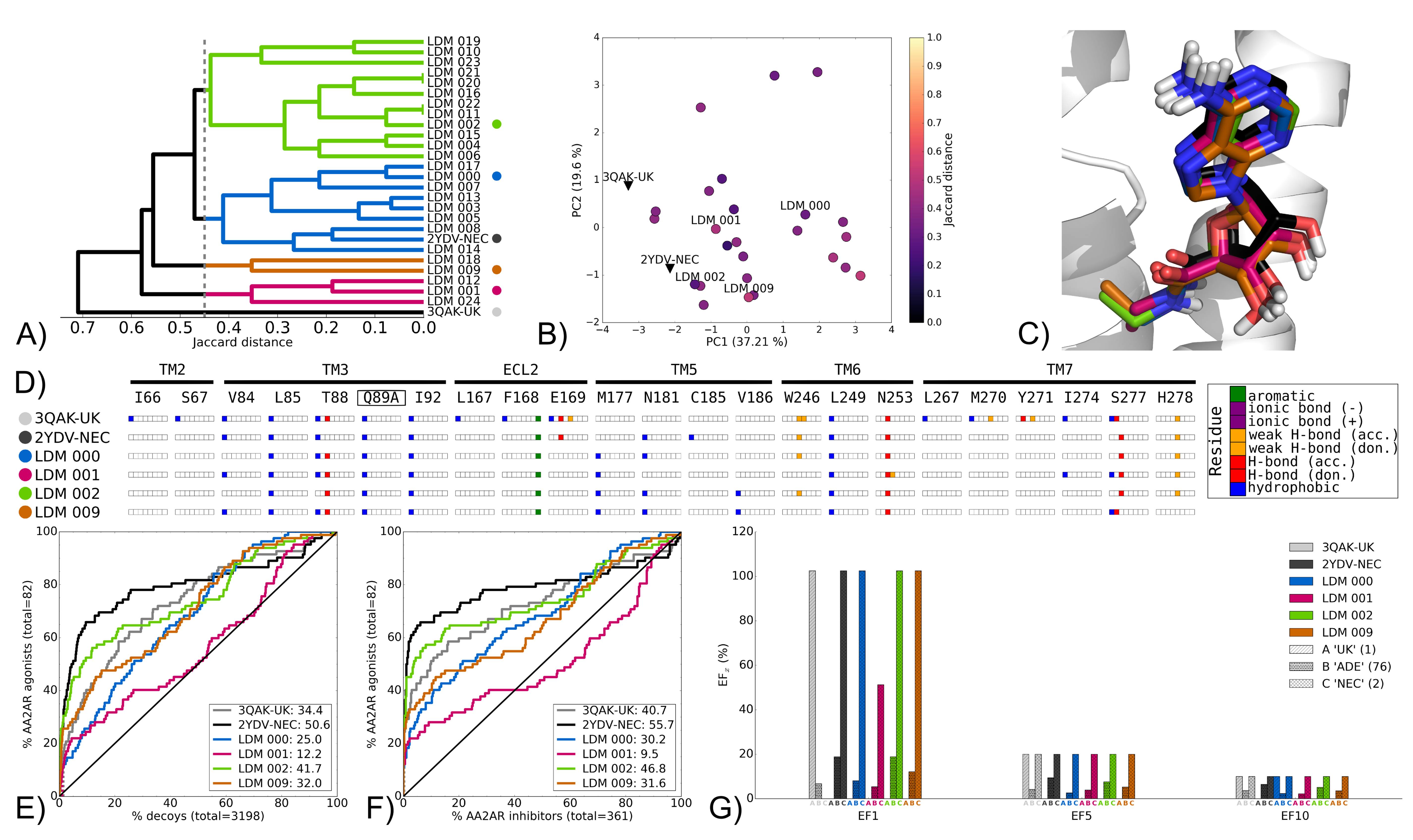

Supplement: S16 Fig — A) Dendrogram of the top 25 LDM models and X-ray structure(s), a cutoff line identifies different LDM clusters and their representative LDM models are designated by a colored dot. Representative LDM models are the highest scoring within the cluster based on the OPUS-ICM metric. B) Comparison of binding pocket conformation between the top 25 LDM models and X-ray structure(s). LDM models are colored based on their IFP Jaccard distance with the destination X-ray structure. C) Binding poses of the representative LDM model(s) and the destination X-ray structure. D) IFP of the representative LDM models and the X-ray structures. Interaction type is described for each residue of the binding pocket: hydrophobic interaction, hydrogen bond (H-bond) donor and acceptor, weak hydrogen bond (weak H-bond) donor and acceptor, ionic bond positive (+) and negative (-) and aromatic interaction. VS performance is described with ROC curves to visualise E) the recovery of known ligands vs. decoys and F) the selectivity of inhibitors over agonists (or vice-versa). The relative rank of the LDM refinement ligand is identified with a vertical dashed line. This vertical line may be masked by other curves if the ligand is very highly ranked. The ROC curve figure inset shows NSQ_AUC values for each binding pocket. Finally, a G) bar chart is used to visualise the EF for representative known ligand chemotypes at EF1, EF5 and EF10. Chemotypes A ‘UK-like’, B ‘ADE-like’ and C ‘NEC-like’ represent only a subset of AA2AR agonist ligands (S2 Fig). The EF bar chart inset shows the number of ligands for each chemotype cluster between parenthesis. Origin and destination X-ray structure chemotype EF shown in grey and black bars, respectively, with the LDM models coloured based on their relative clusters identified in A. (TIF) [file pcbi.1005819.s020.tif]

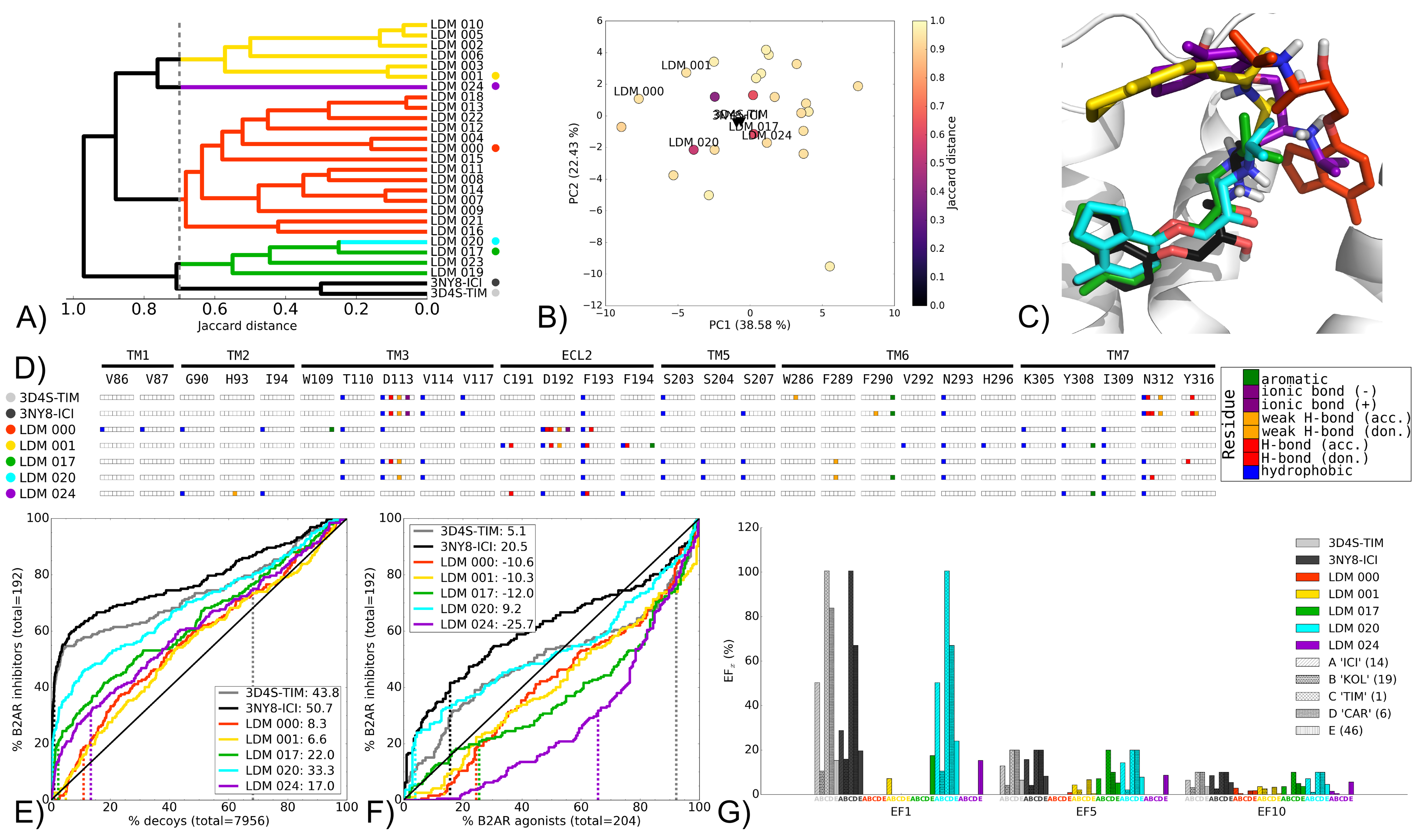

Supplement: S17 Fig — A) Dendrogram of the top 25 LDM models and X-ray structure(s), a cutoff line identifies different LDM clusters and their representative LDM models are designated by a colored dot. Representative LDM models are the highest scoring within the cluster based on the OPUS-ICM metric. B) Comparison of binding pocket conformation between the top 25 LDM models and X-ray structure(s). LDM models are colored based on their IFP Jaccard distance with the destination X-ray structure. C) Binding poses of the representative LDM model(s) and the destination X-ray structure. D) IFP of the representative LDM models and the X-ray structures. Interaction type is described for each residue of the binding pocket: hydrophobic interaction, hydrogen bond (H-bond) donor and acceptor, weak hydrogen bond (weak H-bond) donor and acceptor, ionic bond positive (+) and negative (-) and aromatic interaction. VS performance is described with ROC curves to visualise E) the recovery of known ligands vs. decoys and F) the selectivity of inhibitors over agonists (or vice-versa). The relative rank of the LDM refinement ligand is identified with a vertical dashed line. This vertical line may be masked by other curves if the ligand is very highly ranked. The ROC curve figure inset shows NSQ_AUC values for each binding pocket. Finally, a G) bar chart is used to visualise the EF for representative known ligand chemotypes at EF1, EF5 and EF10. Chemotypes A ‘ICI-like’, B ‘KOL-like’, C ‘TIM-like’, D ‘CAR-like’ and E represent only a subset of B2AR inhibitor ligands (S2 Fig). The EF bar chart inset shows the number of ligands for each chemotype cluster between parenthesis. Origin and destination X-ray structure chemotype EF shown in grey and black bars, respectively, with the LDM models coloured based on their relative clusters identified in A. (TIF) [file pcbi.1005819.s021.tif]

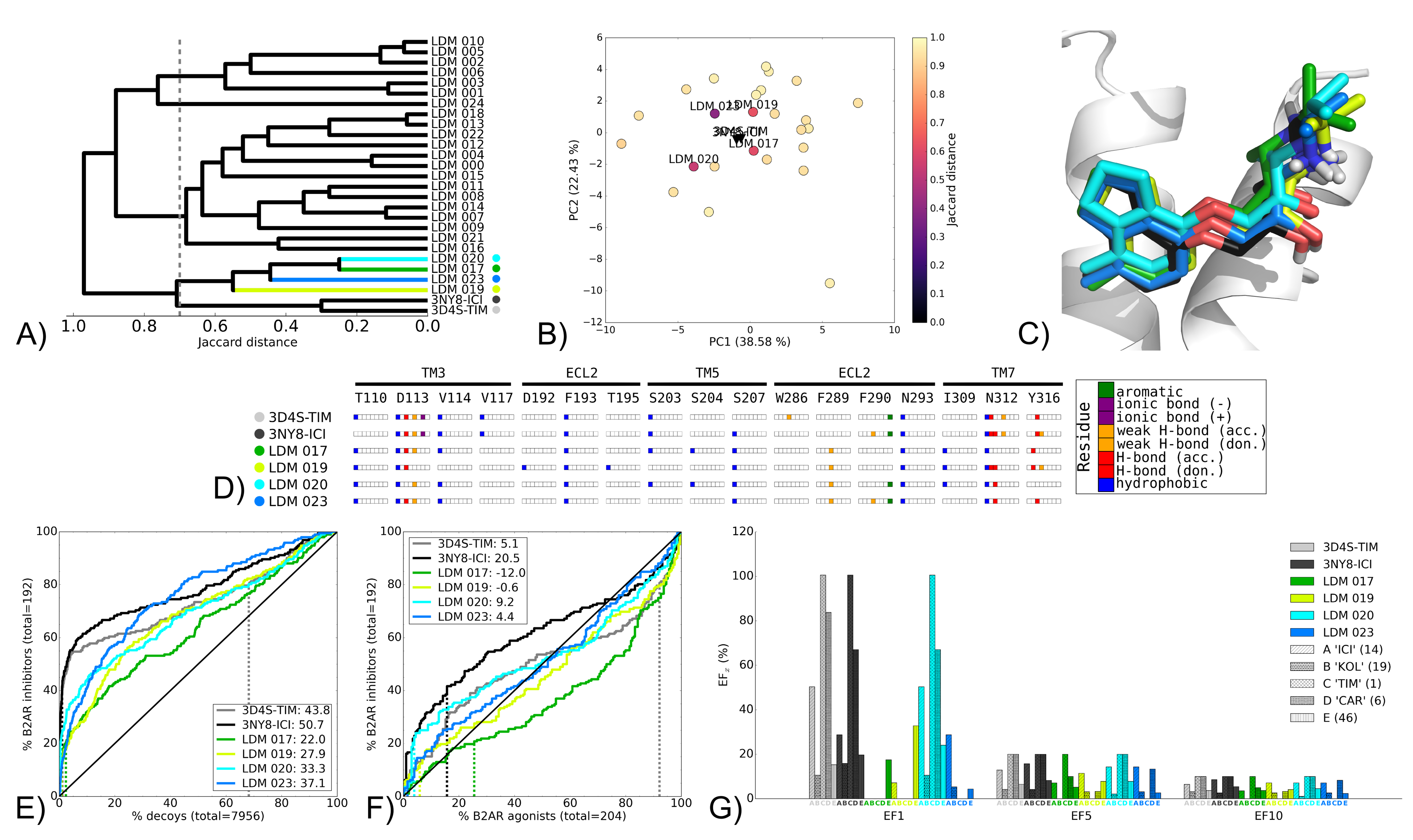

Supplement: S18 Fig — A) Dendrogram of the top 25 LDM models and X-ray structure(s), a cutoff line identifies different LDM clusters and their representative LDM models are designated by a colored dot. Representative LDM models are the highest scoring within the cluster based on the OPUS-ICM metric. B) Comparison of binding pocket conformation between the top 25 LDM models and X-ray structure(s). LDM models are colored based on their IFP Jaccard distance with the destination X-ray structure. C) Binding poses of the representative LDM model(s) and the destination X-ray structure. D) IFP of the representative LDM models and the X-ray structures. Interaction type is described for each residue of the binding pocket: hydrophobic interaction, hydrogen bond (H-bond) donor and acceptor, weak hydrogen bond (weak H-bond) donor and acceptor, ionic bond positive (+) and negative (-) and aromatic interaction. VS performance is described with ROC curves to visualise E) the recovery of known ligands vs. decoys and F) the selectivity of inhibitors over agonists (or vice-versa). The relative rank of the LDM refinement ligand is identified with a vertical dashed line. This vertical line may be masked by other curves if the ligand is very highly ranked. The ROC curve figure inset shows NSQ_AUC values for each binding pocket. Finally, a G) bar chart is used to visualise the EF for representative known ligand chemotypes at EF1, EF5 and EF10. Chemotypes A ‘ICI-like’, B ‘KOL-like’, C ‘TIM-like’, D ‘CAR-like’ and E represent only a subset of B2AR inhibitor ligands (S2 Fig). The EF bar chart inset shows the number of ligands for each chemotype cluster between parenthesis. Origin and destination X-ray structure chemotype EF shown in grey and black bars, respectively, with the LDM models coloured based on their relative clusters identified in A. (TIF) [file pcbi.1005819.s022.tif]

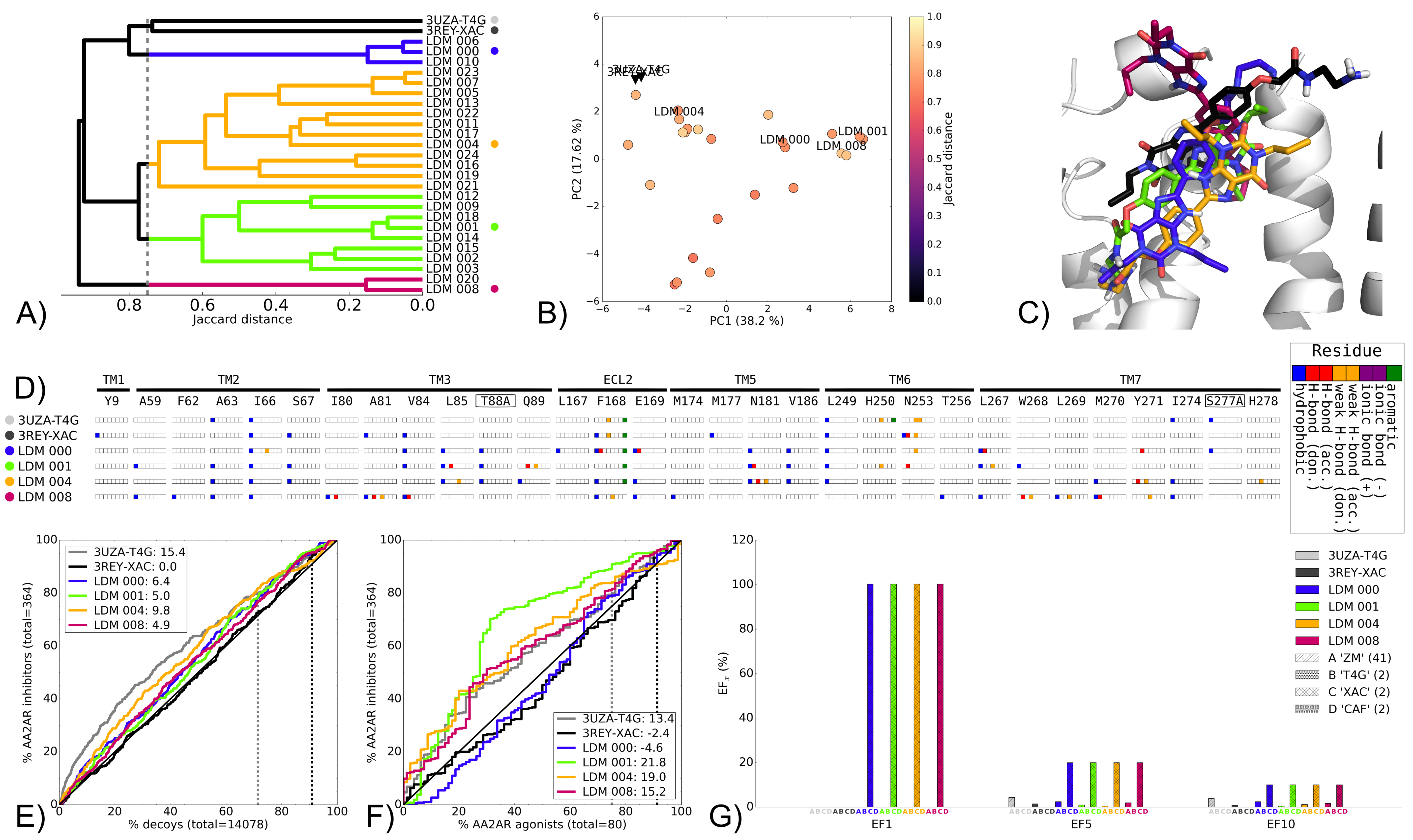

Supplement: S19 Fig — A) Dendrogram of the top 25 LDM models and X-ray structure(s), a cutoff line identifies different LDM clusters and their representative LDM models are designated by a colored dot. Representative LDM models are the highest scoring within the cluster based on the OPUS-ICM metric. B) Comparison of binding pocket conformation between the top 25 LDM models and X-ray structure(s). LDM models are colored based on their IFP Jaccard distance with the destination X-ray structure. C) Binding poses of the representative LDM model(s) and the destination X-ray structure. D) IFP of the representative LDM models and the X-ray structures. Interaction type is described for each residue of the binding pocket: hydrophobic interaction, hydrogen bond (H-bond) donor and acceptor, weak hydrogen bond (weak H-bond) donor and acceptor, ionic bond positive (+) and negative (-) and aromatic interaction. VS performance is described with ROC curves to visualise E) the recovery of known ligands vs. decoys and F) the selectivity of inhibitors over agonists (or vice-versa). The relative rank of the LDM refinement ligand is identified with a vertical dashed line. This vertical line may be masked by other curves if the ligand is very highly ranked. The ROC curve figure inset shows NSQ_AUC values for each binding pocket. Finally, a G) bar chart is used to visualise the EF for representative known ligand chemotypes at EF1, EF5 and EF10. Chemotypes A ‘ZM-like’, B ‘T4G-like’, C ‘XAC-like’ and D ‘CAF-like’ represent only a subset of AA2AR inhibitors ligands (S2 Fig). The EF bar chart inset shows the number of ligands for each chemotype cluster between parenthesis. Origin and destination X-ray structure chemotype EF shown in grey and black bars, respectively, with the LDM models coloured based on their relative clusters identified in A. (TIF) [file pcbi.1005819.s023.tif]

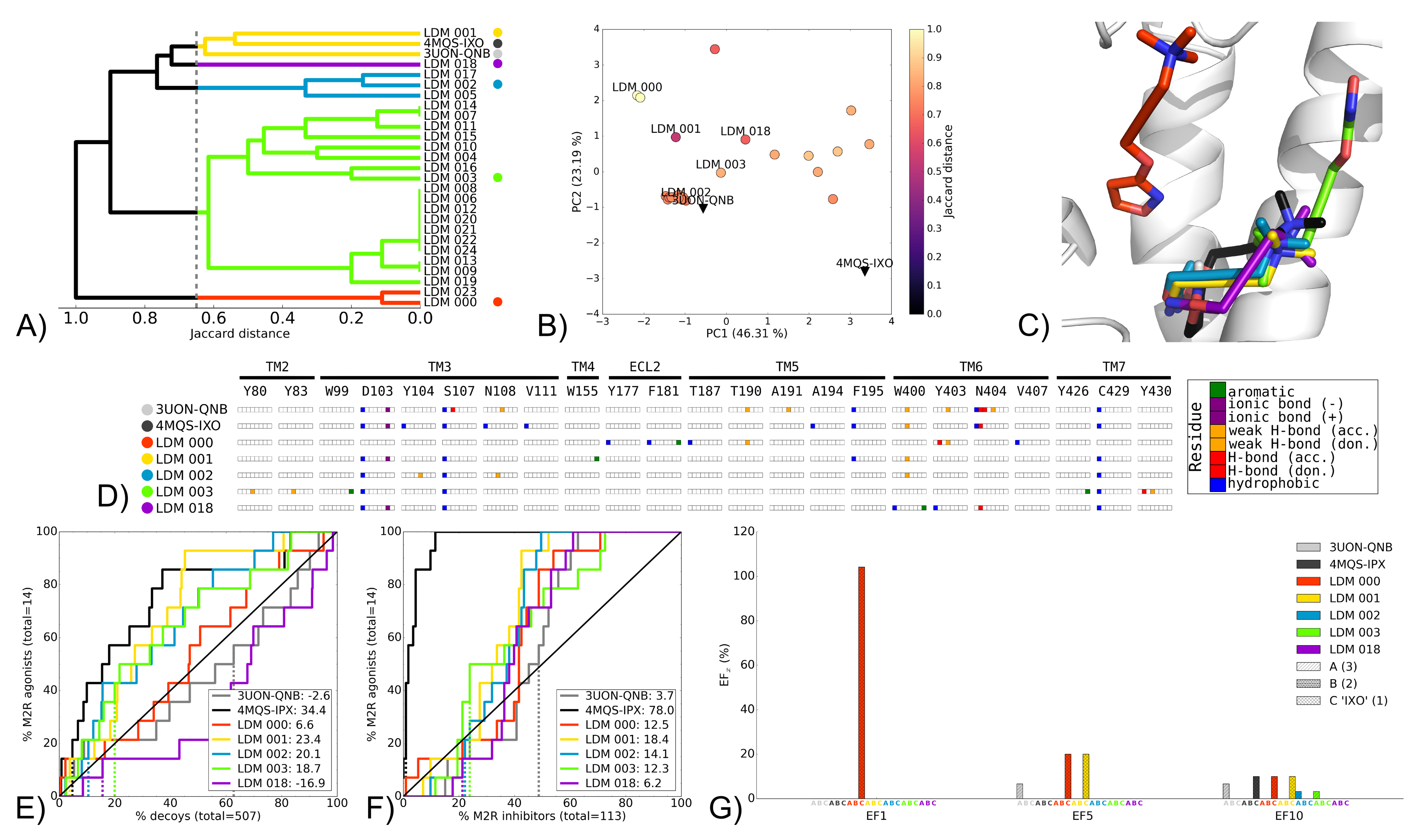

Supplement: S20 Fig — A) Dendrogram of the top 25 LDM models and X-ray structure(s), a cutoff line identifies different LDM clusters and their representative LDM models are designated by a colored dot. Representative LDM models are the highest scoring within the cluster based on the OPUS-ICM metric. B) Comparison of binding pocket conformation between the top 25 LDM models and X-ray structure(s). LDM models are colored based on their IFP Jaccard distance with the destination X-ray structure. C) Binding poses of the representative LDM model(s) and the destination X-ray structure. D) IFP of the representative LDM models and the X-ray structures. Interaction type is described for each residue of the binding pocket: hydrophobic interaction, hydrogen bond (H-bond) donor and acceptor, weak hydrogen bond (weak H-bond) donor and acceptor, ionic bond positive (+) and negative (-) and aromatic interaction. VS performance is described with ROC curves to visualise E) the recovery of known ligands vs. decoys and F) the selectivity of inhibitors over agonists (or vice-versa). The relative rank of the LDM refinement ligand is identified with a vertical dashed line. This vertical line may be masked by other curves if the ligand is very highly ranked. The ROC curve figure inset shows NSQ_AUC values for each binding pocket. Finally, a G) bar chart is used to visualise the EF for representative known ligand chemotypes at EF1, EF5 and EF10. Chemotypes A, B and C ‘IXO-like’ represent only a subset of M2R agonist ligands (S2 Fig). The EF bar chart inset shows the number of ligands for each chemotype cluster between parenthesis. Origin and destination X-ray structure chemotype EF shown in grey and black bars, respectively, with the LDM models coloured based on their relative clusters identified in A. (TIF) [file pcbi.1005819.s024.tif]

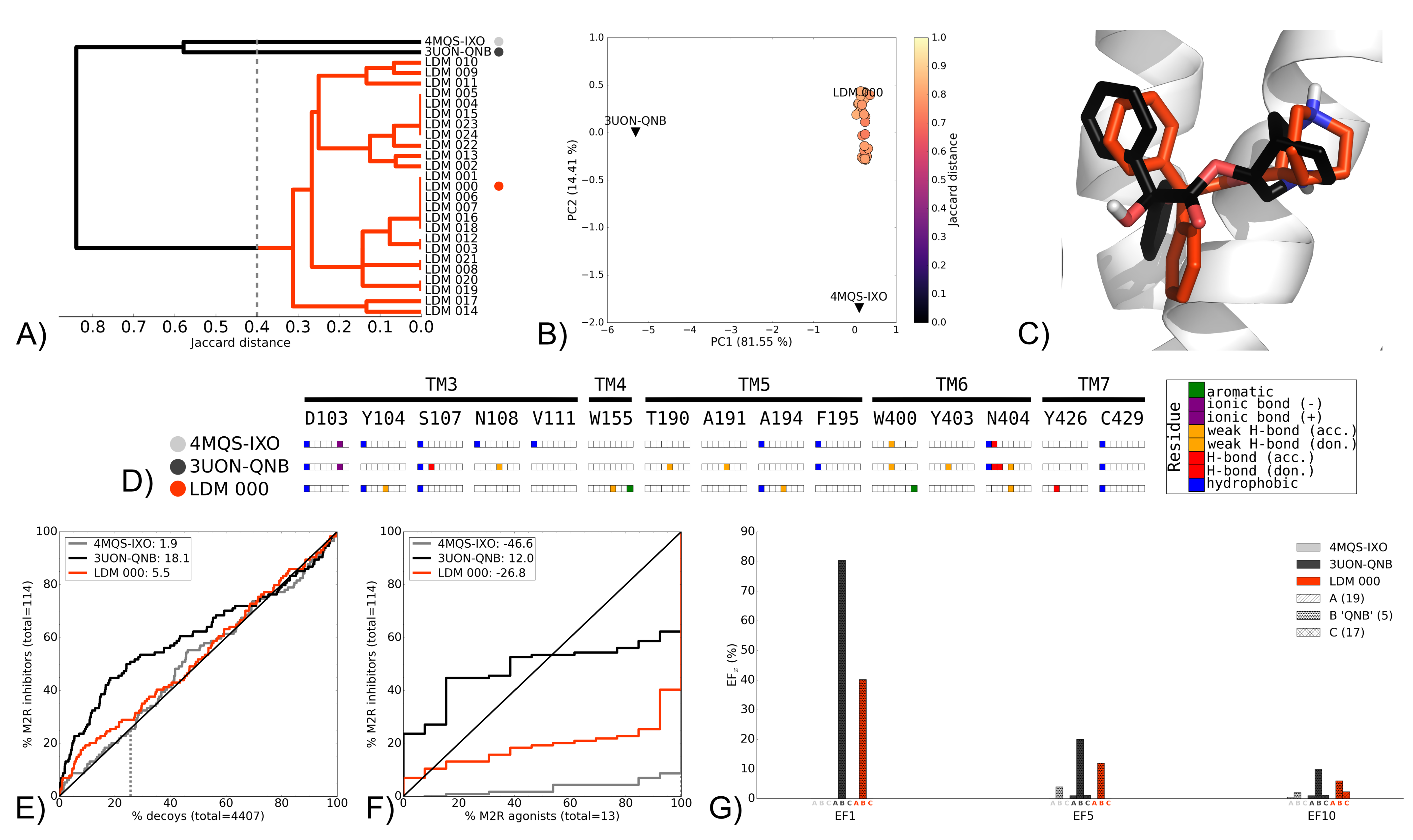

Supplement: S21 Fig — A) Dendrogram of the top 25 LDM models and X-ray structure(s), a cutoff line identifies different LDM clusters and their representative LDM models are designated by a colored dot. Representative LDM models are the highest scoring within the cluster based on the OPUS-ICM metric. B) Comparison of binding pocket conformation between the top 25 LDM models and X-ray structure(s). LDM models are colored based on their IFP Jaccard distance with the destination X-ray structure. C) Binding poses of the representative LDM model(s) and the destination X-ray structure. D) IFP of the representative LDM models and the X-ray structures. Interaction type is described for each residue of the binding pocket: hydrophobic interaction, hydrogen bond (H-bond) donor and acceptor, weak hydrogen bond (weak H-bond) donor and acceptor, ionic bond positive (+) and negative (-) and aromatic interaction. VS performance is described with ROC curves to visualise E) the recovery of known ligands vs. decoys and F) the selectivity of inhibitors over agonists (or vice-versa). The relative rank of the LDM refinement ligand is identified with a vertical dashed line. This vertical line may be masked by other curves if the ligand is very highly ranked. The ROC curve figure inset shows NSQ_AUC values for each binding pocket. Finally, a G) bar chart is used to visualise the EF for representative known ligand chemotypes at EF1, EF5 and EF10. Chemotypes A, B ‘QNB-like’ and C represent only a subset of M2R inhibitor ligands (S2 Fig). The EF bar chart inset shows the number of ligands for each chemotype cluster between parenthesis. Origin and destination X-ray structure chemotype EF shown in grey and black bars, respectively, with the LDM models coloured based on their relative clusters identified in A. (TIF) [file pcbi.1005819.s025.tif]

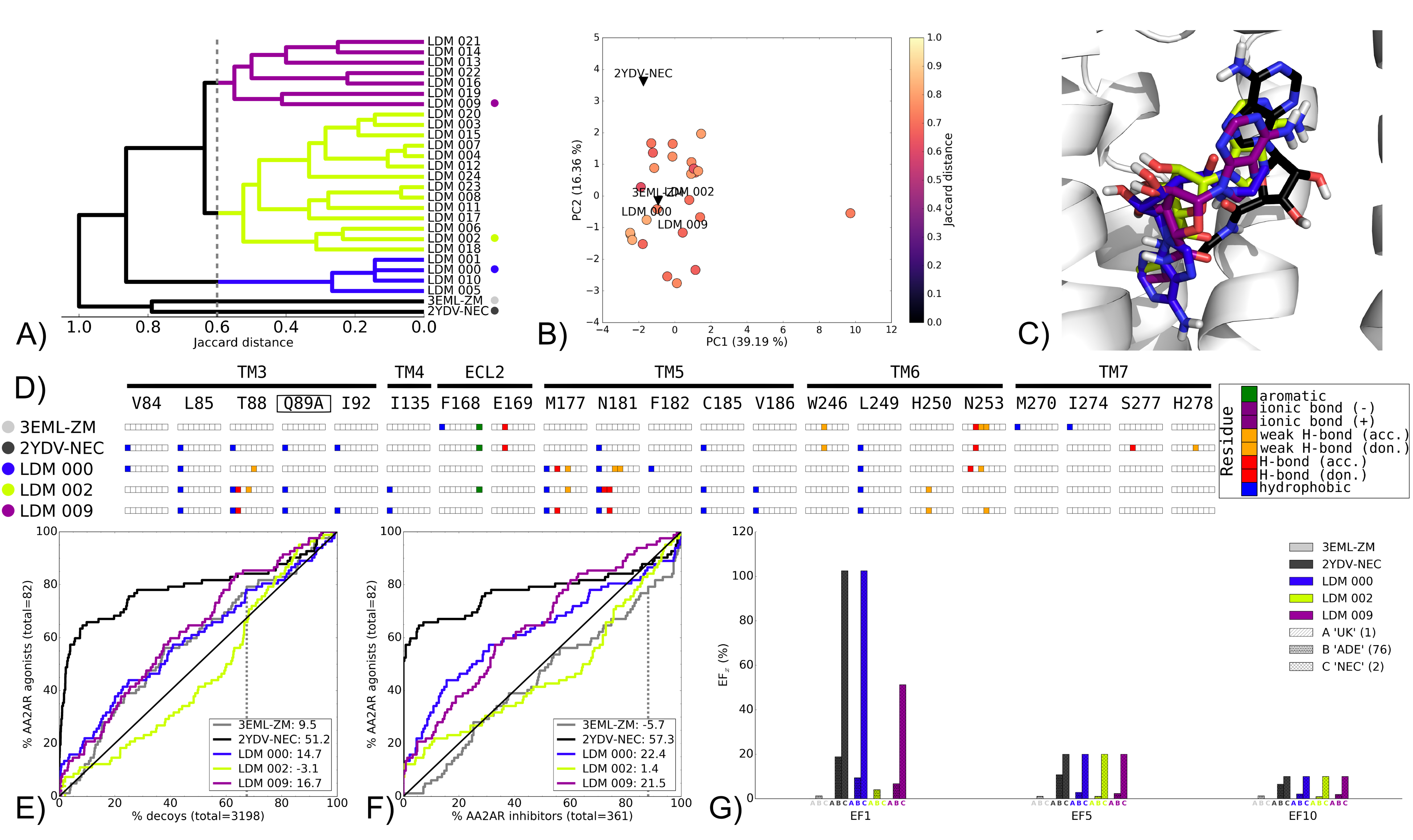

Supplement: S22 Fig — A) Dendrogram of the top 25 LDM models and X-ray structure(s), a cutoff line identifies different LDM clusters and their representative LDM models are designated by a colored dot. Representative LDM models are the highest scoring within the cluster based on the OPUS-ICM metric. B) Comparison of binding pocket conformation between the top 25 LDM models and X-ray structure(s). LDM models are colored based on their IFP Jaccard distance with the destination X-ray structure. C) Binding poses of the representative LDM model(s) and the destination X-ray structure. D) IFP of the representative LDM models and the X-ray structures. Interaction type is described for each residue of the binding pocket: hydrophobic interaction, hydrogen bond (H-bond) donor and acceptor, weak hydrogen bond (weak H-bond) donor and acceptor, ionic bond positive (+) and negative (-) and aromatic interaction. VS performance is described with ROC curves to visualise E) the recovery of known ligands vs. decoys and F) the selectivity of inhibitors over agonists (or vice-versa). The relative rank of the LDM refinement ligand is identified with a vertical dashed line. This vertical line may be masked by other curves if the ligand is very highly ranked. The ROC curve figure inset shows NSQ_AUC values for each binding pocket. Finally, a G) bar chart is used to visualise the EF for representative known ligand chemotypes at EF1, EF5 and EF10. Chemotypes A ‘UK-like’, B ‘ADE-like’ and C ‘NEC-like’ represent only a subset of AA2AR agonist ligands (S2 Fig). The EF bar chart inset shows the number of ligands for each chemotype cluster between parenthesis. Origin and destination X-ray structure chemotype EF shown in grey and black bars, respectively, with the LDM models coloured based on their relative clusters identified in A. (TIF) [file pcbi.1005819.s026.tif]

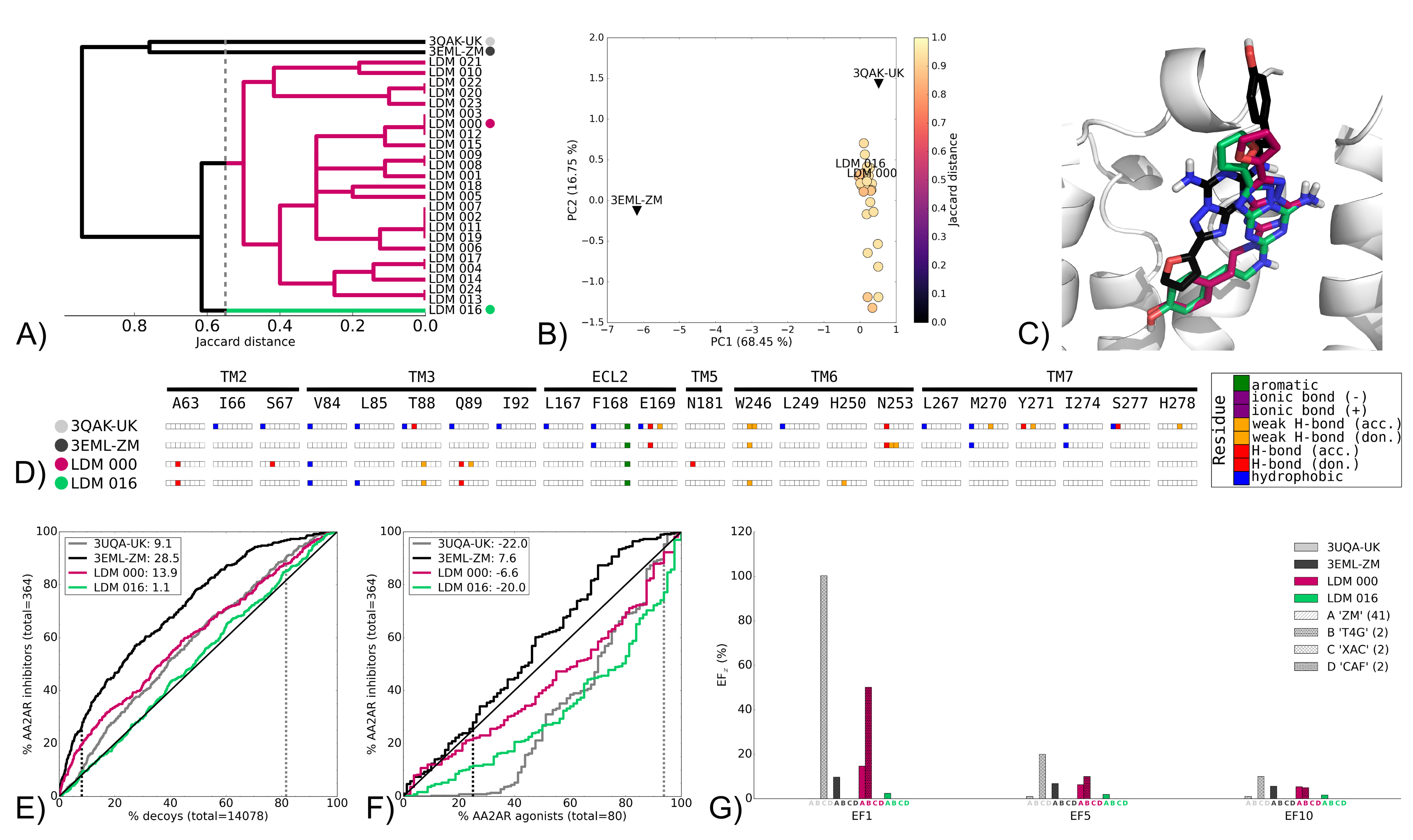

Supplement: S23 Fig — A) Dendrogram of the top 25 LDM models and X-ray structure(s), a cutoff line identifies different LDM clusters and their representative LDM models are designated by a colored dot. Representative LDM models are the highest scoring within the cluster based on the OPUS-ICM metric. B) Comparison of binding pocket conformation between the top 25 LDM models and X-ray structure(s). LDM models are colored based on their IFP Jaccard distance with the destination X-ray structure. C) Binding poses of the representative LDM model(s) and the destination X-ray structure. D) IFP of the representative LDM models and the X-ray structures. Interaction type is described for each residue of the binding pocket: hydrophobic interaction, hydrogen bond (H-bond) donor and acceptor, weak hydrogen bond (weak H-bond) donor and acceptor, ionic bond positive (+) and negative (-) and aromatic interaction. VS performance is described with ROC curves to visualise E) the recovery of known ligands vs. decoys and F) the selectivity of inhibitors over agonists (or vice-versa). The relative rank of the LDM refinement ligand is identified with a vertical dashed line. This vertical line may be masked by other curves if the ligand is very highly ranked. The ROC curve figure inset shows NSQ_AUC values for each binding pocket. Finally, a G) bar chart is used to visualise the EF for representative known ligand chemotypes at EF1, EF5 and EF10. Chemotypes A ‘ZM-like’, B ‘T4G-like’, C ‘XAC-like’ and D ‘CAF-like’ represent only a subset of AA2AR inhibitors ligands (S2 Fig). The EF bar chart inset shows the number of ligands for each chemotype cluster between parenthesis. Origin and destination X-ray structure chemotype EF shown in grey and black bars, respectively, with the LDM models coloured based on their relative clusters identified in A. (TIF) [file pcbi.1005819.s027.tif]
