# Supplementary material for: Improving virtual screening of G protein-coupled receptors via ligand-directed modeling
Source: PLoS Comput Biol. 2017 Nov 13;13(11):e1005819. doi: 10.1371/journal.pcbi.1005819 (PMC5708846; doi:10.1371/journal.pcbi.1005819)
Supplement: S2 Fig — Ligand libraries were clustered based on pharmacophore properties and 0.5 cutoff was used to identify the different clusters. A limited number of representative chemotype clusters were identified by letters (A-E). Chemotype clusters that contain an X-ray ligand were chosen and additional populated clusters were added when increased chemotype coverage was necessary. The resulting chemotype clusters do not contain the entire ligand library but are used to represent its chemotype diversity. AA2AR agonists (A) and inhibitors (B), B2AR agonists (C) and inhibitors (D), M2R agonists (E) and inhibitors (F), CCR5 inhibitors (G), DOR inhibitors (H), H1R inhibitors (I), 5-HT1B inhibitors (J). (PDF) [file pcbi.1005819.s006.pdf]

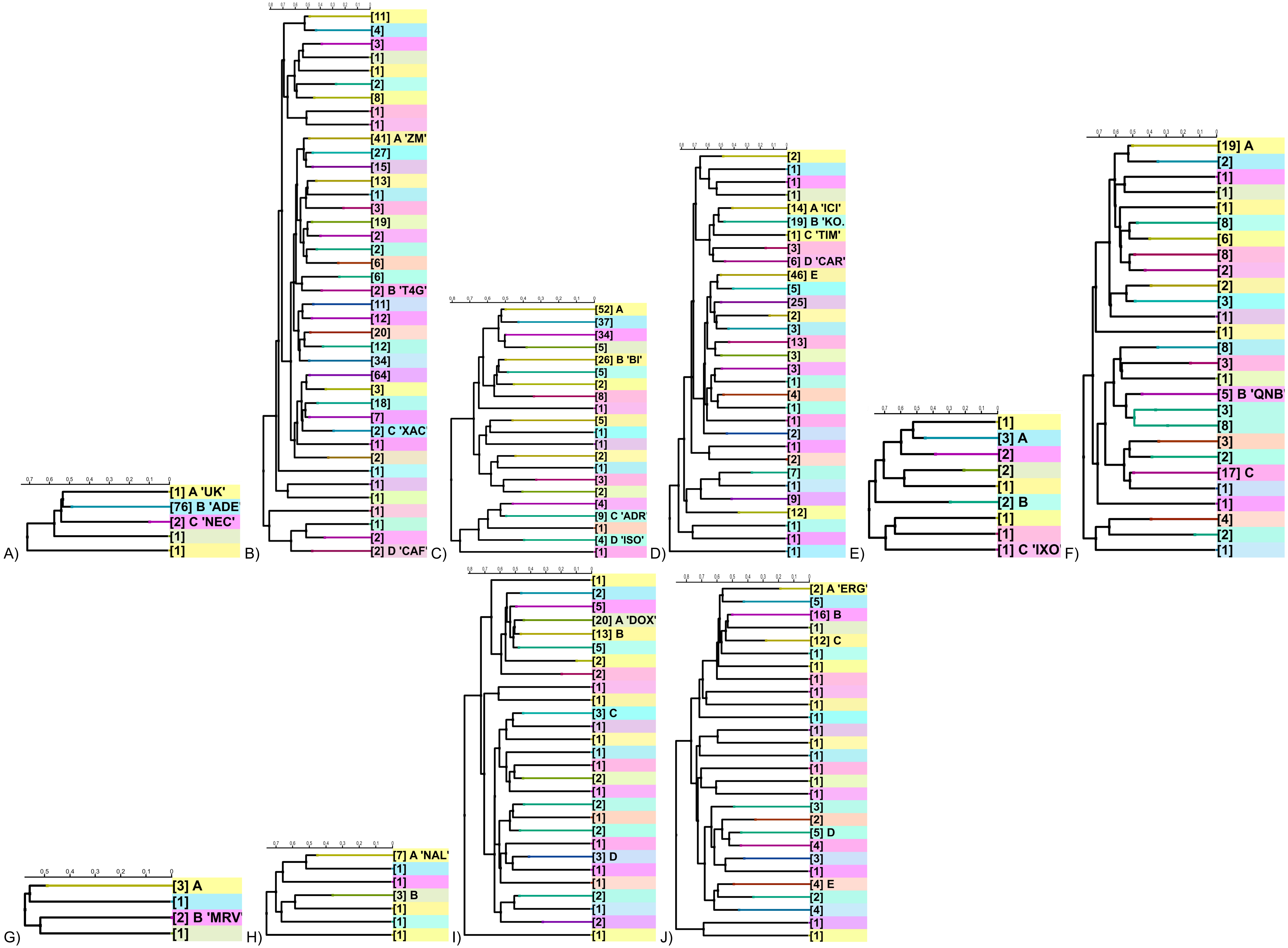
A)
[1] A 'UK'
[76] B 'ADE'
[2] C 'NEC'
[1]
[1]
B)
[11]
[4]
[3]
[1]
[1]
[2]
[8]
[1]
[1]
[41] A 'ZM'
[27]
[15]
[13]
[1]
[3]
[19]
[2]
[2]
[6]
[6]
[2] B 'T4G'
[11]
[12]
[20]
[12]
[34]
[64]
[3]
[18]
[7]
[2] C 'XAC'
[1]
[2]
[1]
[1]
[1]
[1]
[1]
[2]
[2] D 'CAF'
C)
[52] A
[37]
[34]
[5]
[26] B 'BI'
[5]
[2]
[8]
[1]
[5]
[1]
[1]
[2]
[1]
[3]
[2]
[4]
[9] C 'ADR'
[1]
[4] D 'ISO'
[1]
D)
[2]
[1]
[1]
[1]
[14] A 'ICI'
[19] B 'KO.
[1] C 'TIM'
[3]
[6] D 'CAR'
[46] E
[5]
[25]
[2]
[3]
[13]
[3]
[3]
[1]
[4]
[1]
[1]
[2]
[1]
[2]
[7]
[1]
[9]
[12]
[1]
[1]
[1]
E)
[1]
[3] A
[2]
[2]
[1]
[2] B
[1]
[1]
[1] C 'IXO'
F)
[19] A
[2]
[1]
[1]
[1]
[8]
[6]
[8]
[2]
[2]
[3]
[1]
[1]
[8]
[3]
[1]
[5] B 'QNB'
[3]
[8]
[3]
[2]
[17] C
[1]
[1]
[4]
[2]
[1]
G)
[3] A
[1]
[2] B 'MRV'
[1]
H)
[7] A 'NAL'
[1]
[1]
[3] B
[1]
[1]
[1]
I)
[1]
[2]
[5]
[20] A 'DOX'
[13] B
[5]
[2]
[2]
[1]
[1]
[3] C
[1]
[1]
[1]
[1]
[2]
[1]
[2]
[1]
[2]
[1]
[3] D
[1]
[1]
[2]
[1]
[2]
[1]
J)
[2] A 'ERG'
[5]
[16] B
[1]
[12] C
[1]
[1]
[1]
[1]
[1]
[1]
[1]
[1]
[1]
[1]
[1]
[1]
[3]
[2]
[5] D
[4]
[3]
[1]
[4] E
[2]
[4]
[1]
[1]
